# Yellow, Red, and Black *Lepidium meyenii* Preparations Differentially Modulate Doxorubicin Response and Lipid Metabolism-Related Changes in Pancreatic Cancer Cells

**DOI:** 10.3390/molecules31214011

**Published:** 2026-11-06

**Authors:** Adam Jan Olichwier, Izabela Suwda Kalita, Magdalena Bruzgo-Grzybko, Aleksandra Golonko, Natalia Bielicka, Tomasz Krzysztof Bednarski, Ewa Chabielska, Anna Gromotowicz-Poplawska

**Affiliations:** 1 Radiopharmacy Centre, Medical University of Bialystok, 15-569 Bialystok, Poland; izabela.kalita@umb.edu.pl (I.S.K.); anna.gromotowicz-poplawska@umb.edu.pl (A.G.-P.); 2 Department of Biopharmacy and Radiopharmacy, Medical University of Bialystok, 15-222 Bialystok, Poland; magdalena.bruzgo-grzybko@umb.edu.pl (M.B.-G.); natalia.marcinczyk@umb.edu.pl (N.B.); ewa.chabielska@umb.edu.pl (E.C.); 3 Clinical Research Center, Medical University of Bialystok, 15-276 Bialystok, Poland; aleksandra.golonko@umb.edu.pl; 4 Department of Nutrition and Health Sciences, University of Nebraska, Lincoln, NE 68503, USA; tomasz.k.bednarski@unl.edu

**Keywords:** pancreatic cancer, *Lepidium meyenii*, maca, doxorubicin, natural products, drug interaction, lipid metabolism, fibroblast activation protein, apoptosis

## Abstract

Pancreatic cancer is characterized by cellular heterogeneity, contributing to variable responses to anticancer treatment. Natural products may modify these responses in a context-dependent manner. We investigated whether three *Lepidium meyenii* Walp. (Brassicaceae; maca) preparations, marked as yellow (MY), red (MR), and black (MB), differentially modify pancreatic cancer cell responses to doxorubicin (DOX). BxPC-3, MIA PaCa-2, and PANC-1 cells were treated with maca preparations alone or combined with DOX. Cell viability, apoptosis-associated signaling, survival-related proteins, lipid droplet accumulation, and lipid metabolism-related proteins were evaluated. Drug interactions were assessed using Bliss independence and Highest Single Agent (HSA) models. Interaction profiles differed among cell lines and depended on the tested maca preparation and DOX concentration. The strongest favorable interactions were observed in PANC-1 cells treated with MR or MB plus 2.5 µM DOX (Bliss/HSA scores: 18.9/13.1 and 17.6/12.4, respectively), whereas BxPC-3 cells showed favorable interactions with MY, MR, and MB at 5 µM DOX (Bliss/HSA scores: 12.4/9.8, 10.7/8.5, and 11.9/9.2, respectively). MIA PaCa-2 cells showed mainly neutral or less-than-additive interactions. These responses were accompanied by changes in caspase-3 activation, PI3K, FAP, and FOXO1 protein levels, lipid droplet accumulation, and ATGL, ABHD5, and PLIN3 levels. Overall, the tested MY, MR, and MB preparations differentially modified pancreatic cancer cell responses to DOX and were associated with distinct survival-related and lipid metabolism-related changes. Further phytochemical characterization and functional mechanistic studies are required to identify the constituents involved and determine whether the observed molecular changes contribute causally to these effects.

## 1. Introduction

Pancreatic cancer remains one of the most lethal malignancies worldwide, with persistently poor long-term survival despite advances in diagnosis and treatment [1,2]. Its unfavorable prognosis is largely attributed to late diagnosis, rapid metastatic progression, and pronounced resistance to systemic therapies. A key feature of pancreatic cancer is its substantial metabolic plasticity, which enables tumor cells to adapt to therapeutic stress through the activation of pro-survival signaling pathways and metabolic reprogramming [3,4]. These adaptive mechanisms can support cancer cell survival under treatment-induced stress and may therefore represent potential targets for improving therapeutic responses.

The BxPC-3, MIA PaCa-2, and PANC-1 pancreatic cancer cell lines provide complementary experimental models because they differ in their molecular characteristics and therapeutic responsiveness [5,6]. BxPC-3 cells are Kirsten rat sarcoma viral oncogene homolog (KRAS) wild-type, whereas MIA PaCa-2 and PANC-1 cells harbor activating KRAS mutations [7,8]. These mutations are associated with constitutive activation of downstream signaling pathways, including mitogen-activated protein kinase (MAPK) and phosphatidylinositol 3-kinase/protein kinase B (PI3K/AKT), which contribute to tumor cell survival, metabolic adaptation, and resistance to therapeutic stress [7,8].

Doxorubicin (DOX) is a well-established chemotherapeutic agent that induces DNA damage, oxidative stress, and apoptosis [9]. In addition to its direct cytotoxic effects, DOX can influence signaling pathways involved in cell survival and metabolic regulation, including the PI3K/AKT pathway [9]. Although DOX is not a standard first-line treatment for pancreatic cancer, it was selected in the present study as a well-characterized experimental inducer of genotoxic, oxidative, and cellular stress rather than as a model of current clinical treatment. Its use provided a defined experimental context for evaluating whether maca preparations modify pancreatic cancer cell responses to anticancer drug-induced stress. Caspase-3 (CASP3) is a major executioner of apoptosis [10], whereas fibroblast activation protein (FAP) and forkhead box protein O1 (FOXO1) have been implicated in tumor progression, cellular stress responses, and therapeutic response [11,12]. Together, these molecular components provide complementary indicators of apoptosis-associated and survival-related responses to treatment-induced cellular stress.

Metabolic reprogramming is increasingly recognized as an important determinant of pancreatic cancer progression and therapeutic resistance [13]. Lipid droplets (LDs) serve as dynamic intracellular lipid reservoirs that provide energy substrates and membrane precursors, thereby supporting cancer cell adaptation to metabolic stress [14]. LD turnover is regulated by proteins involved in lipid storage and mobilization, including adipose triglyceride lipase (ATGL), α/β-hydrolase domain-containing protein 5 (ABHD5), and perilipin 3 (PLIN3) [15]. Alterations in LD homeostasis may therefore influence the ability of pancreatic cancer cells to adapt to treatment-induced stress and have been proposed as a potential target for modulating therapeutic response [14,16].

Natural products are increasingly being investigated as potential modulators of cellular responses to anticancer treatment. *Lepidium meyenii* Walp. (maca), a plant of the Brassicaceae family native to the Peruvian Andes, has traditionally been used as a food and for its health-promoting properties [17,18]. Maca is characterized by a complex phytochemical composition that includes macamides, macaenes, glucosinolates, alkaloids, polyphenols, sterols, and other secondary metabolites [17,18,19,20,21,22,23]. Importantly, its chemical profile may vary depending on plant phenotype, geographical origin, cultivation conditions, and post-harvest processing [17,18]. Maca occurs in several color phenotypes, among which yellow (MY), red (MR), and black (MB) are the most extensively studied, and differences in their chemical composition have been associated with distinct biological activities [18]. Previous studies have reported antioxidant, anti-inflammatory, metabolic regulatory, and other biological effects of maca and its constituents [17,18,19,20,21,22,23,24,25,26]. In particular, maca preparations have been shown to influence glucose and lipid metabolism and signaling pathways involved in cellular metabolic regulation [24,25,26]. However, whether MY, MR, and MB preparations differentially modulate the response of pancreatic cancer cells to anticancer drug-induced stress and whether such responses are associated with alterations in survival signaling and lipid metabolism remain largely unexplored.

Therefore, this study investigated whether three commercially available *Lepidium meyenii* preparations, marketed as MY, MR, and MB, differentially modulate pancreatic cancer cell responses to DOX. Using BxPC-3, MIA PaCa-2, and PANC-1 cell models, we evaluated the effects of MY, MR, and MB preparations, alone and in combination with DOX, on cell viability, functional wound-closure responses, apoptosis-associated signaling, survival-related proteins, LD accumulation, and lipid metabolism-related proteins. Drug interactions were further assessed using Bliss independence and Highest Single Agent (HSA) models to distinguish favorable from neutral or less-than-additive responses. By integrating these analyses, we aimed to determine whether the effects of the tested maca preparations on DOX response vary according to preparation and cellular context and whether these responses are associated with alterations in survival signaling and lipid metabolism.

## 2. Results

### 2.1. Radiological Quality Assessment of Commercial Maca Preparations

**Figure 1:**
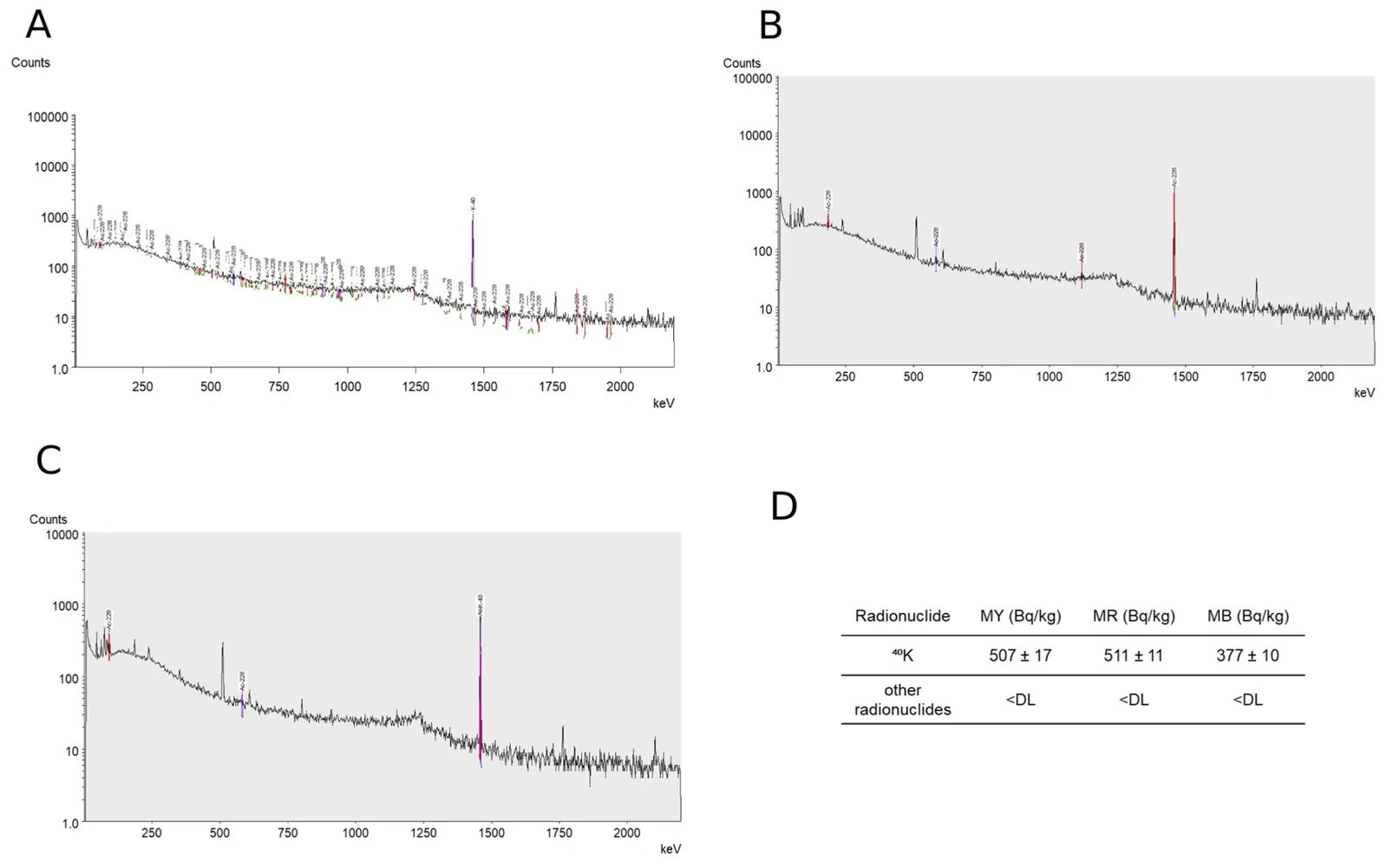
Gamma-ray spectra of maca preparations obtained using a high-purity germanium (HPGe) detector. Representative spectra of (**A**) Maca Yellow (MY), (**B**) Maca Red (MR), and (**C**) Maca Black (MB) are shown. The naturally occurring radionuclide ^40^K was detected, while the remaining analyzed radionuclides were below their corresponding detection limits (DLs). (**D**) ^40^K activity concentrations are expressed as best estimate ± 1σ total uncertainty according to ISO 11929 [27]. Sample-specific DLs varied among MY, MR, and MB; the ranges below represent the minimum–maximum DL values across the three preparations: ^40^K, 13.32–23.30 Bq/kg; ^234^Th, 10.50–15.17 Bq/kg; ^234m^Pa, 73.90–114.88 Bq/kg; ^230^Th, 28.75–53.95 Bq/kg; ^226^Ra, 11.27–15.98 Bq/kg; ^214^Pb, 1.17–19.80 Bq/kg; ^214^Bi, 6.61–7.57 Bq/kg; ^210^Pb, 17.20–29.09 Bq/kg; ^235^U, 5.23–8.41 Bq/kg; ^227^Th, 2.07–27.55 Bq/kg; ^223^Ra, 3.26–3.56 Bq/kg; ^219^Rn, 1.09–1.69 Bq/kg; ^211^Pb, 1.05–1.44 Bq/kg; ^211^Bi, 3.20–5.68 Bq/kg; ^228^Ac, 2.80–25.32 Bq/kg; ^224^Ra, 8.51–13.07 Bq/kg; ^212^Pb, 1.09–1.37 Bq/kg; ^212^Bi, 6.67–10.15 Bq/kg; ^208^Tl, 7.55–10.15 Bq/kg; and ^137^Cs, 0.36–0.69 Bq/kg. Values below the corresponding sample-specific DL were considered not detected.

*Lepidium meyenii* is traditionally cultivated at high altitudes in the Peruvian Andes, typically between approximately 3500 and 4500 m above sea level, where plants are exposed to distinctive environmental conditions [17]. Given the increased exposure to cosmic radiation at high altitudes and the potential influence of local geological conditions on plant radionuclide profiles, radiological characterization was included as an additional quality-control measure to screen for potential radioactive contamination prior to biological evaluation. Commercially available morphotype-specific maca preparations were subjected to high-purity germanium (HPGe) gamma-ray spectrometry. Only naturally occurring ^40^K was detected, whereas no artificial or anthropogenic radionuclides were identified within the sensitivity limits of the method (Figure 1A–D). These results indicated no detectable contamination with anthropogenic radionuclides in the tested maca preparations.

### 2.2. Maca Preparations Exhibit Preparation-, Cell Line-, and Time-Dependent Effects on Pancreatic Cancer Cell Viability

**Figure 2:**
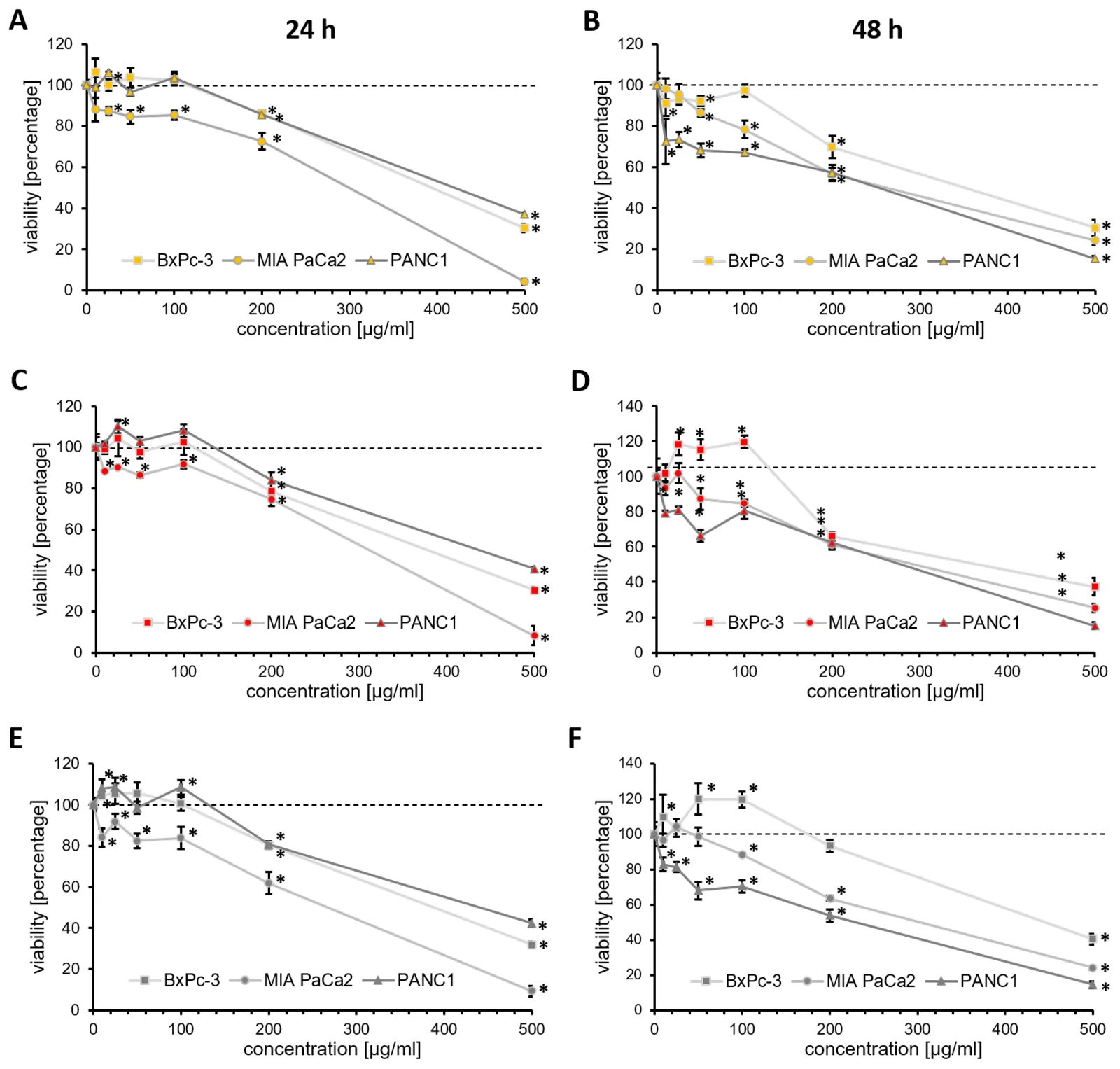
Viability of pancreatic cancer cells following treatment with Maca Yellow (MY, (**A**,**B**)), Maca Red (MR, (**C**,**D**)), and Maca Black (MB, **E**,**F**). BxPC-3, MIA PaCa-2, and PANC-1 cells were treated with increasing concentrations of MY, MR, or MB for 24 h (**A**,**C**,**E**) or 48 h (**B**,**D**,**F**). Cell viability was assessed following treatment. Data are presented as mean ± SD from three independent biological experiments, each performed with four technical replicates (*n* = 3 biological experiments). * *p* < 0.05 vs. 0 µg/mL MY/MR/MB.

Maca preparations exerted generally limited effects on pancreatic cancer cell viability, with responses varying according to cell line, maca preparation, concentration, and treatment duration (Figure 2A–F). Among the three preparations, MR produced the most pronounced reduction in cell viability, whereas the effects of MY and MB were generally more modest and became evident primarily at higher concentrations.

MIA PaCa-2 cells showed the greatest overall sensitivity to maca preparations, with viability decreasing in a concentration-dependent manner following treatment with MY (Figure 2A,B), MR (Figure 2C,D), and MB (Figure 2E,F) at both 24 and 48 h. BxPC-3 cells were comparatively less responsive, with significant reductions in viability generally observed only at concentrations ≥ 200 µg/mL. PANC-1 cells showed moderate responses after 24 h, followed by greater reductions in viability after 48 h, particularly following treatment with MY (Figure 2B) and MB (Figure 2F). At lower MR concentrations, a slight increase in BxPC-3 cell viability was observed after 48 h (Figure 2D), indicating a non-monotonic concentration-response pattern.

The calculated IC_25_ and IC_50_ values reflected these concentration- and time-dependent differences (Table 1).

### 2.3. Maca Preparations Differentially Modify Cell Viability in Combination with DOX

**Figure 3:**
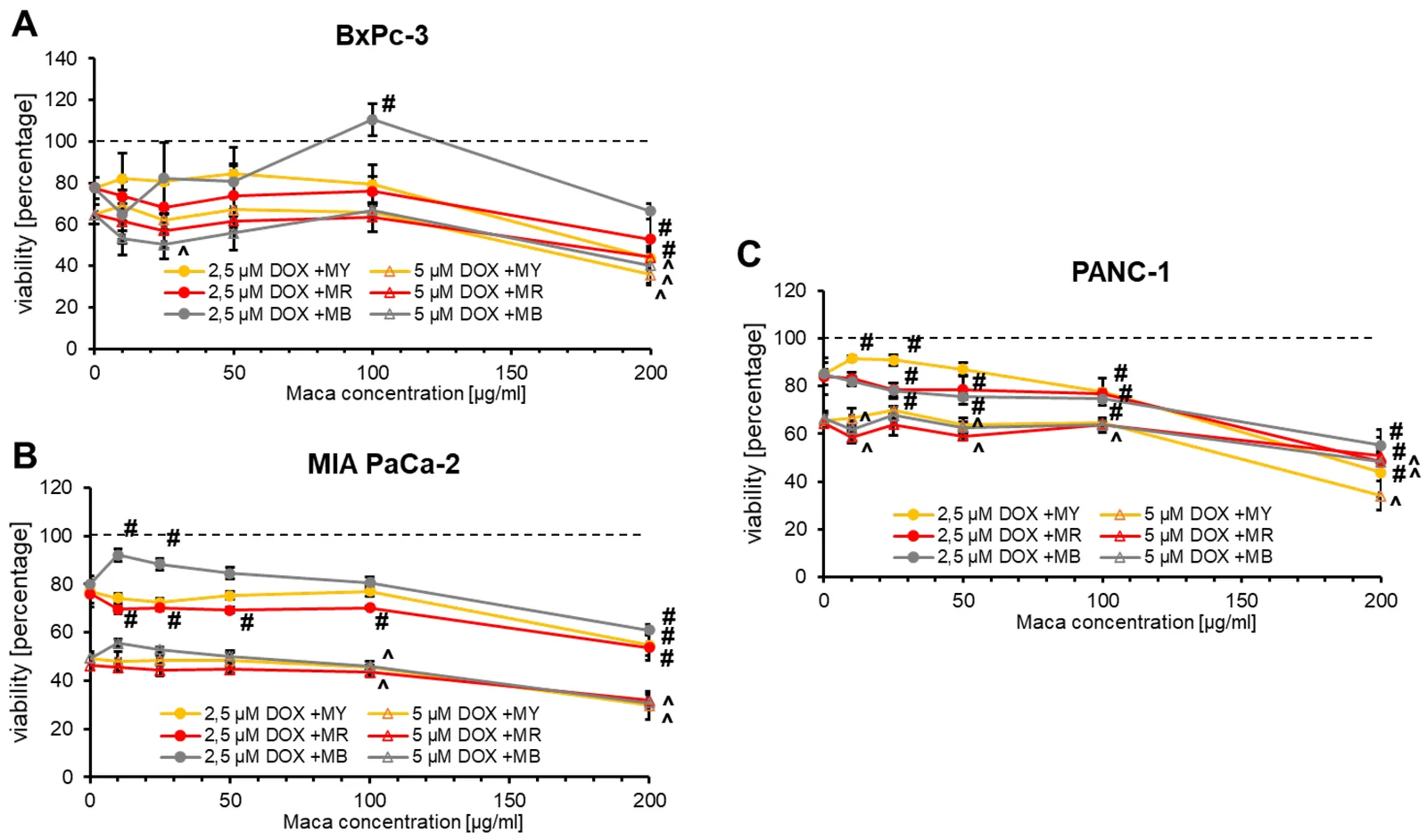
Viability of pancreatic cancer cells following DOX–maca co-treatment. BxPC-3 (**A**), MIA PaCa-2 (**B**), and PANC-1 (**C**) cells were co-treated with DOX (2.5 or 5 µM) and increasing concentrations of Maca Yellow (MY), Maca Red (MR), or Maca Black (MB). The concentrations on the *x*-axis refer to maca preparations, with 0 µg/mL representing DOX monotherapy (2.5 or 5 µM). Cell viability was expressed relative to the untreated control (100%), indicated by the dashed horizontal line, without additional normalization to DOX monotherapy. Data are presented as mean ± SD from three independent biological experiments, each performed with four technical replicates (*n* = 3 biological experiments). # *p* < 0.05 vs. DOX 2.5 µM alone; ^ *p* < 0.05 vs. DOX 5 µM alone.

Dose-response analysis showed a concentration-dependent reduction in cell viability following DOX treatment in all three pancreatic cancer cell lines (Supplementary Figure S1). Based on these dose–response curves, two common DOX concentrations (2.5 and 5 µM) were selected for combination experiments to enable direct comparison across the three cell lines (Supplementary Table S1). All combination treatments were performed for 24 h. In Figure 3, the concentrations on the *x*-axis refer exclusively to maca preparations, with 0 µg/mL representing DOX monotherapy at the corresponding concentration (2.5 or 5 µM).

Co-treatment with maca preparations and DOX resulted in preparation- and cell line-dependent changes in cell viability (Figure 3A–C). MR produced the most pronounced reductions in viability in MIA PaCa-2 cells (Figure 3B), whereas the effects in PANC-1 and BxPC-3 cells were more dependent on the concentrations of both MR and DOX. MY produced comparatively modest changes, with significant reductions relative to the corresponding DOX-treated groups observed mainly at higher maca concentrations (Figure 3A). MB showed the most pronounced reductions in viability in PANC-1 cells (Figure 3C), whereas its effects were more limited in MIA PaCa-2 (Figure 3B) and BxPC-3 cells (Figure 3A).

Overall, combined DOX–maca treatment produced preparation-, cell line-, and concentration-dependent changes in viability. Because these changes alone do not establish a favorable drug interaction, the combinations were subsequently evaluated using Bliss independence and Highest Single Agent (HSA) models.

### 2.4. Interaction Analysis Reveals Context-Dependent Effects of Maca Preparations on DOX Response

**Figure 4:**
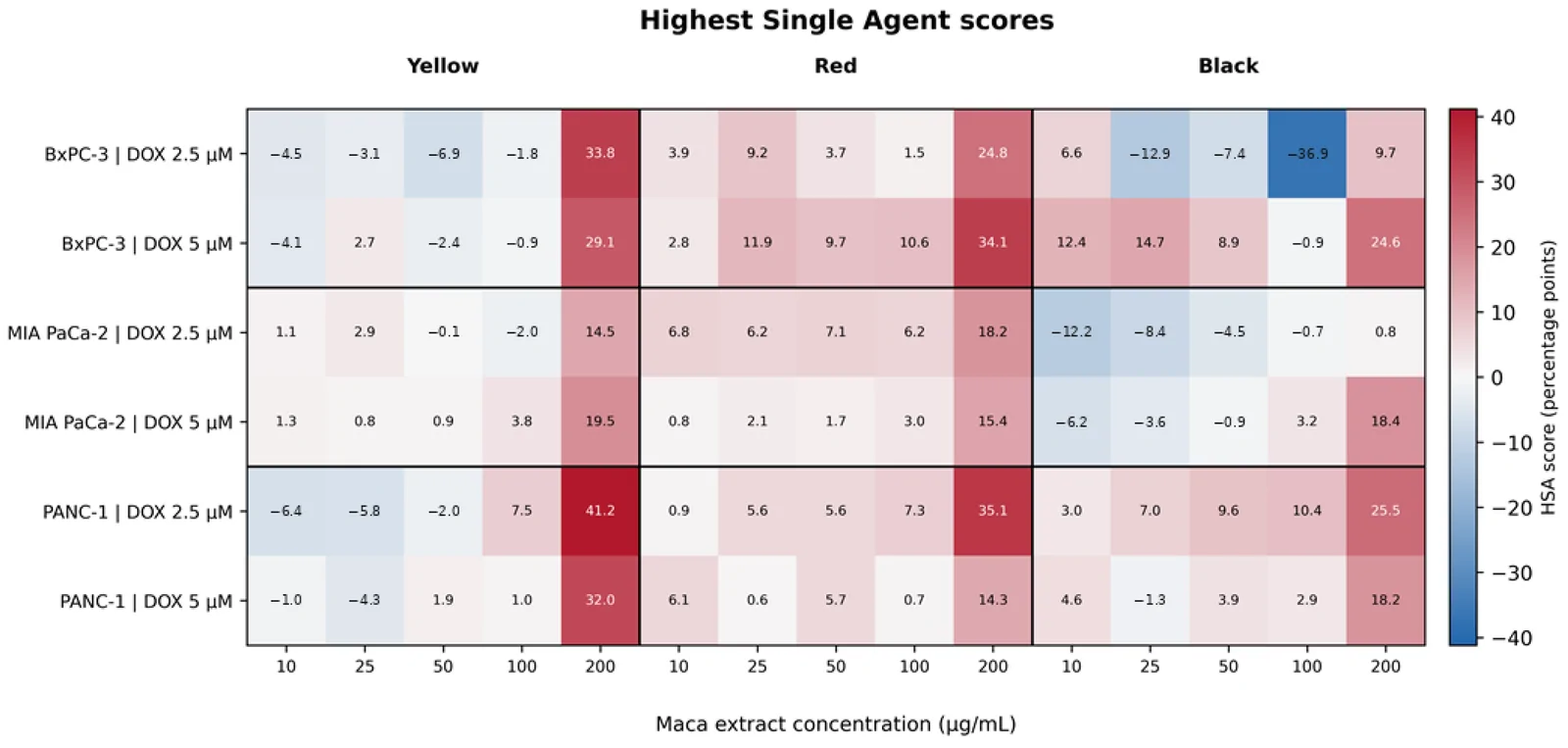
Heatmap of ΔBliss interaction scores for combinations of DOX and the tested maca preparations in BxPC-3, MIA PaCa-2, and PANC-1 cells after 24 h treatment. Columns represent MY, MR, and MB at concentrations of 10, 25, 50, 100, and 200 µg/mL. Rows represent cells treated with DOX at 2.5 or 5 µM. Positive ΔBliss values indicate greater-than-expected inhibition of cell viability according to the Bliss independence model, whereas negative values indicate less-than-expected activity.

**Figure 5:**
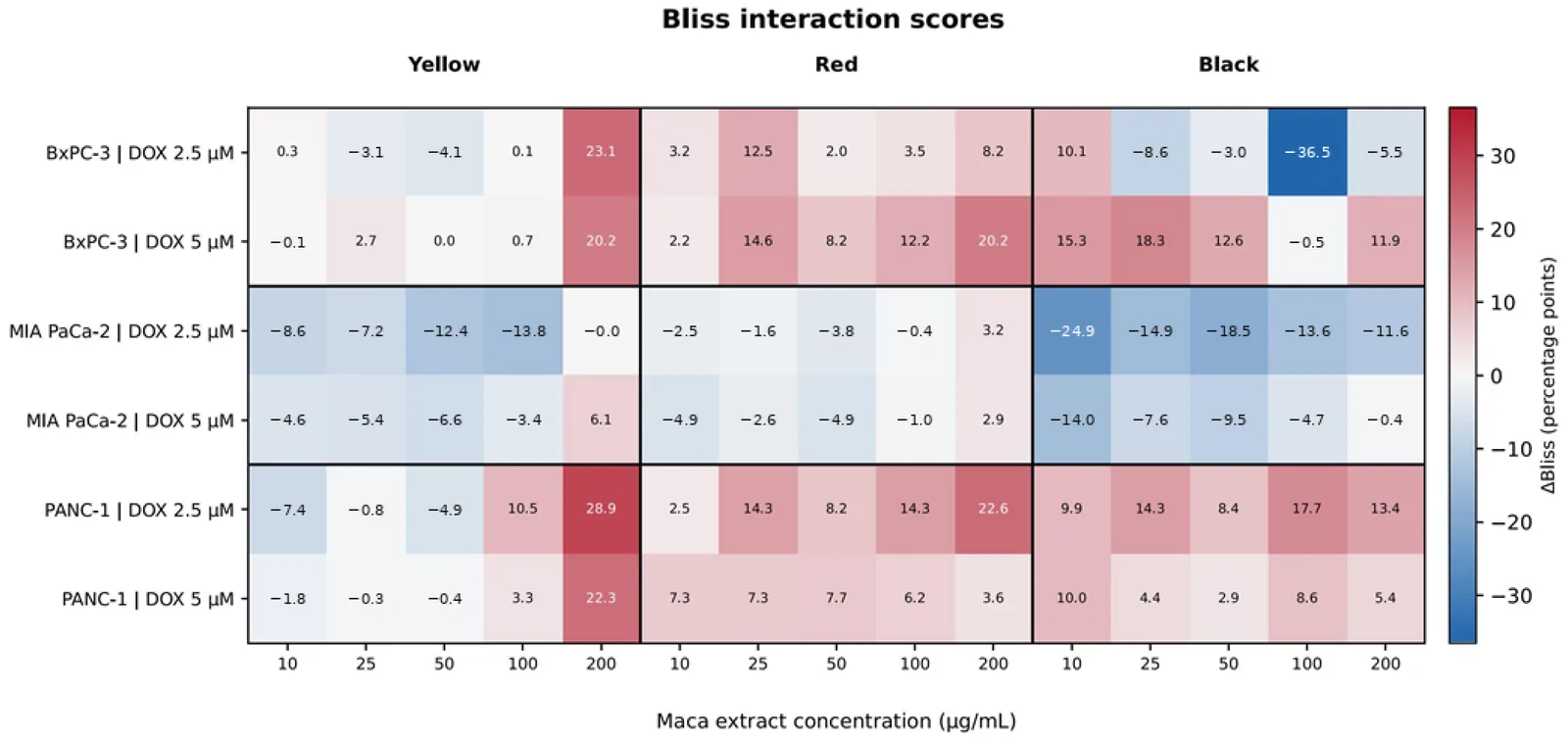
Heatmap of Highest Single Agent (HSA) interaction scores for combinations of DOX and the tested maca preparations in BxPC-3, MIA PaCa-2, and PANC-1 cells after 24 h treatment. Positive HSA values indicate that the combination reduced cell viability more effectively than the most active single-agent treatment.

The interaction between maca preparations and DOX varied according to pancreatic cancer cell line, maca preparation, and DOX concentration, as demonstrated by the Bliss independence and HSA analyses (Figure 4 and Figure 5). The most consistent favorable interactions were observed in PANC-1 cells treated with MR or MB in combination with 2.5 µM DOX, where positive ΔBliss and HSA scores indicated a greater inhibition of cell viability than predicted by the Bliss independence model or achieved by the most effective single-agent treatment. In BxPC-3 cells, favorable interactions were observed primarily at 5 µM DOX, particularly in combination with MR and MB (Figure 4 and Figure 5). In contrast, MIA PaCa-2 cells predominantly showed neutral or negative ΔBliss values, corresponding to approximately additive or less-than-additive interactions. This pattern was generally consistent with the corresponding HSA scores.

### 2.5. Maca Preparations Differentially Modify DOX-Associated Wound Closure

**Figure 6:**
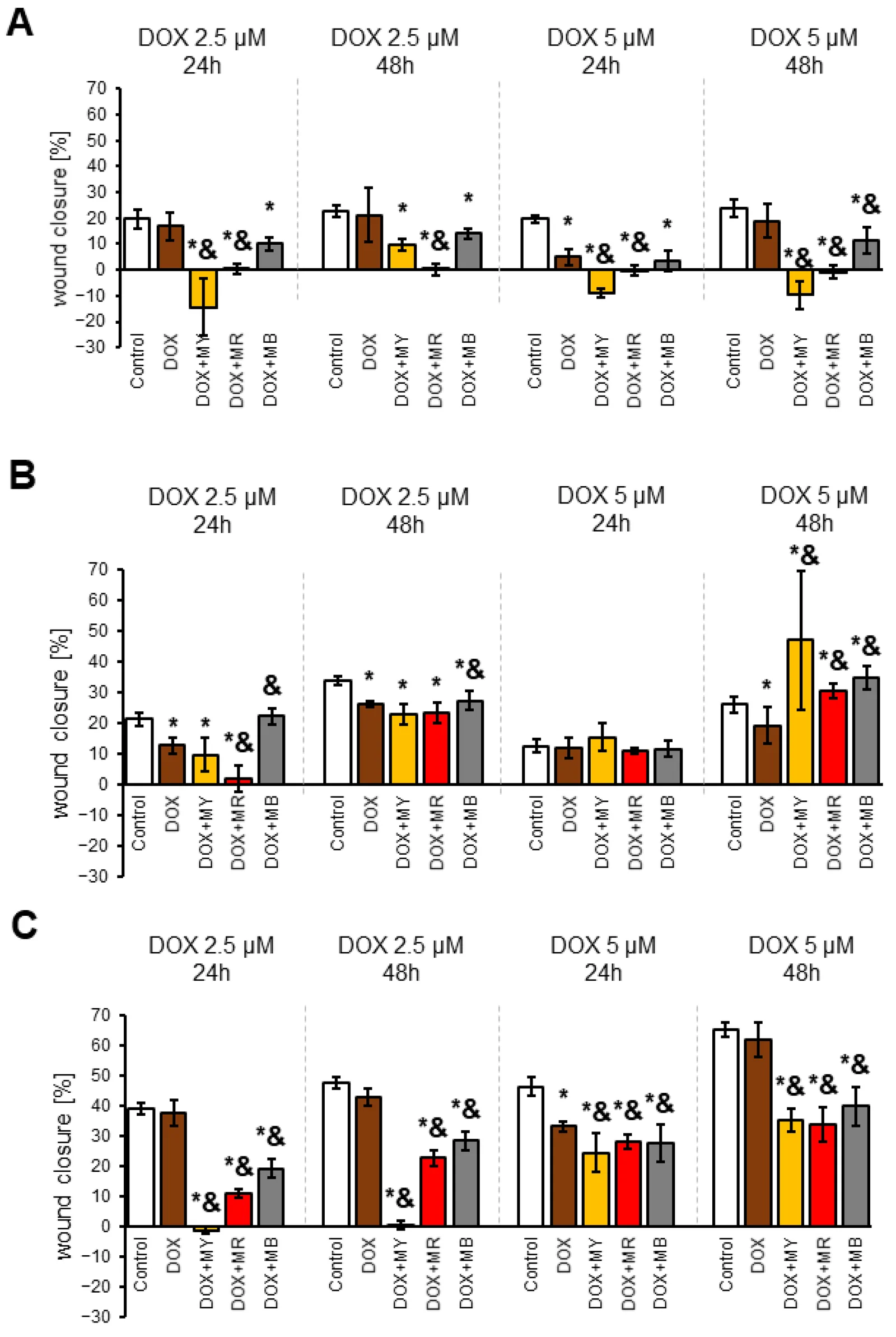
Percentage wound closure following DOX–maca co-treatment assessed by the wound healing assay. BxPC-3 (**A**), MIA PaCa-2 (**B**), and PANC-1 (**C**) cells were treated with DOX (2.5 or 5 µM) for 24 or 48 h, alone or in combination with MY, MR, or MB (100 µg/mL). Cells cultured in medium containing 1% FBS without DOX or maca preparations served as the control. Wound closure was calculated as the percentage reduction in wound width relative to the initial wound width (0 h), without additional normalization to the untreated control or DOX-treated groups. Measurements were performed under reduced-serum conditions to minimize serum-dependent effects on the assay. Representative microscopy images are provided in Supplementary Figures S2–S4. Data are presented as mean ± SD from three independent experiments (*n* = 3). * *p* < 0.05 vs. Control; & *p* < 0.05 vs. DOX.

To further evaluate the functional response to combined treatment, wound closure was assessed as the percentage reduction in wound width relative to the initial width (0 h) following co-treatment with DOX (2.5 or 5 µM) and maca preparations (100 µg/mL) (Figure 6A–C). Representative microscopy images of the wound healing assay are provided in Supplementary Figures S2–S4. In the BxPC-3 cells, co-treatment with MY or MR significantly reduced wound closure compared with DOX alone at both 24 and 48 h, whereas MB produced a significant reduction only after 48 h in combination with 5 µM DOX (Figure 6A).

In MIA PaCa-2 cells, MR further reduced wound closure compared with 2.5 µM DOX alone after 24 h, whereas MB increased wound closure relative to DOX alone at both time points. In the presence of 5 µM DOX, significant differences were observed only after 48 h, with MY producing the greatest increase in wound closure (Figure 6B).

In PANC-1 cells, wound closure was reduced under all co-treatment conditions compared with the corresponding DOX-treated groups, with MY producing the most pronounced reduction irrespective of DOX concentration or treatment duration (Figure 6C).

### 2.6. Maca Preparations Differentially Modify DOX-Induced Caspase-3 Activation

**Figure 7:**
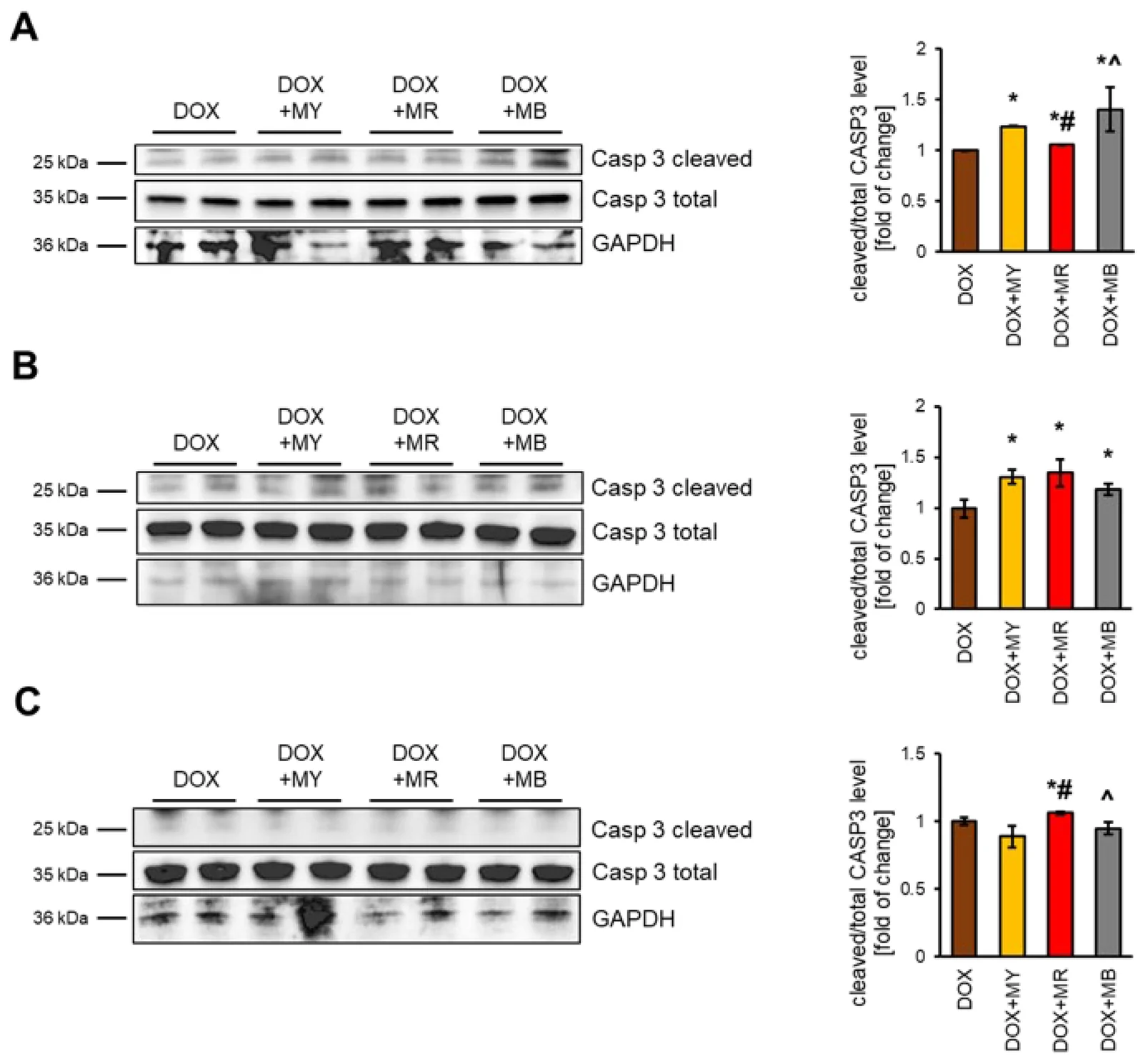
CASP3 activation following DOX–maca co-treatment in pancreatic cancer cells. BxPC-3 (**A**), MIA PaCa-2 (**B**), and PANC-1 (**C**) cells were treated with DOX (2.5 µM), alone or in combination with MY, MR, or MB (100 µg/mL) for 24 h. Representative immunoblots and corresponding densitometric analyses of cleaved CASP3 relative to total CASP3 are shown. CASP3 band intensities were normalized to total protein using Stain-Free imaging of the corresponding PVDF membrane lanes before calculating the cleaved-to-total CASP3 ratio. GAPDH immunoblots are presented as supplementary reference images and were not used for densitometric normalization or calculation of the cleaved-to-total CASP3 ratio. Data are presented as mean ± SD from three independent experiments (*n* = 3). * *p* < 0.05 vs. DOX; # *p* < 0.05 vs. DOX + MY; ^ *p* < 0.05 vs. DOX + MR.

To examine whether the effects of combined treatment were associated with changes in apoptosis-related signaling, CASP3 activation was evaluated after 24 h co-treatment with DOX (2.5 µM) and maca preparations (100 µg/mL). Co-treatment with MY, MR, or MB significantly increased CASP3 activation compared with DOX alone in both BxPC-3 (Figure 7A) and MIA PaCa-2 (Figure 7B) cells. In PANC-1 cells, a significant increase in CASP3 activation was observed only following co-treatment with MR (Figure 7C). Overall, these findings indicate that maca preparations differentially modified DOX-associated CASP3 activation in a cell line- and preparation-dependent manner.

### 2.7. Maca Preparations Differentially Modulate Survival-Related Protein Levels During DOX Treatment

**Figure 8:**
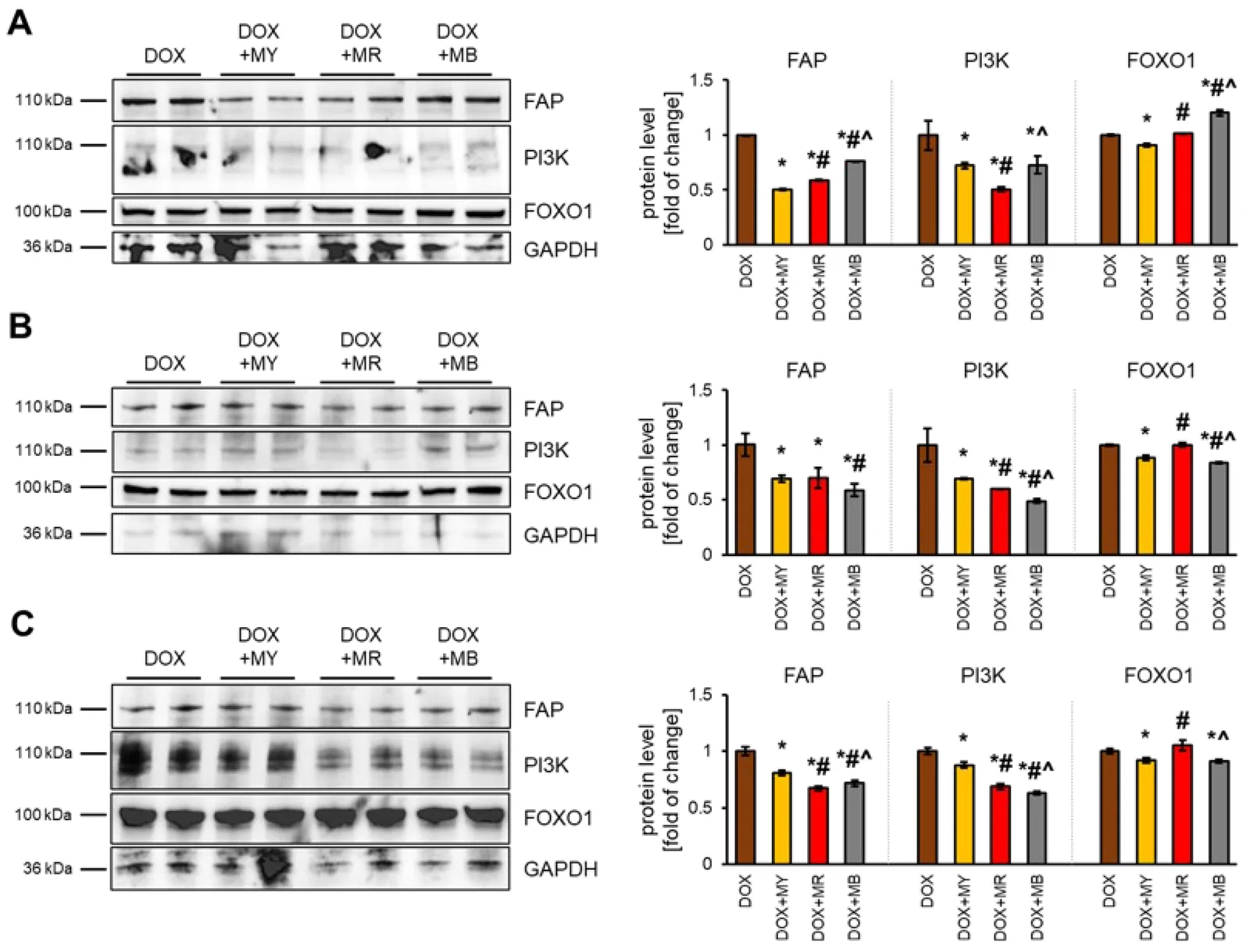
Protein levels of FAP, PI3K, and FOXO1 following DOX–maca co-treatment in pancreatic cancer cells. BxPC-3 (**A**), MIA PaCa-2 (**B**), and PANC-1 (**C**) cells were treated with DOX (2.5 µM), alone or in combination with the maca preparations (100 µg/mL) for 24 h. Representative immunoblots and corresponding densitometric analyses normalized to total protein using Stain-Free imaging of the corresponding PVDF membranes are shown. GAPDH immunoblots are presented as supplementary reference images and were not used for densitometric normalization. In PANC-1 cells panel (**C**), GAPDH and PI3K were detected on the same PVDF membrane. Data are presented as mean ± SD from three independent experiments (*n* = 3). * *p* < 0.05 vs. DOX; # *p* < 0.05 vs. DOX + MY; ^ *p* < 0.05 vs. DOX + MR.

To determine whether the observed treatment responses were accompanied by changes in survival-related signaling, FAP, PI3K, and FOXO1 protein levels were evaluated following 24 h co-treatment with DOX (2.5 µM) and maca preparations (100 µg/mL). Co-treatment with MY, MR, or MB reduced both the FAP and PI3K protein levels in the BxPC-3, MIA PaCa-2, and PANC-1 cells compared with DOX treatment alone (Figure 8A–C). In contrast, FOXO1 protein levels showed a more heterogeneous response. MY decreased FOXO1 levels in all three cell lines, whereas MR did not significantly alter the FOXO1 levels compared with DOX alone. MB increased the FOXO1 levels in BxPC-3 cells but decreased them in the MIA PaCa-2 and PANC-1 cells (Figure 8A–C).

### 2.8. Maca Preparations Differentially Modify Lipid Droplet Accumulation During DOX Treatment

LD accumulation was evaluated following co-treatment with DOX (2.5 or 5 µM) and maca preparations (100 µg/mL). Representative Oil Red O-stained images are shown in Supplementary Figures S5–S7.

**Figure 9:**
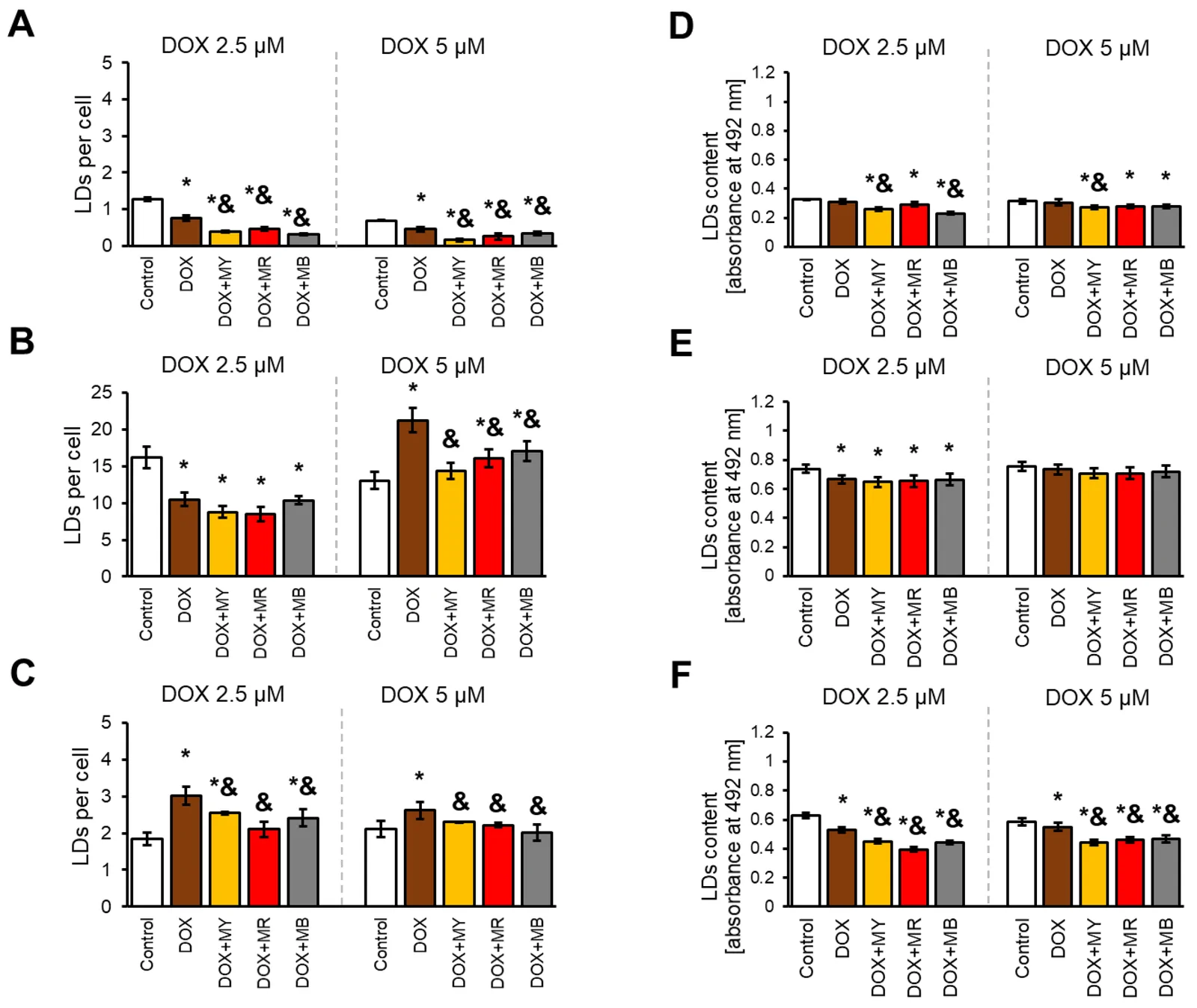
Lipid droplet (LD) accumulation following DOX–maca co-treatment in pancreatic cancer cells. Cells were treated with DOX (2.5 or 5 µM), alone or in combination with maca preparations (100 µg/mL). (**A**–**C**) Quantification of LDs per cell in BxPC-3 (**A**), MIA PaCa-2 (**B**), and PANC-1 (**C**) cells following Oil Red O staining. (**D**–**F**) Spectrophotometric quantification of extracted Oil Red O dye based on absorbance measured at 492 nm in BxPC-3 (**D**), MIA PaCa-2 (**E**), and PANC-1 (**F**) cells. Control cells were cultured in medium containing 1% FBS without DOX or maca preparations. Data are presented as mean ± SD from three independent experiments (*n* = 3). * *p* < 0.05 vs. Control; & *p* < 0.05 vs. DOX.

Quantitative image analysis showed reduced LD accumulation in BxPC-3 cells at both DOX concentrations (Figure 9A), in MIA PaCa-2 cells following treatment with 5 µM DOX (Figure 9B), and in PANC-1 cells at both DOX concentrations (Figure 9C), relative to the corresponding DOX-treated groups.

Spectrophotometric quantification of extracted Oil Red O dye further demonstrated treatment-dependent differences in intracellular lipid content (Figure 9D–F). In BxPC-3 cells, co-treatment with MY or MB significantly reduced the lipid content at 2.5 µM DOX, whereas at 5 µM DOX, a significant reduction was observed only with MY (Figure 9D). In MIA PaCa-2 cells, no significant changes in total intracellular lipid content were detected under the tested co-treatment conditions (Figure 9E). In contrast, PANC-1 cells showed a consistent reduction in intracellular lipid content following co-treatment with all three maca preparations at both DOX concentrations (Figure 9F).

Overall, the effects of maca preparations on LD accumulation and intracellular lipid content were cell line-dependent, with the most consistent reductions observed in PANC-1 cells.

### 2.9. Maca Preparations Differentially Modify Lipid Metabolism-Related Protein Levels During DOX Treatment

**Figure 10:**
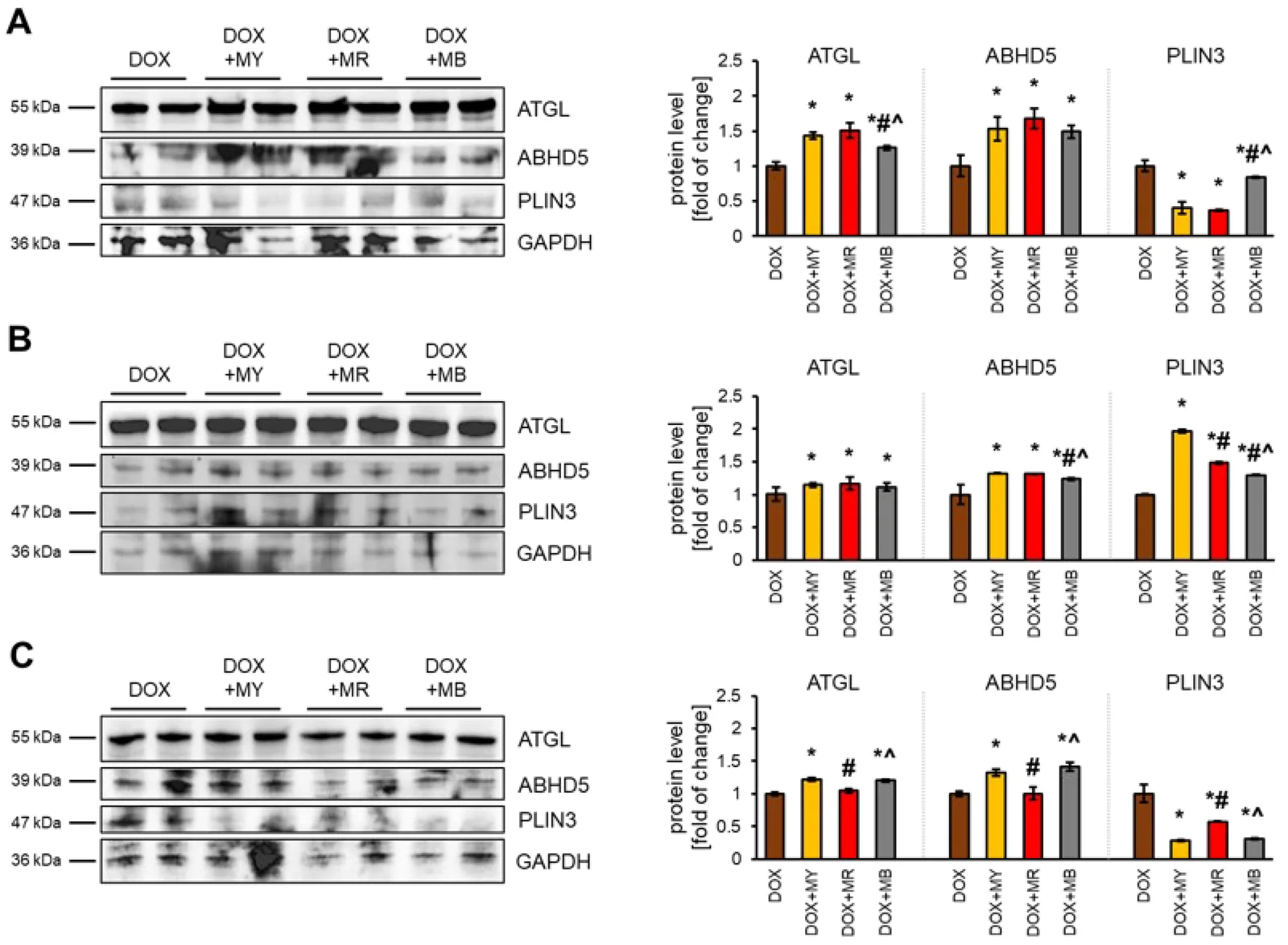
Protein levels of the lipolytic regulators ATGL, ABHD5, and PLIN3 following DOX–maca co-treatment in pancreatic cancer cells. BxPC-3 (**A**), MIA PaCa-2 (**B**), and PANC-1 (**C**) cells were treated with DOX (2.5 µM), alone or in combination with maca preparations (100 µg/mL) for 24 h. Representative immunoblots and corresponding densitometric analyses normalized to total protein using Stain-Free imaging of the corresponding PVDF membranes are shown. GAPDH immunoblots are presented as supplementary reference images and were not used for densitometric normalization. In PANC-1 cells panel (**C**), GAPDH and ATGL were detected on the same PVDF membrane. Data are presented as mean ± SD from three independent experiments (*n* = 3). * *p* < 0.05 vs. DOX; # *p* < 0.05 vs. DOX + MY; ^ *p* < 0.05 vs. DOX + MR.

ATGL, ABHD5, and PLIN3 protein levels were evaluated following co-treatment with DOX and the maca preparations. In the BxPC-3 (Figure 10A) and MIA PaCa-2 (Figure 10B) cells, co-treatment with MY, MR, or MB increased the ATGL and ABHD5 levels compared with DOX alone. In the PANC-1 cells, increased ATGL and ABHD5 levels were observed following co-treatment with MY and MB, whereas MR did not significantly alter either protein (Figure 10C). PLIN3 showed a distinct cell line-dependent response, with reduced levels in BxPC-3 and PANC-1 cells but increased levels in the MIA PaCa-2 cells (Figure 10A–C).

Overall, changes in the ATGL, ABHD5, and PLIN3 protein levels were cell line- and morphotype-dependent and occurred in parallel with differential changes in cellular lipid accumulation during DOX–maca co-treatment.

## 3. Discussion

### 3.1. Maca Preparations and Experimental Quality Assessment

Quality assessment of botanical preparations is an important element in studies evaluating their biological activity. In the present study, radiological characterization was included as an additional quality-control measure before biological evaluation. *Lepidium meyenii* is traditionally cultivated at high elevations in the central Andes, frequently exceeding 3500–4500 m above sea level [17,18], where naturally occurring radionuclides may be transferred from soil to plant material [28,29]. HPGe gamma-ray spectrometry detected only naturally occurring ^40^K, whereas no anthropogenic radionuclides were identified in the tested preparations, supporting their suitability for subsequent biological experiments.

The phytochemical composition of *L. meyenii* has been extensively investigated and includes several major classes of bioactive constituents, particularly glucosinolates, macamides, macaenes, alkaloids, phytosterols, and polyphenolic compounds [17,18,22,23,30]. The phytochemical composition of maca varies with morphotype, geographical origin, cultivation conditions, and processing [17,18,30,31]. Recent studies have reported differences in major secondary metabolites, including macamides, macaenes, glucosinolates, and polyphenols, between maca materials of different origins and morphotypes [17,18,31,32]. Representative data relevant to the interpretation of the present findings are summarized in Table 2.

The available phytochemical data demonstrate considerable variation in maca composition depending on geographical origin, hypocotyl morphotype, and processing conditions (Table 2). Although all three preparations investigated in the present study originated from Peru, previous studies have reported phytochemical differences among Peruvian maca morphotypes [17,18,22,23,30,31,32,33,34,35,36,37,38,39,40]. These findings provide a relevant chemical context for the distinct biological responses observed among MY, MR, and MB. Nevertheless, as the tested batches were not subjected to HPLC or LC-MS profiling, the observed differences cannot be attributed specifically to morphotype or individual phytochemical constituents. Future batch-specific characterization will be necessary to clarify the chemical basis of these responses.

### 3.2. Cell Line-Dependent Responses to DOX–Maca Co-Treatment

The present study showed that the effects of the tested MY, MR, and MB preparations on DOX response varied markedly among pancreatic cancer cell lines, consistent with the molecular heterogeneity of this disease [5,6,7,8]. Differences among BxPC-3, MIA PaCa-2, and PANC-1 cells may reflect variation in oncogenic signaling, stress-response pathways, and metabolic adaptation, which can influence treatment sensitivity.

Although co-treatment frequently reduced cell viability relative to DOX alone, these changes did not uniformly translate into favorable drug interactions. Bliss independence and HSA analyses revealed the most consistent favorable interaction profiles in PANC-1 cells treated with MR or MB in combination with 2.5 µM DOX. BxPC-3 cells showed favorable interactions mainly at 5 µM DOX, whereas MIA PaCa-2 cells predominantly exhibited neutral or less-than-additive interactions despite showing pronounced reductions in viability. These findings emphasize that the magnitude of viability loss alone is insufficient to establish a favorable interaction between maca preparations and DOX.

The context-dependent nature of maca activity is consistent with previous observations showing that biological responses to maca preparations may vary according to cell type and experimental conditions [41]. Thus, the observed responses may reflect differences among the tested maca preparations, DOX concentrations, and the intrinsic biological characteristics of individual pancreatic cancer cell lines.

The interplay between metabolic reprogramming and cell-death pathways is increasingly recognized as an important component of cancer treatment responses [42]. DOX is not a standard first-line treatment for pancreatic cancer but was used in the present study as a well-characterized inducer of genotoxic and cellular stress [9,43,44]. Accordingly, the experimental model should be viewed primarily as a system for investigating how maca preparations modify pancreatic cancer cell responses to anticancer drug-induced stress rather than as a direct representation of current clinical treatment regimens. The interaction patterns observed with DOX cannot be directly extrapolated to clinically relevant pancreatic cancer treatments, such as gemcitabine- or taxane-based regimens. Gemcitabine interferes primarily with DNA synthesis, whereas taxanes target microtubule dynamics, and these mechanistic differences may result in distinct cellular and metabolic responses [45,46]. This may be particularly relevant in pancreatic cancer, where metabolic reprogramming, including lipid metabolism, has been associated with chemotherapeutic response and resistance [47]. Therefore, the Bliss/HSA interaction profiles identified here should be considered specific to the DOX-based model until validated using clinically relevant pancreatic cancer therapies.

The most favorable interaction profiles coincided with pronounced changes in lipid-related parameters, particularly in the PANC-1 cells. However, the present data do not establish a causal relationship between these metabolic changes and the observed interaction with DOX. Rather, they support further investigation of whether alterations in lipid homeostasis contribute to the differential responses of pancreatic cancer cells to combined treatment.

### 3.3. Changes in Survival-Related Proteins During DOX–Maca Co-Treatment

DOX–maca co-treatment was associated with changes in the levels of proteins involved in survival- and stress-related signaling, including PI3K, FAP, and FOXO1. The PI3K/AKT pathway is an important regulator of tumor cell survival, growth, and metabolic adaptation [48,49], whereas FOXO1 functions as a stress-responsive transcription factor involved in the regulation of apoptosis and metabolic homeostasis [12]. In the present study, all three maca preparations reduced PI3K and FAP protein levels relative to DOX alone across the three pancreatic cancer cell lines. These changes indicate that survival-related signaling components are responsive to combined treatment, although pathway activity was not directly assessed.

FAP has been implicated in tumor progression and signaling in the pancreatic cancer microenvironment [13,50]. Although FAP is predominantly associated with cancer-associated fibroblasts, its expression has also been reported in tumor cells, including pancreatic cancer cells [13,50], supporting its evaluation in the present monoculture model. The consistent reduction in FAP protein levels following DOX–maca co-treatment therefore represents an additional molecular response associated with combined treatment. Because FAP has been linked to signaling pathways involved in tumor progression [13,50], the parallel reduction in FAP and PI3K observed here warrants further investigation; however, the present data do not establish a causal relationship between these changes.

In contrast to FAP and PI3K, FOXO1 showed a strongly cell line- and preparation-dependent response. MY reduced the FOXO1 protein levels in all three cell lines, MR produced no significant change relative to DOX alone, and MB increased FOXO1 in BxPC-3 cells while decreasing it in MIA PaCa-2 and PANC-1 cells. Given the role of FOXO1 in cellular responses to metabolic and oxidative stress [12], these differences are consistent with treatment-associated changes in stress-responsive signaling. However, because FOXO1 activity depends not only on the total protein abundance but also on post-translational regulation and subcellular localization, the functional consequences of these changes require further investigation.

The relationship between these molecular responses and DOX interaction profiles was not uniform across the three cell lines. Therefore, changes in FAP, PI3K, and FOXO1 should be interpreted as treatment-associated molecular responses rather than as established mechanisms underlying favorable DOX–maca interactions. Further studies assessing pathway activation and functional perturbation of these proteins will be required to determine their contribution to the observed cellular responses.

### 3.4. Lipid Accumulation and Lipid Metabolism-Related Responses

Alterations in lipid metabolism are increasingly recognized as an important component of pancreatic cancer adaptation to cellular stress [51,52]. Lipid metabolism is closely interconnected with oncogenic signaling pathways, including PI3K/AKT signaling [48]. LDs serve as dynamic reservoirs of neutral lipids and contribute to energy homeostasis, membrane synthesis, and protection against metabolic and oxidative stress [14,52,53]. Recent studies in pancreatic cancer have further demonstrated that LD formation and triglyceride storage contribute to metabolic adaptation under stress and can influence the response to chemotherapy, including gemcitabine- and FOLFIRINOX-based treatments [54,55]. In the present study, DOX–maca co-treatment was associated with changes in LD accumulation and intracellular lipid content, with the most consistent reductions observed in PANC-1 cells.

Image-based and spectrophotometric analyses did not produce identical patterns across all cell lines. In particular, MIA PaCa-2 cells showed reduced LD accumulation by image analysis following treatment with 5 µM DOX combined with maca preparations, whereas spectrophotometric Oil Red O quantification did not reveal significant changes in total intracellular lipid content. This difference may reflect the distinct parameters captured by the two approaches: image analysis evaluates LD abundance at the cellular level, whereas spectrophotometric measurements reflect total Oil Red O-associated lipid content in the analyzed cell population. Thus, these methods should be regarded as complementary rather than directly interchangeable.

Changes in lipid accumulation were accompanied by alterations in ATGL, ABHD5, and PLIN3 protein levels. ATGL and its co-activator ABHD5 are involved in triglyceride mobilization from LDs, whereas PLIN3 contributes to LD organization and the regulation of lipid storage and mobilization [15,56,57,58]. Increased ATGL and ABHD5 levels were observed following co-treatment with all three maca preparations in BxPC-3 and MIA PaCa-2 cells and with MY and MB in PANC-1 cells. These changes are compatible with altered lipid mobilization; however, because lipolytic flux was not directly measured, they cannot be interpreted as evidence of enhanced lipolysis. Recent functional evidence from ovarian cancer models has linked ATGL-mediated triglyceride hydrolysis to increased resistance to cisplatin, highlighting the potential relevance of lipid mobilization to chemotherapy responses [59]. However, whether similar mechanisms operate in pancreatic cancer cells remains to be established.

PLIN3 showed a distinct cell line-dependent response, with reduced protein levels in BxPC-3 and PANC-1 cells but increased levels in MIA PaCa-2 cells. Together with the differences in LD accumulation and ATGL/ABHD5 levels, this finding further indicates that the lipid-related response to DOX–maca co-treatment differed among the pancreatic cancer cell lines. Previous studies have implicated ATGL and ABHD5 in the regulation of tumor lipid metabolism and cellular metabolic adaptation [55,56], while morphotype-specific maca preparations have also been reported to affect cellular metabolism in non-cancerous experimental systems [60].

Changes in lipid-related parameters were accompanied by increased CASP3 activation under several co-treatment conditions. However, the relationship between these responses was not uniform across the three cell lines. For example, MIA PaCa-2 cells showed increased CASP3 activation following co-treatment with all three maca preparations despite the absence of significant changes in total intracellular lipid content. Therefore, the present findings do not establish lipid depletion or increased lipid mobilization as a direct mechanism of apoptosis. Instead, they indicate that apoptosis-associated and lipid-related responses occur in parallel and vary according to cellular context.

Overall, the combined changes in survival-related proteins, lipid accumulation, lipid metabolism-related proteins, and CASP3 activation indicate that DOX–maca co-treatment is associated with multiple, cell line-dependent molecular and metabolic responses. The present data do not establish a causal sequence linking these processes, and further studies incorporating direct measurements of lipid flux, pathway activity, and functional perturbation of individual molecular targets will be required to determine their mechanistic relationships.

**Figure 11:**
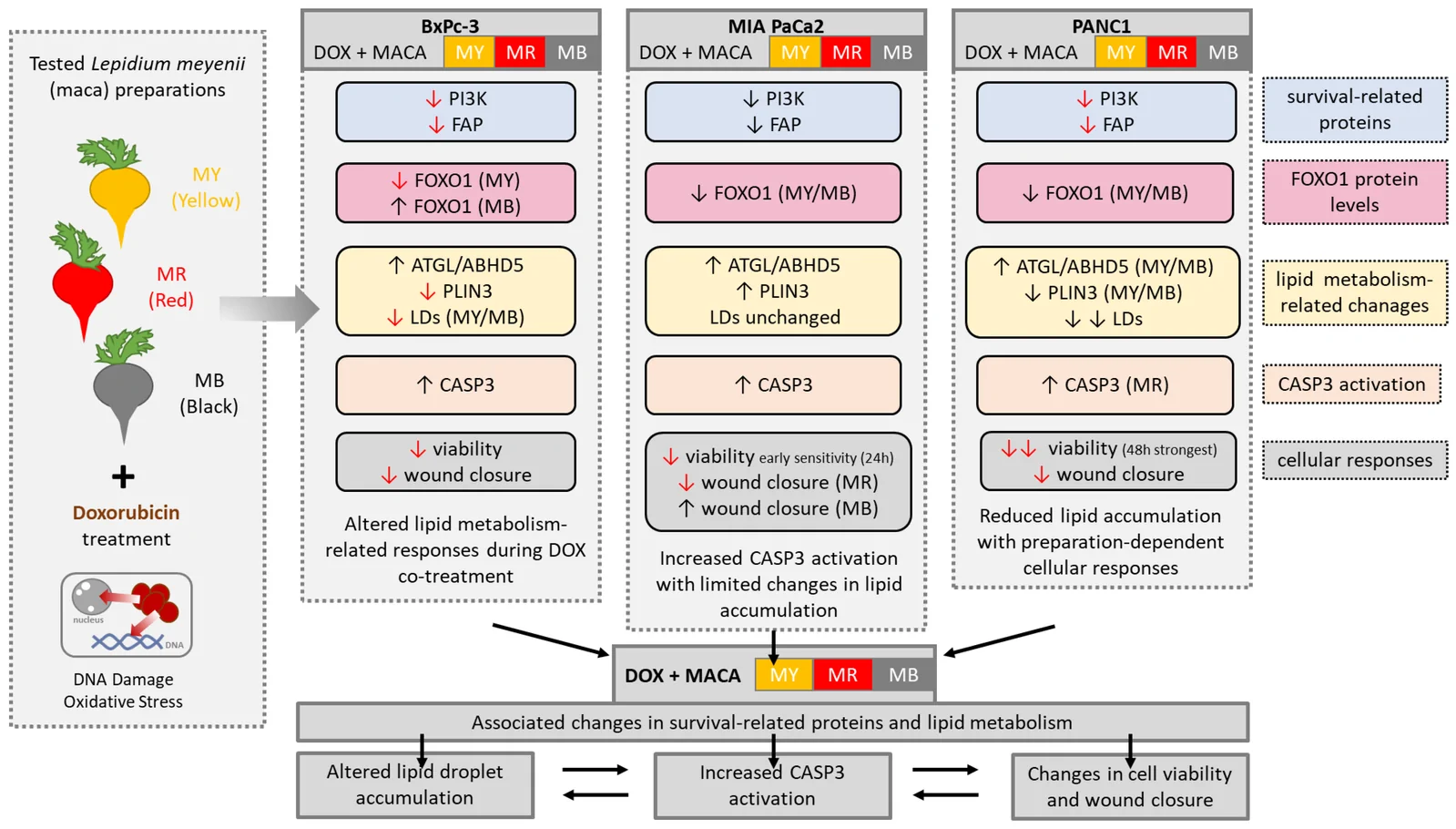
Summary of cell line-dependent molecular and metabolic responses to DOX–maca co-treatment. Schematic representation of the treatment-associated molecular and metabolic responses observed following combined doxorubicin (DOX) and the tested maca preparations (Maca Yellow, MY; Maca Red, MR; Maca Black, MB) in BxPC-3, MIA PaCa-2, and PANC-1 pancreatic cancer cells. Across all three cell lines, DOX–maca co-treatment reduced protein levels of phosphoinositide 3-kinase (PI3K) and fibroblast activation protein (FAP), whereas forkhead box O1 (FOXO1) showed cell line- and preparation-dependent modulation. Co-treatment was also associated with differential changes in lipid droplet (LD) accumulation and in the levels of the lipid metabolism-related proteins adipose triglyceride lipase (ATGL), α/β-hydrolase domain-containing protein 5 (ABHD5), and perilipin 3 (PLIN3). These molecular and metabolic responses occurred in parallel with cell line- and preparation-dependent changes in caspase-3 (CASP3) activation, wound closure, and cell viability. The most consistent favorable interactions identified by Bliss independence and Highest Single Agent (HSA) analyses were observed in PANC-1 cells treated with MR or MB in combination with 2.5 µM DOX, whereas BxPC-3 cells showed favorable interactions primarily at 5 µM DOX and MIA PaCa-2 cells predominantly exhibited neutral or less-than-additive interactions. The schematic summarizes treatment-associated relationships and does not imply a causal sequence between survival signaling, lipid remodeling, and cellular response to DOX.

Taken together, the present findings indicate that DOX–maca co-treatment is associated with alterations in survival-related signaling and lipid metabolic homeostasis that vary according to the tested preparation and cell line. The most consistent favorable interaction profiles identified by Bliss and HSA analyses were observed in PANC-1 cells treated with MR or MB in combination with 2.5 µM DOX, a cellular context that also showed pronounced alterations in lipid-related parameters. However, because these associations were not uniform across all cell lines and neither lipid flux nor pathway activity was directly assessed, the present data do not establish metabolic remodeling as a causal determinant of DOX sensitivity. Instead, they identify lipid metabolic plasticity as a candidate process warranting further mechanistic investigation. A schematic overview integrating these cell line-dependent treatment-associated responses is presented in Figure 11.

From a natural-product perspective, the differences observed among the tested MY, MR, and MB preparations indicate that these specific commercial products should not be assumed to be pharmacologically interchangeable. Whether these differences are consistently associated with maca morphotypes requires further investigation using chemically characterized preparations. At the same time, the divergent responses of BxPC-3, MIA PaCa-2, and PANC-1 cells demonstrate that the biological effects of these preparations are strongly influenced by tumor-cell context. These two levels of heterogeneity, botanical and cellular, represent important considerations for the preclinical evaluation of complex natural products as potential modulators of anticancer drug responses. Establishing whether the observed effects are reproducible and therapeutically relevant will require the integration of phytochemical characterization and standardization with functional mechanistic studies, clinically relevant anticancer regimens, and advanced preclinical models.

### 3.5. Study Limitations

Several limitations should be considered when interpreting the present findings. First, commercially available morphotype-specific maca products were evaluated as complete botanical preparations, and no direct HPLC- or LC-MS-based phytochemical characterization of the tested products was performed. Consequently, the observed biological effects should be interpreted as properties of the specific MY, MR, and MB preparations evaluated in this study and cannot be attributed to individual phytochemical constituents. Likewise, differences among the three preparations cannot be ascribed to differences in specific macamides, glucosinolates, polyphenols, sterols, or other compounds on the basis of the present data. Future studies should incorporate chromatographic and mass-spectrometric profiling to characterize product composition, identify potential bioactive constituents, and improve the standardization and reproducibility of morphotype-specific maca preparations used in pharmacological studies. The recovery of maca-derived material after filtration was not quantitatively assessed; therefore, the reported concentrations should be considered nominal powder-equivalent concentrations.

Second, the present study identified coordinated changes in survival-related signaling, lipid metabolism-related parameters, and cellular responses to treatment; however, the underlying molecular relationships were not directly validated. In particular, changes in protein abundance do not necessarily reflect pathway activity or metabolic flux. Although treatment-associated changes in lipid droplet accumulation and the protein levels of ATGL, ABHD5, PLIN3, PI3K, FAP, and FOXO1 were observed, their causal involvement in the cellular response to treatment remains unconfirmed. Functional studies involving targeted inhibition or gene silencing are required to establish the mechanistic significance of these findings. Further studies employing pathway-specific inhibitors, gene silencing or other functional perturbation approaches, together with direct measurements of lipid flux, will be required to determine whether the observed molecular and metabolic alterations contribute causally to the differential responses to DOX–maca co-treatment.

Third, apoptosis-associated protein analyses were performed in DOX-treated groups and did not include corresponding maca-only groups. The primary objective of these experiments was to investigate whether maca preparations modify apoptosis-associated responses to DOX rather than to characterize their independent pro-apoptotic activity. This approach was supported by the viability experiments, in which maca preparations generally exerted limited effects at the concentration selected for molecular analyses (100 µg/mL). Nevertheless, the absence of maca-only groups prevents us from distinguishing the direct effects of maca preparations on CASP3 activation from effects specifically associated with DOX co-treatment.

An additional limitation is the use of DOX as an experimental model of anticancer drug-induced stress. DOX is not a standard first-line treatment for pancreatic cancer; therefore, the present findings should not be directly extrapolated to current clinical pancreatic cancer chemotherapy regimens. Future studies should determine whether comparable effects occur in combination with clinically relevant agents and treatment regimens.

## 4. Materials and Methods

### 4.1. Materials

Primary antibodies against FAP (EPR20021) and secondary goat anti-rabbit HRP antibody (ab97051) were obtained from Abcam (Cambridge, UK). GAPDH (4G5) and secondary peroxidase-conjugated rat anti-mouse HRP antibody (LO-ME-3) were purchased from Bio-Rad (Hercules, CA, USA). ATGL (#2138), Caspase-3 (#9662), and PI3K (#4255) antibodies were obtained from Cell Signaling Technology (Danvers, MA, USA). FOXO1 (ZRB1862-25UL) antibody was obtained from Merck (Darmstadt, Germany). ABHD5 (sc-100468) and PLIN3 (sc-390968) antibodies were purchased from Santa Cruz Biotechnology (Santa Cruz, CA, USA). Unless otherwise stated, all chemicals and reagents were purchased from Sigma-Aldrich (St. Louis, MO, USA).

### 4.2. Maca Products, Preparation, and Radiological Quality Assessment

Commercially available yellow (MY), red (MR), and black (MB) dried root powders of *Lepidium meyenii* Walp. (Brassicaceae), originating from Peru, were used in this study. The products were supplied in Poland by NANGA Przemysław Figura (Zlotow, Poland). According to the available product specifications, the powdered maca products were single-ingredient preparations consisting of 100% maca root, with no additional botanical ingredients or excipients declared. The products were marketed as the corresponding yellow, red, and black maca variants. The corresponding product/lot identifiers of the materials used in this study were MY 539, MR 010402240RG, and MB MNPO-241000279.

For experimental use, each finely milled maca root powder was suspended in dimethyl sulfoxide (DMSO; J66650.AE, Thermo Fisher Scientific, Grand Island, NY, USA) at an initial concentration of 10 mg/mL and subsequently filtered through a 0.45 µm filter. The preparations appeared visually homogeneous, with no visible insoluble residue. According to the manufacturers’ certificates of analysis, the powders met a particle size specification of 60 mesh. The concentrations reported in this study were calculated based on the initial mass of maca powder used for preparation.

As an additional quality-control procedure, radiological characterization of the maca preparations was performed before biological experiments using high-purity germanium (HPGe; ORTEC, AMETEK, Wokingham, UK) gamma-ray spectrometry. Gamma-ray spectra were analyzed using reference isotope libraries to identify radionuclides and calculate their activities from background-corrected peak areas using calibrated full-energy peak efficiencies.

The present study evaluated the biological activity of the complete morphotype-specific maca preparations rather than individual isolated constituents.

### 4.3. Cell Culture

Human pancreatic cancer cell lines BxPC-3 (CRL-1687, ATCC, Manassas, VA, USA), MIA PaCa-2 (CRL-1420, ATCC), and PANC-1 (CRL-1469-LUC2, ATCC) were used. PANC-1-Luc2 cells are referred to as PANC-1 throughout the manuscript for simplicity. BxPC-3 cells were cultured in RPMI-1640 medium (01885, ATCC) supplemented with 10% (*v*/*v*) FBS (F7524, Sigma-Aldrich, St. Louis, MO, USA), Antibiotic Antimycotic Solution 100× (with 10,000 units penicillin, 10 mg streptomycin, and 25 μg amphotericin B per mL, A5955, Merck, St. Louis, MO, USA). MIA PaCa-2 cells were maintained in DMEM (00691, ATCC) supplemented with 10% (*v*/*v*) FBS (F7524, Sigma), 2.5% horse serum (16050-122, Gibco, Thermo Fisher Scientific, Grand Island, NY, USA), and Antibiotic Antimycotic Solution 100× (A5955, Merck). PANC-1 cells were cultured in DMEM (00691, ATCC) supplemented with 10% (*v*/*v*) FBS (F7524, Sigma) and Antibiotic Antimycotic Solution 100× (A5955, Merck). Cells were incubated at 37 °C in a humidified atmosphere containing 5% CO_2_, and the medium was changed every 48 h.

For experiments, cells were cultured in medium containing 1% (*v*/*v*) FBS. Cells were treated with doxorubicin (DOX) hydrochloride (D1515, Sigma-Aldrich) at concentrations of 0.5, 1, 2.5, 5, 7.5, and 10 µM or MY, MR, and MB at concentrations of 10, 25, 50, 100, 200, and 500 µg/mL for 24 or 48 h. To evaluate the combined effects of maca preparations and DOX, cells were treated simultaneously with DOX at 2.5 or 5 µM and MY, MR, or MB preparations (10–500 µg/mL) for 24 h. Interaction analyses using the Bliss independence and HSA models were performed for maca concentrations of 10–200 µg/mL. These two common DOX concentrations were selected based on the dose-response analyses to enable direct comparison of combination effects across the three pancreatic cancer cell lines. Control cells were cultured in medium containing 1% FBS and an equivalent concentration of DMSO (at a concentration corresponding to the highest dose used in the experimental conditions). Although DOX is not a standard therapeutic agent for pancreatic cancer, it was used in the present study as a reproducible model of genotoxic, metabolic, and apoptotic stress.

Based on the viability data, 100 µg/mL was selected for subsequent molecular and functional analyses because this concentration produced limited effects on viability when applied alone while allowing an evaluation of treatment-associated changes during co-treatment.

### 4.4. Cell Viability Assay

Cells were seeded into 96-well plates at densities allowing approximately 70% confluence and treated with maca preparations or DOX for 24 or 48 h. Cell viability was assessed using the CellTiter-Blue reagent (G8080, Promega Corporation, Madison, WI, USA) according to the manufacturer’s instructions. Viable cells convert resazurin to resorufin, resulting in a colorimetric change measured using a Mobi µ2 MicroDigital plate reader (MicroDigital Co., Seoul, Republic of Korea) at 573 nm with a reference wavelength of 600 nm. Cell viability was expressed as a percentage relative to untreated control cells (100%).

Dose–response curves were fitted using nonlinear regression (variable slope). IC_25_ and IC_50_ values were calculated using a variable-slope dose-response model. For combination treatments, cell viability was determined using DOX at 2.5 or 5 µM together with different concentrations of MY, MR, or MB preparations for 24 h.

### 4.5. Drug Interaction Analysis

The interaction between DOX and morphotype-specific maca preparations was evaluated using the Bliss independence model and the Highest Single Agent (HSA) model. Cell viability values obtained for DOX alone, maca preparations alone, and combination treatments were normalized to untreated control cells.

For Bliss analysis, the expected viability was calculated assuming independent drug action and compared with the experimentally observed viability:

ΔBliss = expected viability − observed viability.

Positive ΔBliss values indicated greater-than-expected inhibition, values close to zero indicated approximately additive effects, and negative values indicated less-than-expected inhibition.

HSA scores were calculated by comparing the viability of each combination treatment with the most effective corresponding single-agent treatment. In addition, the percentage change in cell viability during DOX–maca co-treatment relative to DOX treatment alone was calculated as a descriptive measure and was not used to classify drug interactions.

Bliss and HSA analyses were performed independently for each biological experiment using GraphPad Prism (version 11.02; GraphPad Software, La Jolla, CA, USA). The resulting scores are presented as mean ± SD from three independent biological experiments.

### 4.6. Wound Healing Assays

BxPC-3, MIA PaCa-2, and PANC-1 cells were seeded in 6-well plates and grown to approximately 90% confluence. A linear scratch was created using a sterile 200-µL pipette tip, and detached cells were removed by washing with PBS. Fresh medium containing either the vehicle control or test compounds (DOX alone or in combination with MY, MR, or MB) was added. Cells were maintained in medium containing 1% FBS during treatment to minimize serum-dependent effects on wound closure.

Images were captured at 0, 24, and 48 h using a light microscope (EVOS XL Core, Invitrogen by Thermo Fisher Scientific, Carlsbad, CA, USA).

Wound closure was calculated as the percentage reduction in wound width relative to the initial wound width (0 h), using the following formula:

Wound closure (%) = [(D_0_ − D_t_)/D_0_] × 100

where D_0_ represents the initial wound width at 0 h and D_t_ represents the wound width measured after 24 or 48 h.

The calculated values were presented directly as percentage wound closure, without any additional normalization to the untreated control or DOX-treated groups.

### 4.7. Oil Red O Staining and Quantification of Lipid Accumulation

Lipid accumulation was evaluated using Oil Red O (ORO) staining. Cells were washed three times with PBS (pH 7.4) and fixed with 10% neutral-buffered formalin at room temperature (22–24 °C) for 30 min. Cells were then incubated with 60% isopropanol for 5 min. The ORO stock solution was prepared by dissolving 0.4 g ORO powder (Merck, O0625-25G) in 100 mL of 99% isopropanol overnight and filtered through a 0.2 µm filter. A working solution was freshly prepared by mixing the stock solution with distilled water (3:2, *v*/*v*) and filtered before use. Cells were stained for 15 min at room temperature, washed with distilled water, and air-dried. Images were captured using an inverted light microscope (Evos XL Core, Invitrogen by Thermo Fisher Scientific) equipped with a digital camera at 200× magnification.

The number of LDs per cell was quantified from ORO-stained images using ImageJ software (version 1.54u9, National Institutes of Health, Bethesda, MD, USA). Images were converted to 8-bit grayscale, and a uniform threshold was applied to detect ORO-positive droplets. LDs were quantified using the Analyze Particles function. Cell number was determined by manual counting from the same fields of view. The same threshold settings were applied to all analyzed images. Results were expressed as LDs per cell. For each condition, at least three randomly selected fields were analyzed.

For semi-quantitative analysis, the bound dye was extracted using 99% isopropanol, and absorbance was measured at 492 nm using a microplate reader (Mobi µ2 MicroDigital plate reader, MicroDigital Co., Seoul, Republic of Korea). Oil Red O staining was expressed as absorbance values measured at 492 nm.

### 4.8. Protein Quantification

Protein concentrations were determined using the Pierce™ 660 nm Protein Assay (22660, Thermo Fisher Scientific) with Pre-Diluted Protein Assay Standards, Bovine Serum Albumin (BSA) Set (23208, Thermo Fisher Scientific) as a standard.

### 4.9. Western Blot Analysis

Cells were lysed in ice-cold Cell Extraction Buffer (FNN0011, Invitrogen) supplemented with 1 mM phenylmethanesulfonyl fluoride (PMSF, 36978, Thermo Fisher Scientific) and Halt™ Protease Inhibitor Cocktail (1862209, Thermo Fisher Scientific). Lysates were incubated on ice for 30 min with gentle mixing every 10 min and centrifuged at 15,000 rpm for 10 min at 4 °C. The supernatants were collected, and protein concentrations were determined using the Pierce™ 660 nm Protein Assay.

Equal amounts of protein (25 µg per sample) were mixed with 2× Laemmli Sample Buffer (#161-0737, Bio-Rad), denatured at 95 °C for 10 min, and separated on Mini-PROTEAN TGX Stain-Free 4–15% polyacrylamide gels (#4568084, Bio-Rad). Proteins were transferred onto PVDF membranes (Trans-Blot Turbo Mini PVDF Membranes, #10026934, Bio-Rad).

Membranes were blocked using EveryBlot Blocking Buffer (#12010020, Bio-Rad) and incubated overnight at 4 °C with the appropriate primary antibodies. Following washing, membranes were incubated with the corresponding horseradish peroxidase (HRP)-conjugated secondary antibodies. Protein bands were detected using Clarity Max ECL substrate (#1705062, Bio-Rad) and visualized with a ChemiDoc Imaging System (Bio-Rad). Densitometric analysis was performed using Image Lab software (version 6.1, Bio-Rad Laboratories, Hercules, CA, USA). Protein loading and transfer quality were assessed using Stain-Free imaging of the gels and corresponding PVDF membranes after transfer. Target protein band intensities were normalized to the total protein signal measured in the corresponding membrane lanes using Stain-Free technology. This normalization approach was applied consistently to all Western blot analyses presented in the manuscript. GAPDH was additionally included in the Western blot images as a supplementary reference where reliable detection was available but was not used for densitometric normalization.

### 4.10. Statistical Analysis

Statistical analyses were performed using GraphPad Prism version 11.02 (GraphPad Software, La Jolla, CA, USA). Data are presented as mean ± SD from three independent biological experiments. Technical replicates were averaged within each biological experiment. Comparisons between relevant treatment groups were performed separately for each cell line and experimental condition using one-way ANOVA followed by Tukey’s multiple-comparison test. Differences were considered statistically significant at *p* < 0.05.

### 4.11. Experimental Design

**Figure 12:**
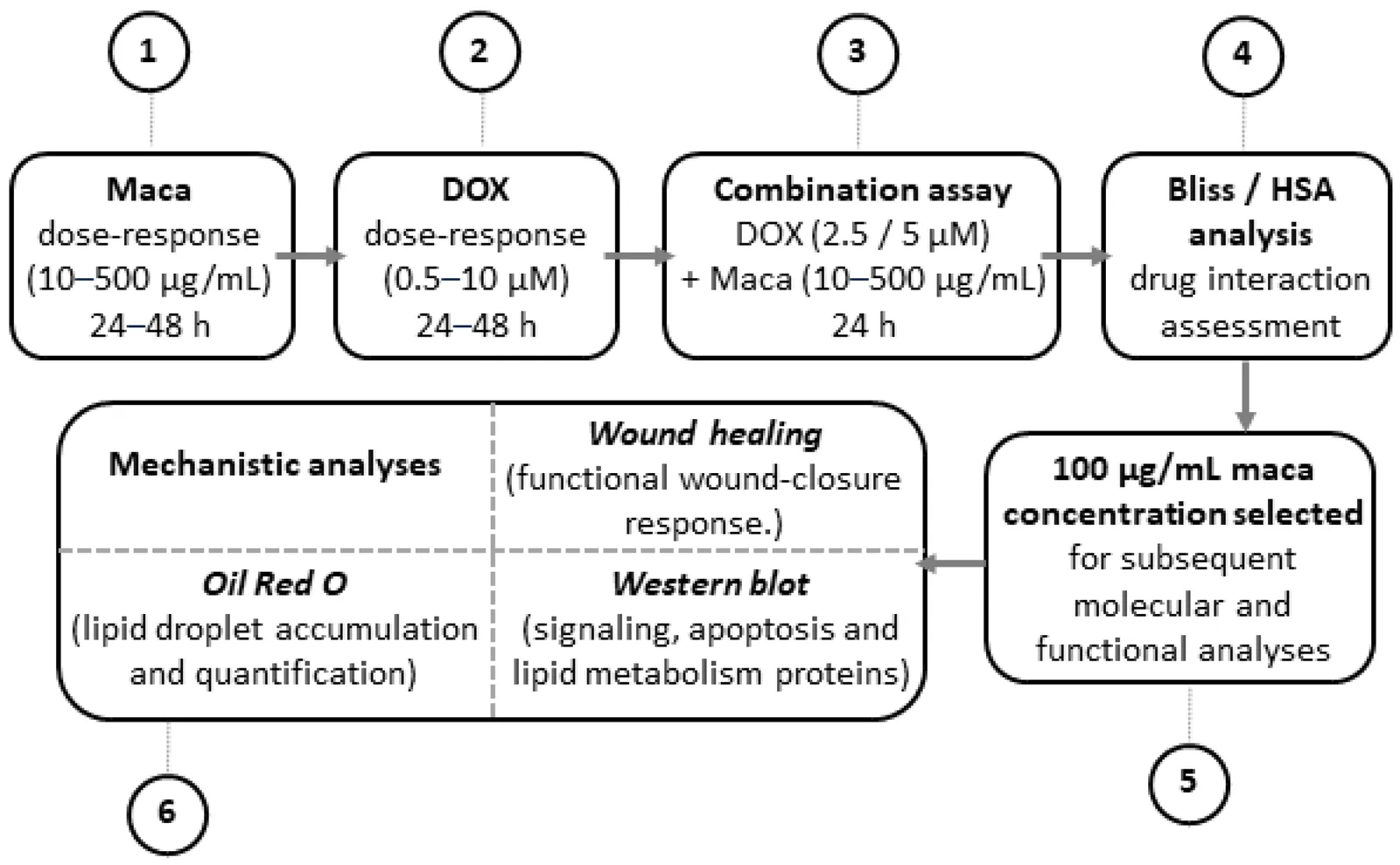
Experimental workflow of the study. Dose-response analyses were first performed for morphotype-specific maca preparations (10–500 µg/mL; 24–48 h) and doxorubicin (DOX; 0.5–10 µM; 24–48 h). Combination experiments were subsequently conducted using DOX at 2.5 and 5 µM together with increasing concentrations of maca preparations (10–500 µg/mL) for 24 h. Interaction analyses using the Bliss independence and Highest Single Agent (HSA) models were performed for maca concentrations of 10–200 µg/mL. Based on the dose-response results, 100 µg/mL maca preparation was selected for subsequent analyses of treatment-associated changes in protein expression, lipid droplet accumulation, and wound closure. These included Western blot analysis of proteins involved in signaling, apoptosis, and lipid metabolism, Oil Red O staining for lipid droplet accumulation and quantification, and wound healing assays for the assessment of treatment-associated changes in wound closure.

The study was performed in six sequential steps, as summarized in Figure 12: (i) determination of dose–response curves for morphotype-specific maca preparations; (ii) determination of dose–response curves for DOX; (iii) evaluation of combined DOX–maca treatment using two selected DOX concentrations (2.5 and 5 µM) and increasing concentrations of maca preparations; (iv) assessment of treatment interactions using the Bliss independence and HSA models; (v) selection of 100 µg/mL maca preparations for subsequent molecular and functional analyses; and (vi) evaluation of treatment-associated changes using Western blotting, Oil Red O staining, and wound closure assays. Western blotting was used to assess apoptosis-associated, survival-related, and lipid metabolism-related proteins, Oil Red O staining was used to evaluate lipid accumulation, and the wound closure assay was used to assess the functional response to treatment.

## 5. Conclusions

The three commercial maca preparations investigated in this study (MY, MR, and MB) differentially modified pancreatic cancer cell responses to DOX, with effects varying according to the preparation, cell line, and DOX concentration. Favorable interactions identified by Bliss independence and HSA analyses were most consistently observed in PANC-1 cells treated with MR or MB in combination with 2.5 µM DOX and in BxPC-3 cells at 5 µM DOX, whereas MIA PaCa-2 cells predominantly showed neutral or less-than-additive interactions.

DOX–maca co-treatment was accompanied by reduced PI3K and FAP protein levels across all three cell lines, differential modulation of FOXO1 and CASP3, and cell line-dependent alterations in lipid droplet accumulation and the lipid metabolism-related proteins ATGL, ABHD5, and PLIN3. These findings indicate that the responses to the tested maca preparations are associated with changes in survival-related signaling, apoptosis-associated responses, and lipid metabolic homeostasis, although the causal relationships among these processes remain to be established.

Overall, the results demonstrate that the biological effects of the tested maca preparations vary according to the specific preparation and pancreatic cancer cell context. These findings support further investigation of maca preparations as complex natural products capable of modifying experimental responses to anticancer drug-induced stress. Future studies integrating batch-specific phytochemical characterization, functional mechanistic validation, clinically relevant pancreatic cancer treatment regimens, and advanced preclinical models will be required to determine the reproducibility and translational potential of these observations.

## Figures and Tables

**Table 1:** Calculated IC_25_ and IC_50_ values for Maca Yellow (MY), Maca Red (MR) and Maca Black (MB) in BxPC-3, MIA PaCa-2 and PANC-1 pancreatic cancer cells. Inhibitory concentrations were determined from dose–response curves after 24 h and 48 h of treatment. Data were derived from three independent biological experiments, each performed with four technical replicates (*n* = 3 biological experiments).

Cell Line	Maca	Incubation Time	IC_25_ (µg/mL)	IC_50_ (µg/mL)
BxPC-3	MY	24 h	227.41	397.48
MIA PaCa-2	MY	24 h	124.16	266.78
PANC-1	MY	24 h	238.60	430.47
BxPC-3	MY	48 h	196.35	408.57
MIA PaCa-2	MY	48 h	139.25	303.08
PANC-1	MY	48 h	50.00	237.27
BxPC-3	MR	24 h	212.74	384.92
MIA PaCa-2	MR	24 h	139.00	285.88
PANC-1	MR	24 h	261.27	452.99
BxPC-3	MR	48 h	248.78	409.86
MIA PaCa-2	MR	48 h	153.75	319.87
PANC-1	MR	48 h	85.40	262.71
BxPC-3	MB	24 h	225.93	394.28
MIA PaCa-2	MB	24 h	116.65	265.11
PANC-1	MB	24 h	259.83	451.84
BxPC-3	MB	48 h	294.83	476.91
MIA PaCa-2	MB	48 h	171.63	327.49
PANC-1	MB	48 h	74.11	243.84

**Table 2:** Geographical and phenotype-dependent variation in the phytochemical composition of Peruvian and Asian-grown maca.

Phytochemical Class/Marker	Peruvian Maca	Asian-Grown Maca	References
Macamides	Total macamides: yellow 393.30 ± 2.42; black 269.10 ± 1.03 µg/g. Separate field study (µmol/g dry matter): yellow 0.10–1.12; pink 0.07–1.98; violet 0–1.87; lead-coloured 0.06–1.46. Spray-dried extracts: higher relative levels in black than red maca.	China: purple 1845.75 ± 3.45; yellow 1090.20 ± 2.07 µg/g. Separate study: black 0.15%; other phenotypes 0.23–0.29%.	[31,33,34,35,36]
Macaenes	5-Oxo-6E,8E-octadecadienoic acid (µmol/g dry matter): yellow 0.27–0.83; pink 0.30–2.67; violet 0.07–1.78; lead-coloured 0.28–1.73.	Tibet, China (µg/g): 9-oxo-10E,12Z-octadecadienoic acid—yellow 220.65–875.03; black 173.85–563.16; purple 338.18–600.06. 9-Oxo-10E,12E-octadecadienoic acid—yellow 185.17–476.05; black 190.08–365.56; purple 195.91–298.12.	[31,36,37]
Total glucosinolates	Fresh hypocotyls (g/100 g): black 1.684; red 1.656; yellow 0.193. Dried: black 0.085; red 0.357; yellow 0.153.	China: yellow and black 1.55%; white 0.93%; purple 0.76%.	[31,35,38]
Glucotropaeolin (GLCT)	Ancash, dried hypocotyls (g/100 g): purple 0.641; black 0.164; red 0.103; yellow 0.033.	Shangri-La, China (g/100 g): H-shaped—black 2.229; purple 1.165; yellow 0.672. G-shaped—yellow 1.760; black 1.030; purple 0.873.	[31,32]
*m*-Methoxyglucotropaeolin (MGLCT)	Ancash, dried hypocotyls (g/100 g): purple 0.232; red 0.144; yellow 0.143; black 0.125.	Shangri-La, China: not detected in yellow, purple, or black maca, regardless of hypocotyl shape.	[31,32]
Total phenolic compounds	Red 225.05 ± 0.65; yellow 193.85 ± 0.90; black 171.60 ± 0.64 mg GAE/100 g dry weight.	Fukushima, Japan (mmol/L): yellow-purple 65.1; white-purple 64.6; deep-purple 61.7; yellow 46.4; white 46.7; black 35.8.	[31,39,40]

Abbreviations: GAE, gallic acid equivalent. Data were obtained from independent studies involving different maca phenotypes, geographical origins, sample-processing conditions, and analytical methods. Therefore, direct quantitative comparisons across studies are limited. The reported values do not represent measurements of the maca batches investigated in the present study.

## Data Availability

The data supporting the findings of this study are available from the corresponding author upon reasonable request.

## Supplementary Materials

The following supporting information can be downloaded at https://www.mdpi.com/article/10.3390/molecules31214011/s1, Figure S1: Effects of doxorubicin (DOX) on pancreatic cancer cell viability. BxPC-3 cells were treated with increasing concentrations of DOX for 24 h (A) or 48 h (B). MIA PaCa-2 cells were treated for 24 h (C) or 48 h (D). PANC-1 cells were treated for 24 h (E) or 48 h (F). Cell viability was assessed following treatment. As a vehicle control, DMSO was used at the same concentration as that present in the highest DOX dose. Data are presented as mean ± SD from three independent biological experiments, each performed with four technical replicates (n = 3 biological experiments). * *p* < 0.05 vs. 0 µM DOX. Figure S2: Representative microscopy images of the wound healing assay in BxPC-3 pancreatic cancer cells following doxorubicin (DOX) and maca co-treatment. Images show wound closure at 0, 24, and 48 h following treatment with (A) 2.5 µM DOX or (B) 5 µM DOX alone or in combination with Maca Yellow (MY), Maca Red (MR), or Maca Black (MB) (100 µg/mL). Untreated cells served as the control. Representative micrographs illustrate treatment-dependent changes in wound closure compared to DOX monotherapy and control conditions. Figure S3: Representative microscopy images of the wound healing assay in MIA PaCa-2 pancreatic cancer cells following doxorubicin (DOX) and maca co-treatment. Images show wound closure at 0, 24, and 48 h following treatment with (A) 2.5 µM DOX or (B) 5 µM DOX alone or in combination with Maca Yellow (MY), Maca Red (MR), or Maca Black (MB) (100 µg/mL). Untreated cells served as the control. Representative micrographs illustrate treatment-dependent changes in wound closure compared to DOX monotherapy and control conditions. Figure S4: Representative microscopy images of the wound healing assay in PANC-1 pancreatic cancer cells following doxorubicin (DOX) and maca co-treatment. Images show wound closure at 0, 24, and 48 h following treatment with (A) 2.5 µM DOX or (B) 5 µM DOX alone or in combination with Maca Yellow (MY), Maca Red (MR), or Maca Black (MB) (100 µg/mL). Untreated cells served as the control. Representative micrographs illustrate treatment-dependent changes in wound closure compared to DOX monotherapy and control conditions. Figure S5: Representative Oil Red O staining of lipid droplets (LDs) in BxPC-3 pancreatic cancer cells following doxorubicin (DOX) and maca co-treatment. Cells cultured in 1% FBS served as the control for reduced-serum conditions (Control 1% FBS), while 10% FBS represented standard growth conditions (Control 10% FBS). Treatment with (A) 2.5 µM DOX or (B) 5 µM DOX alone or in combination with Maca Yellow (MY), Maca Red (MR), or Maca Black (MB) (100 µg/mL) was performed. LDs were visualized by Oil Red O staining (red signal). Representative micrographs illustrate treatment-dependent changes in intracellular lipid accumulation, with combined DOX-maca treatment showing altered lipid droplet content compared to DOX alone and control conditions. Figure S6: Representative Oil Red O staining of lipid droplets (LDs) in MIA PaCa-2 pancreatic cancer cells following doxorubicin (DOX) and maca co-treatment. Cells cultured in 1% FBS served as the control for reduced-serum conditions (Control 1% FBS), while 10% FBS represented standard growth conditions (Control 10% FBS). Treatment with (A) 2.5 µM DOX or (B) 5 µM DOX alone or in combination with Maca Yellow (MY), Maca Red (MR), or Maca Black (MB) (100 µg/mL) was performed. LDs were visualized by Oil Red O staining (red signal). Representative micrographs illustrate treatment-dependent changes in intracellular lipid accumulation, with combined DOX-maca treatment showing altered lipid droplet content compared to DOX alone and control conditions. Figure S7: Representative Oil Red O staining of lipid droplets (LDs) in PANC-1 pancreatic cancer cells following doxorubicin (DOX) and maca co-treatment. Cells cultured in 1% FBS served as the control for reduced-serum conditions (Control 1% FBS), while 10% FBS represented standard growth conditions (Control 10% FBS). Treatment with (A) 2.5 µM DOX or (B) 5 µM DOX alone or in combination with Maca Yellow (MY), Maca Red (MR), or Maca Black (MB) (100 µg/mL) was performed. LDs were visualized by Oil Red O staining (red signal). Representative micrographs illustrate treatment-dependent changes in intracellular lipid accumulation, with combined DOX-maca treatment showing altered lipid droplet content compared to DOX alone and control conditions. Table S1: Calculated IC_25_ and IC_50_ values for doxorubicin (DOX) in BxPC-3, MIA PaCa-2 and PANC-1 pancreatic cancer cells. Inhibitory concentrations were determined from dose-response curves after 24 h and 48 h of treatment. Values were derived from three independent biological experiments, each performed with four technical replicates. Table S2: Complete Bliss independence, Highest Single Agent (HSA), and DOX enhancement analysis for combinations of doxorubicin and morphotype-specific maca preparations in BxPC-3, MIA PaCa-2, and PANC-1 pancreatic cancer cells after 24 h of treatment. Values represent means and corresponding standard deviations from three independent biological experiments.

## Abbreviations

The following abbreviations are used in this manuscript:

ABHD5	α/β-hydrolase domain-containing protein 5
AKT	protein kinase B
ATGL	adipose triglyceride lipase
CASP3	caspase-3
DOX	doxorubicin
FAP	fibroblast activation protein
FOXO1	forkhead box O1
GAPDH	glyceraldehyde-3-phosphate dehydrogenase
HPGe	high-purity germanium (detector)
KRAS	Kirsten rat sarcoma viral oncogene homolog
LDs	lipid droplets
MAPK	mitogen-activated protein kinase
MB	maca black
MR	maca red
MY	maca yellow
PI3K	phosphoinositide 3-kinase
PLIN3	perilipin 3
