# Supplementary material for: Yellow, Red, and Black Lepidium meyenii Preparations Differentially Modulate Doxorubicin Response and Lipid Metabolism-Related Changes in Pancreatic Cancer Cells
Source: Molecules. 2026 Nov 6;31(21):4011. doi: 10.3390/molecules31214011 (PMC13765015; doi:10.3390/molecules31214011)

BxPc-3
Figure 7A

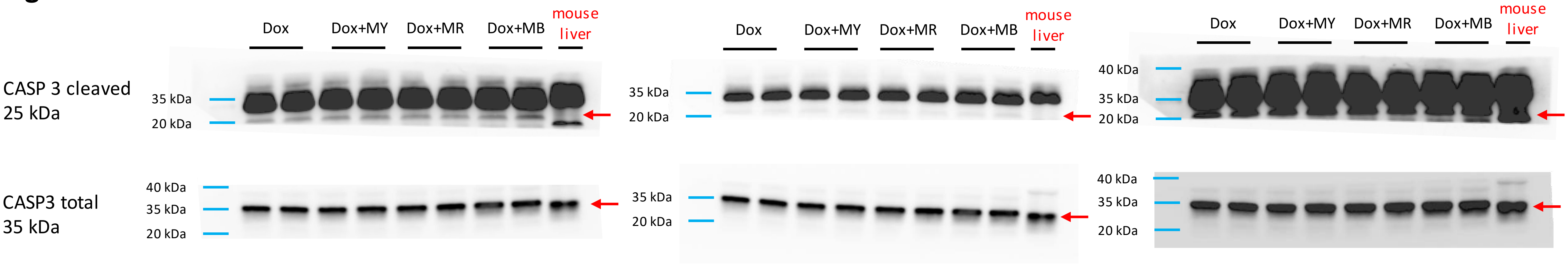

MIA PaCa2
Figure 7B

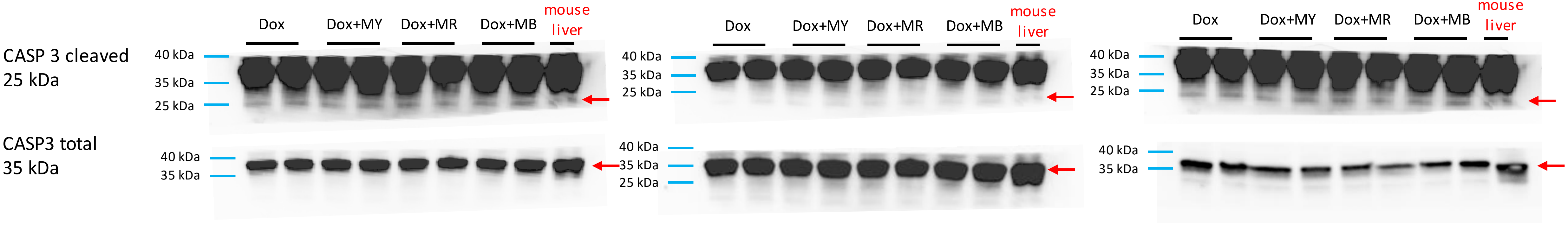

PANC-1
Figure 7C

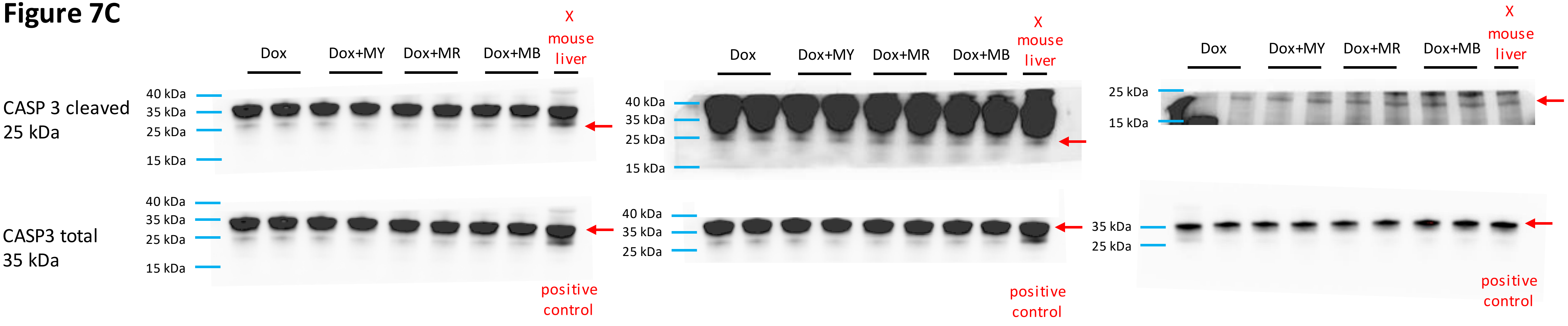

BxPc-3
Figure 8A

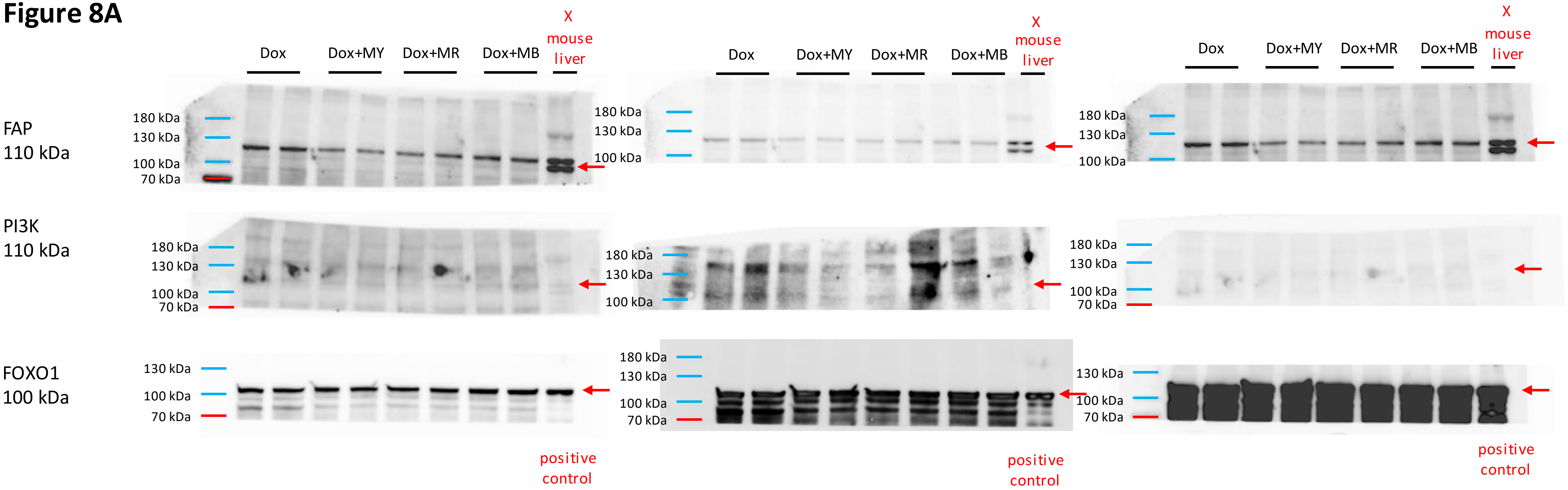

MIA PaCa2
Figure 8B

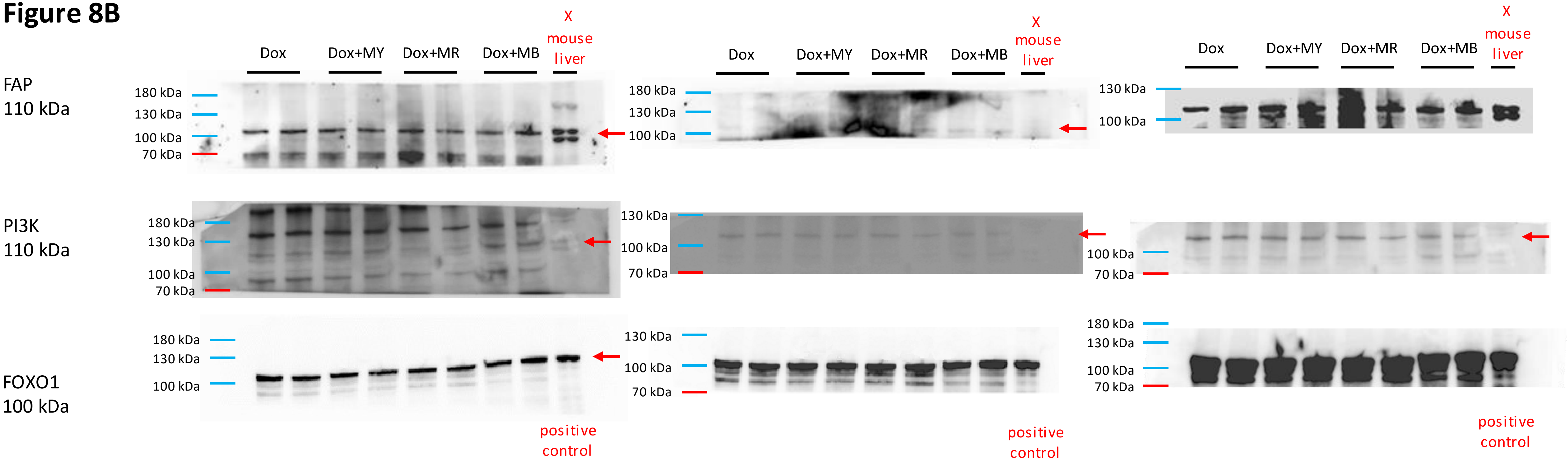

PANC-1
Figure 8C

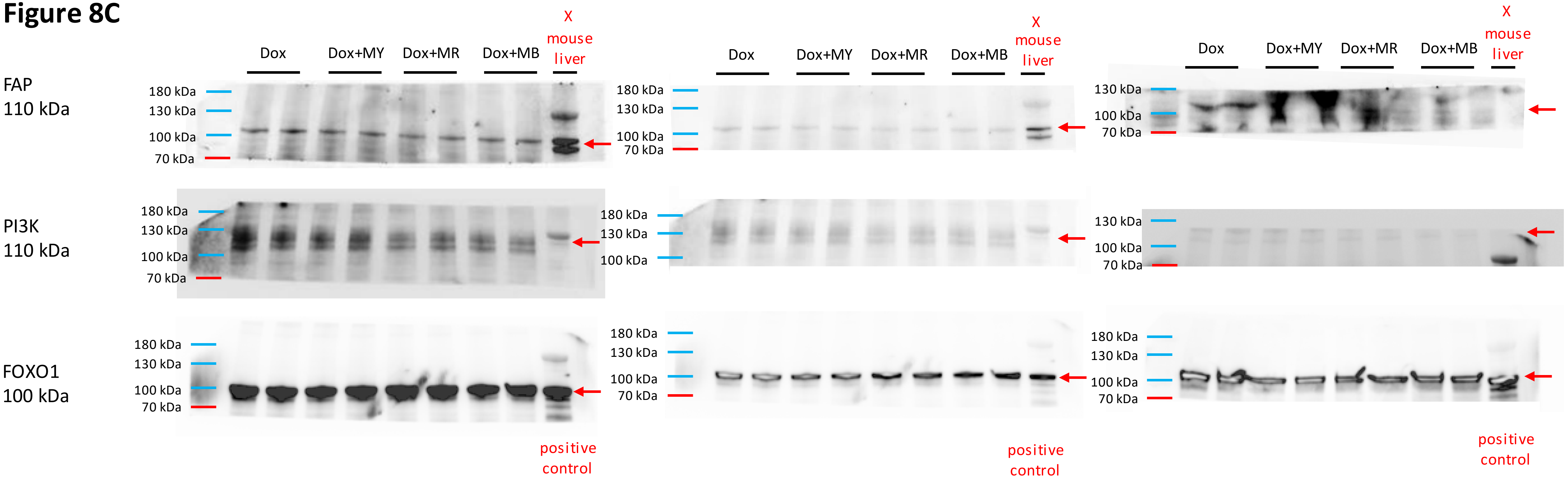

BxPc-3

Figure 10A

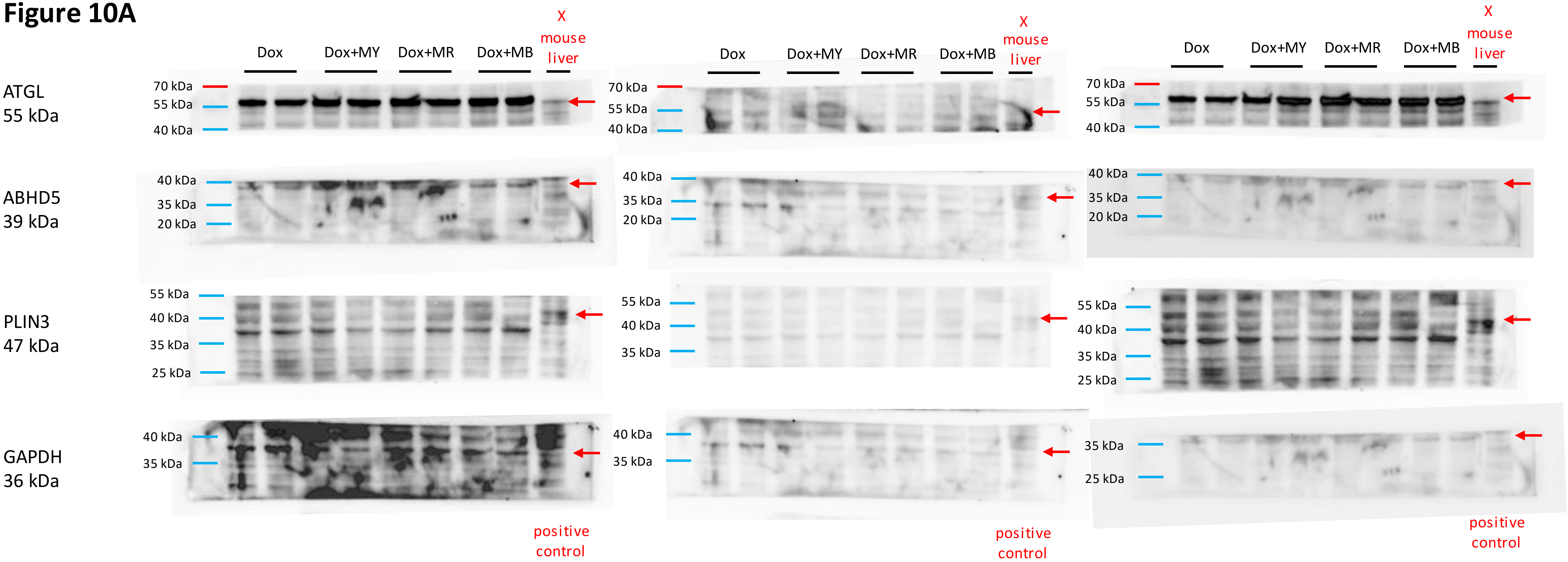

MIA PaCa2
Figure 10B

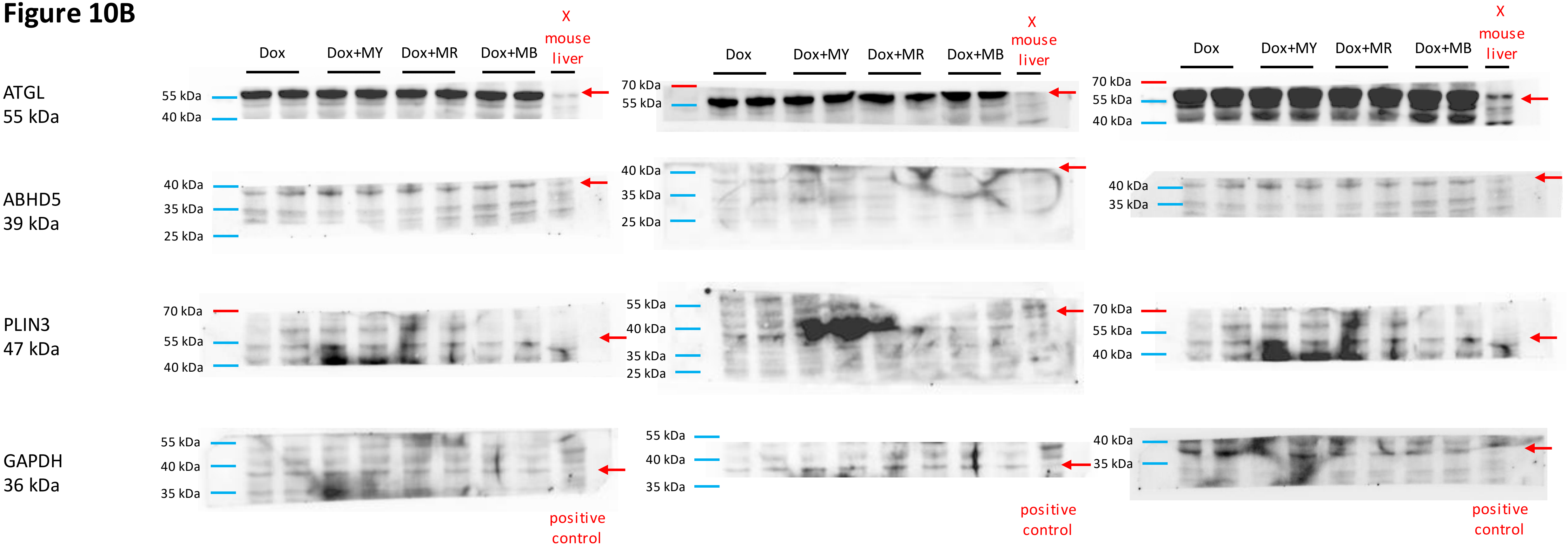

PANC-1

Figure 10C

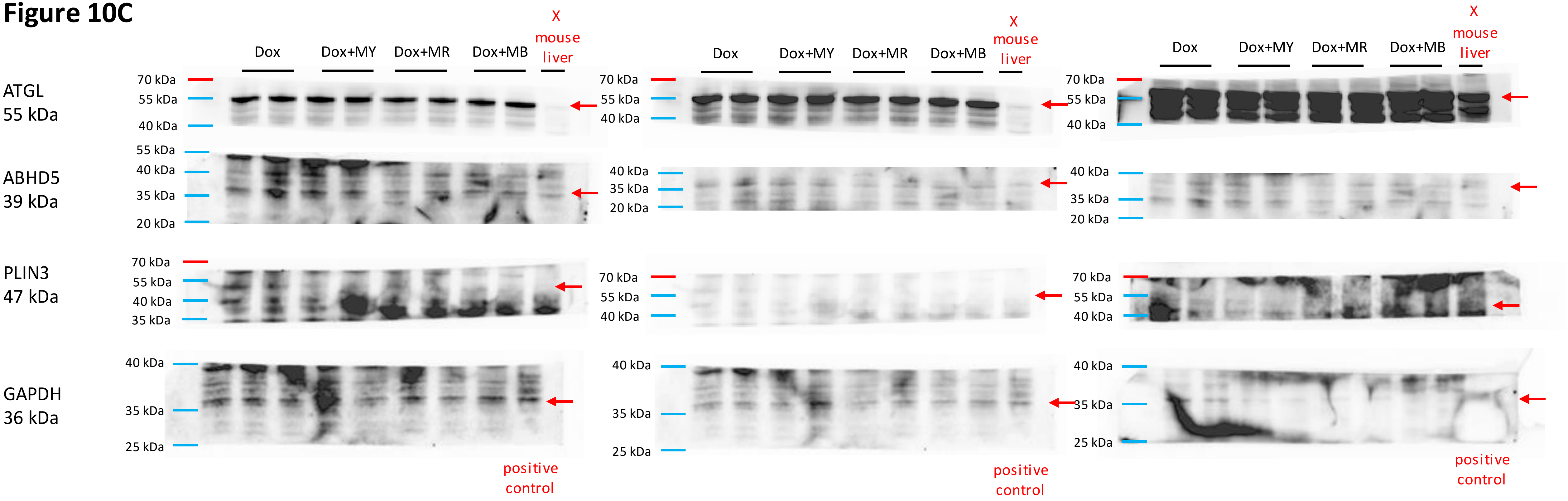

Full-length uncropped Western blot images for all Western blot experiments presented in the figures of this manuscript are provided together with the corresponding gel electrophoresis images, post-transfer membrane images, and membrane sectioning schemes used for protein detection. These data document protein loading, transfer efficiency, and the relationship between individual membrane sections and their corresponding protein targets.

Raw WB BxPc-3

gel electrophoresis

Gel 1

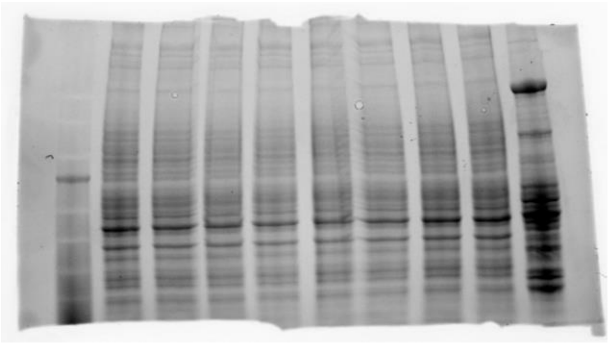

post-transfer membrane

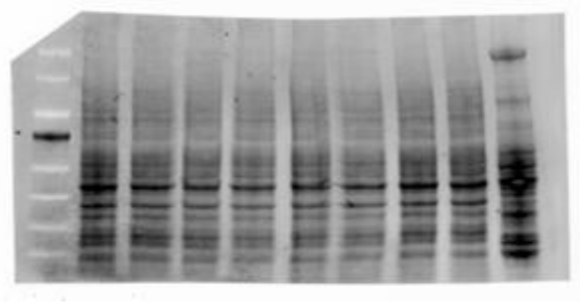

post-transfer membrane

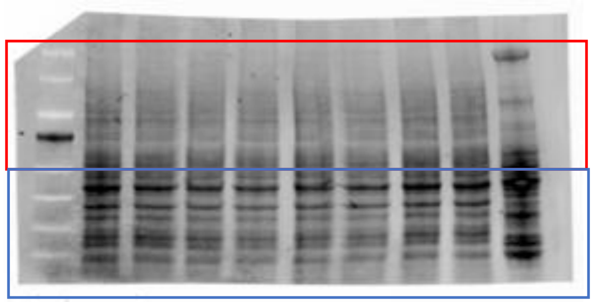

2.1

2.2

ChemiDoc image of the post-transfer membrane

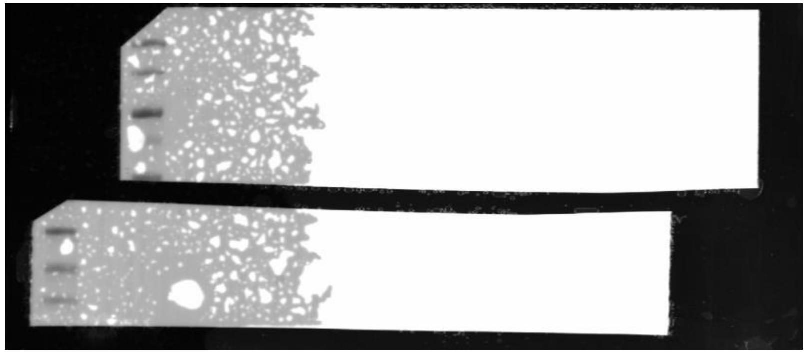

1.1

1.2

Abbreviations: Liver, mouse liver tissue (positive control), DOX, doxorubicin, MY, yellow maca, MR, red maca, MB, black maca,.

Raw WB BxPc-3

1.1 not used

1.2 GAPDH
anti-mouse

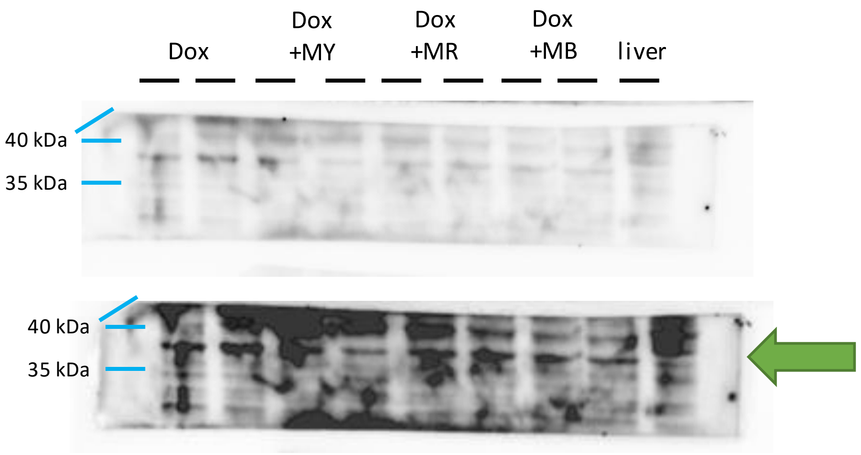

Used for Figure 7A, 8A
and 10A

1.1

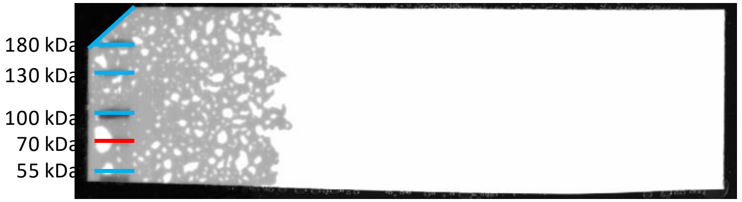

1.2

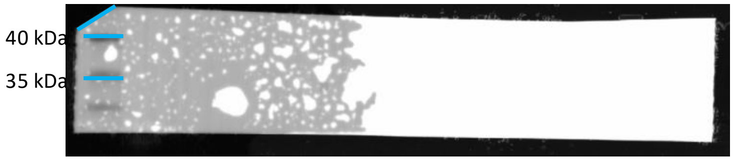

Raw WB BxPc-3

gel electrophoresis

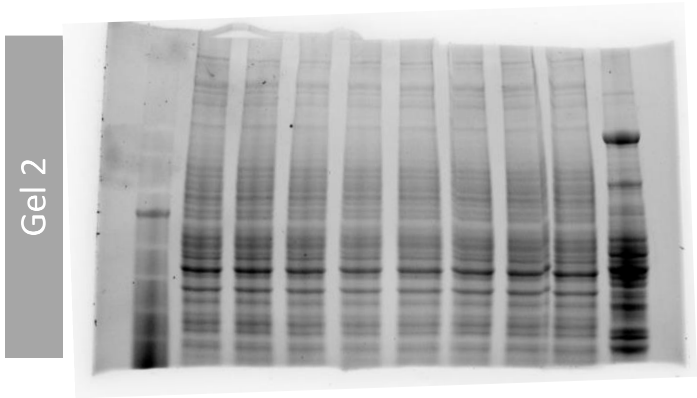

post-transfer membrane

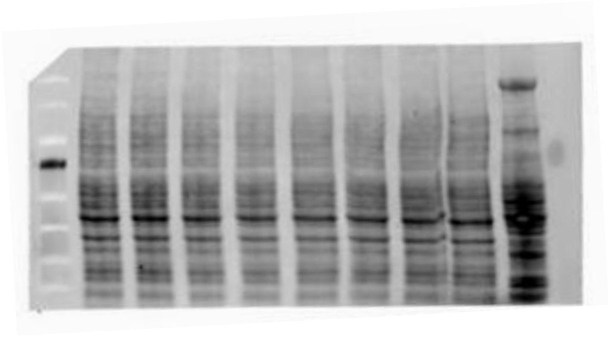

post-transfer membrane

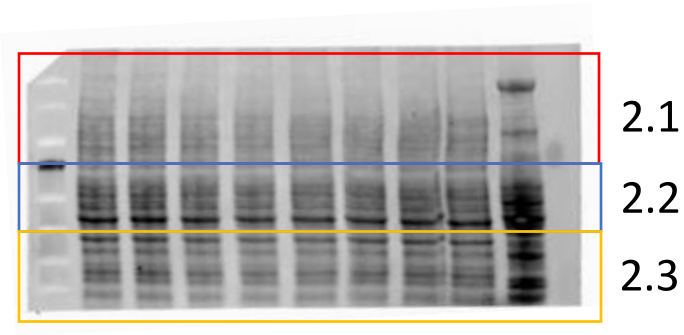

ChemiDoc image of the post-transfer membrane

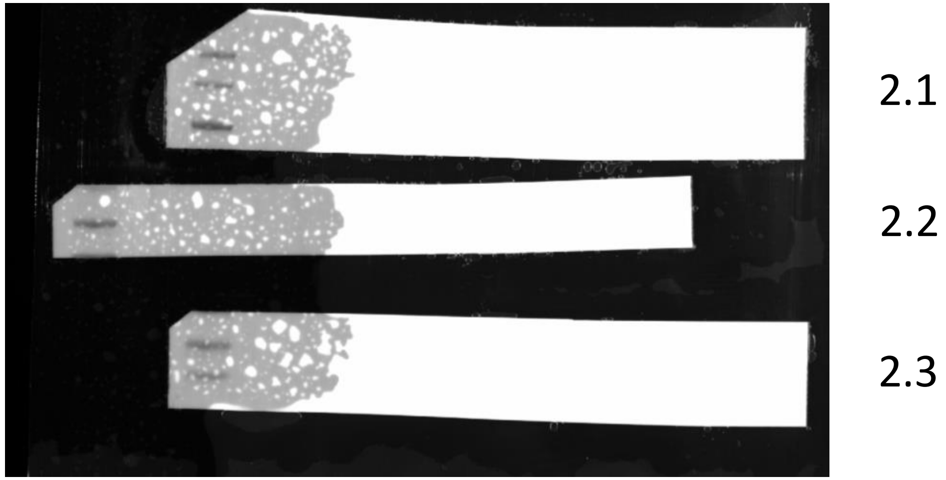

Abbreviations: Liver, mouse liver tissue (positive control), DOX, doxorubicin, MY, yellow maca, MR, red maca, MB, black maca,.

Raw WB BxPc-3

2.1 PI3K
anti-rabbit

Dox | Dox +MY | Dox +MR | Dox +MB | liver

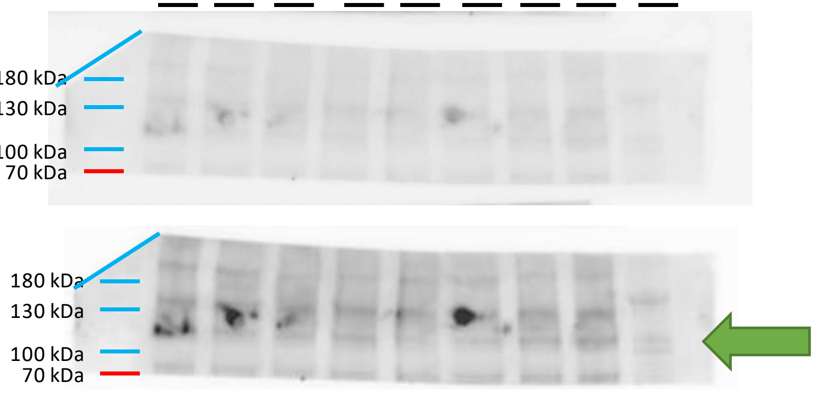

2.2 ATGL
anti-rabbit

Dox | Dox +MY | Dox +MR | Dox +MB | liver

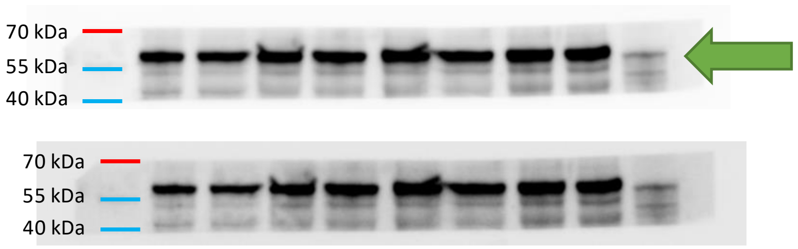

2.3 ABHD5
anti-mouse

Dox | Dox +MY | Dox +MR | Dox +MB | liver

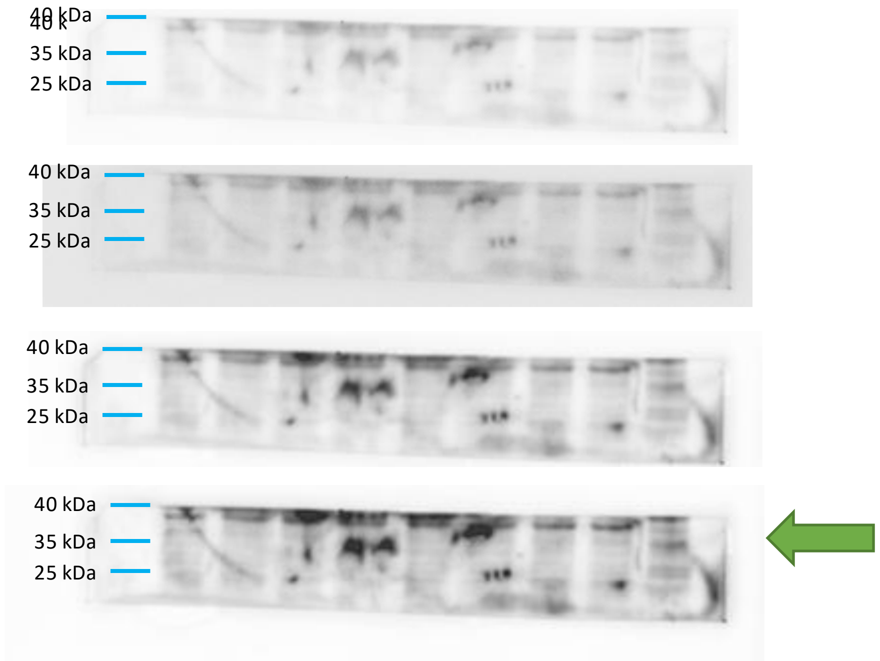

Used for Figure 8A

Used for Figure 10A

Used for Figure 10A

2.1

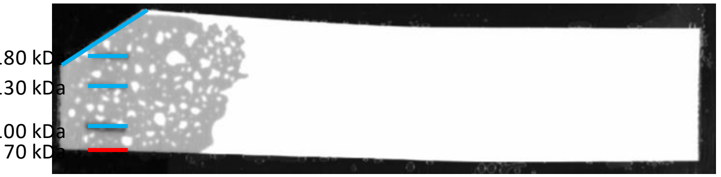

2.2

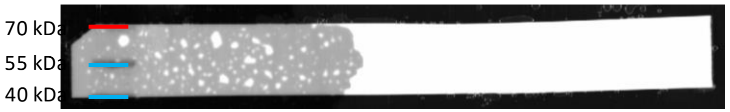

2.3

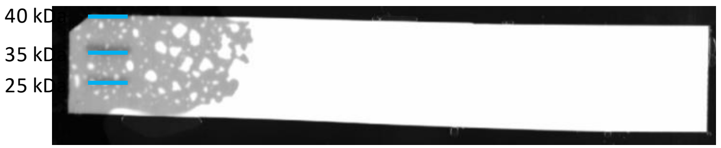

Raw WB BxPc-3

gel electrophoresis

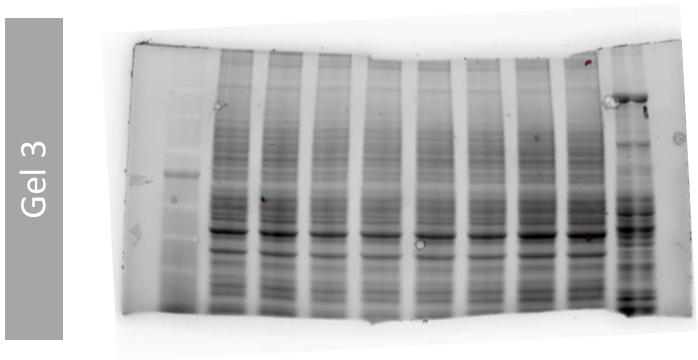

post-transfer membrane

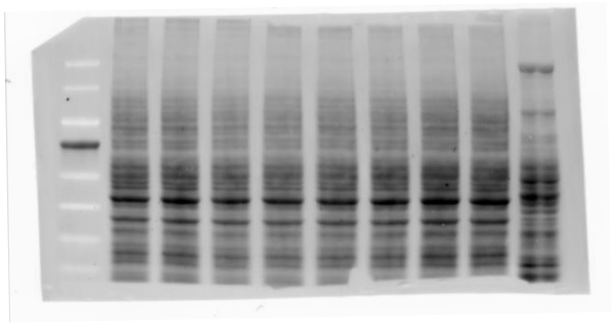

post-transfer membrane

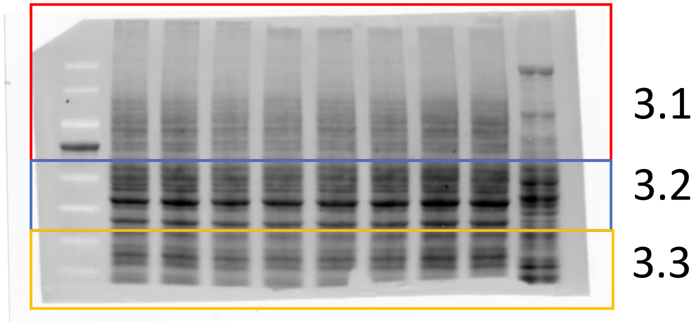

ChemiDoc image of the post-transfer membrane

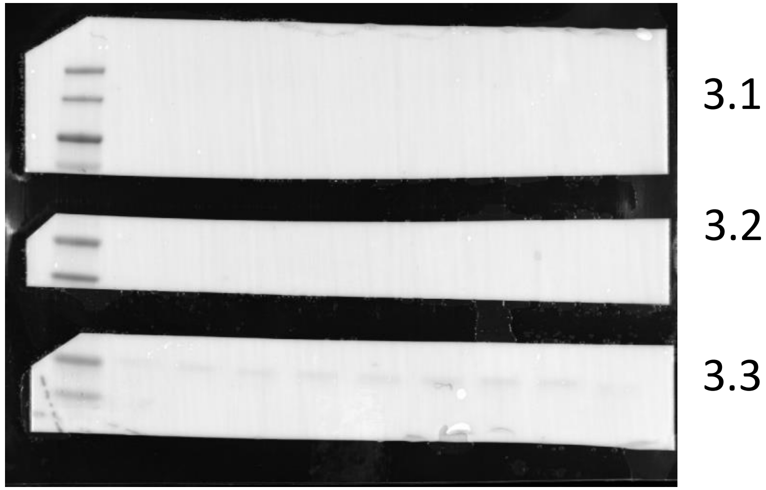

Abbreviations: Liver, mouse liver tissue (positive control), DOX, doxorubicin, MY, yellow maca, MR, red maca, MB, black maca,.

Raw WB BxPc-3

3.1 FAP
anti-rabbit

Dox | Dox +MY | Dox +MR | Dox +MB | liver

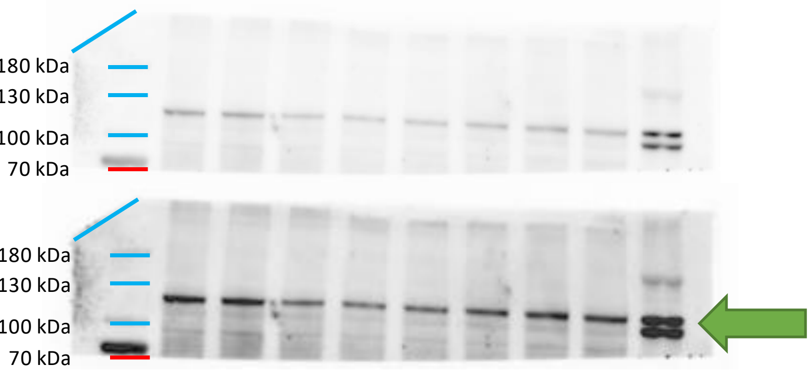

3.2 not used

Dox | Dox +MY | Dox +MR | Dox +MB | liver

3.3 CASP3
anti-rabbit

Dox | Dox +MY | Dox +MR | Dox +MB | liver

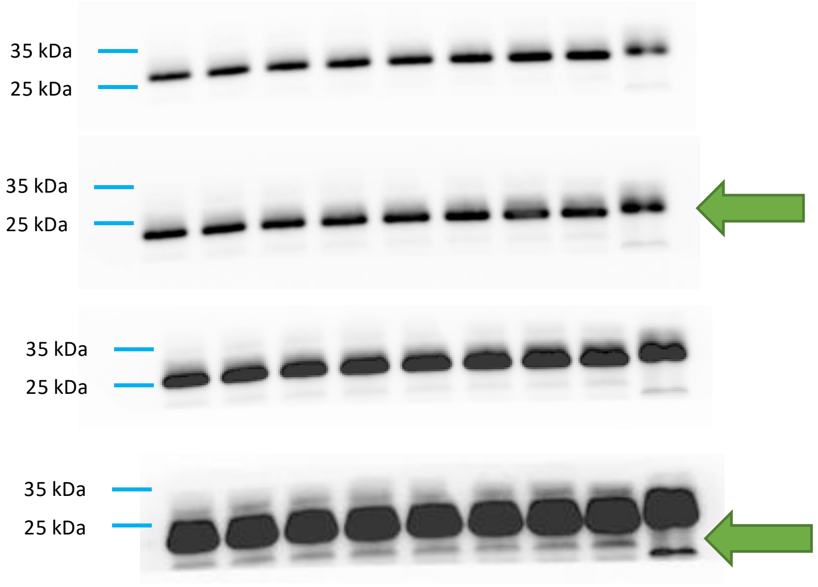

Used for Figure 8A

Not used

Used for Figure 7A

3.1

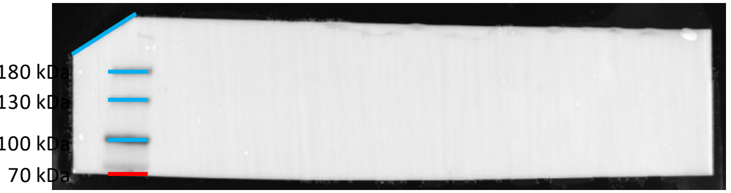

3.2

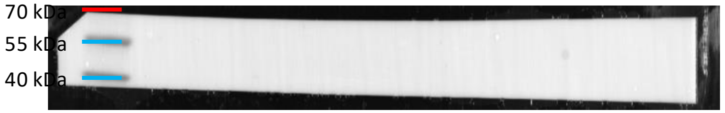

3.3

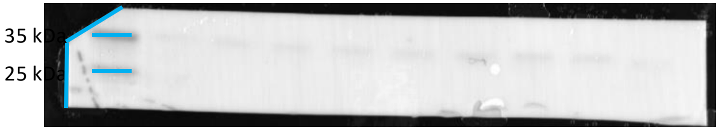

Raw WB BxPc-3

gel electrophoresis

Gel 4

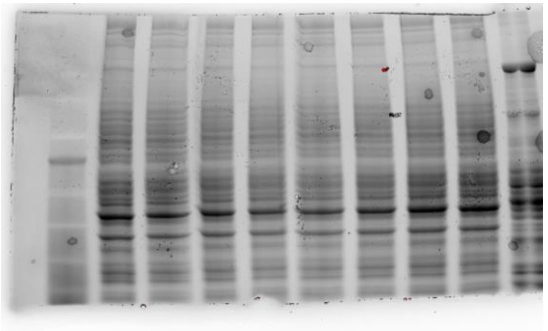

post-transfer membrane

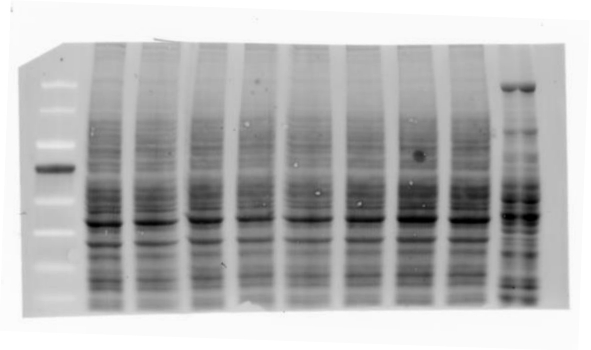

post-transfer membrane

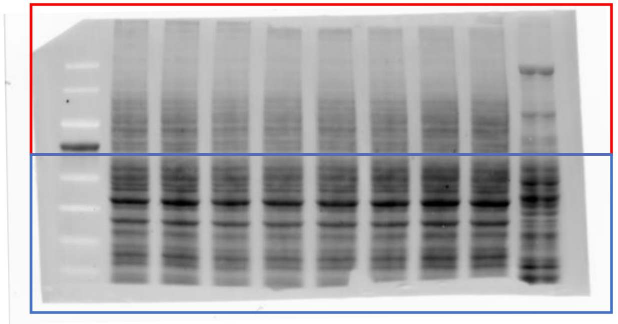

4.1

4.2

ChemiDoc image of the post-transfer membrane

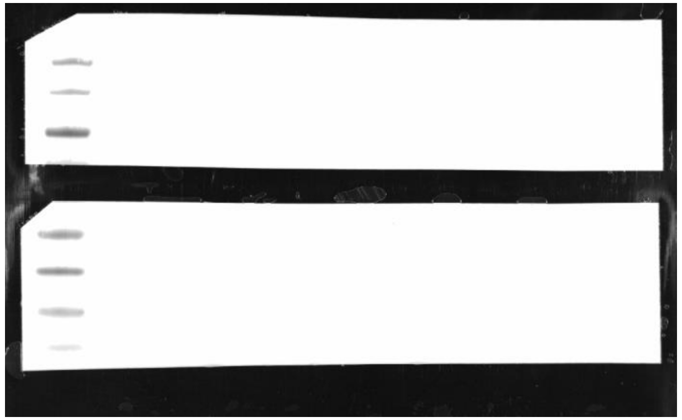

4.1

4.2

Abbreviations: Liver, mouse liver tissue (positive control), DOX, doxorubicin, MY, yellow maca, MR, red maca, MB, black maca,.

Raw WB BxPc-3

4.1 FOXO1
anti-rabbit

4.2 PLIN3
anti-mouse

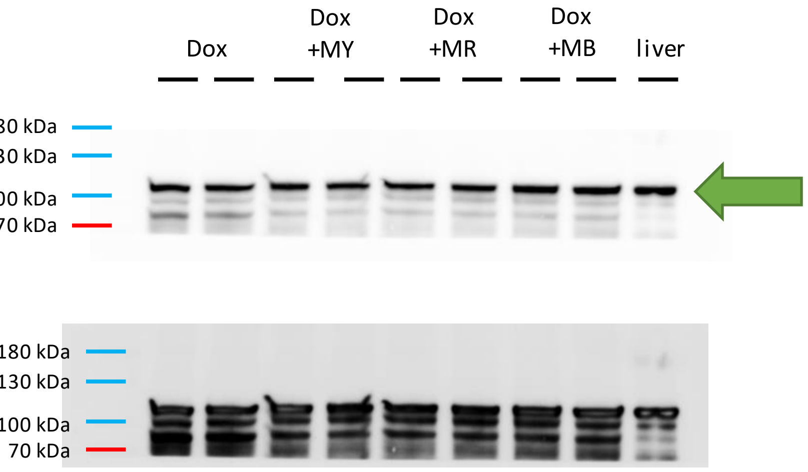

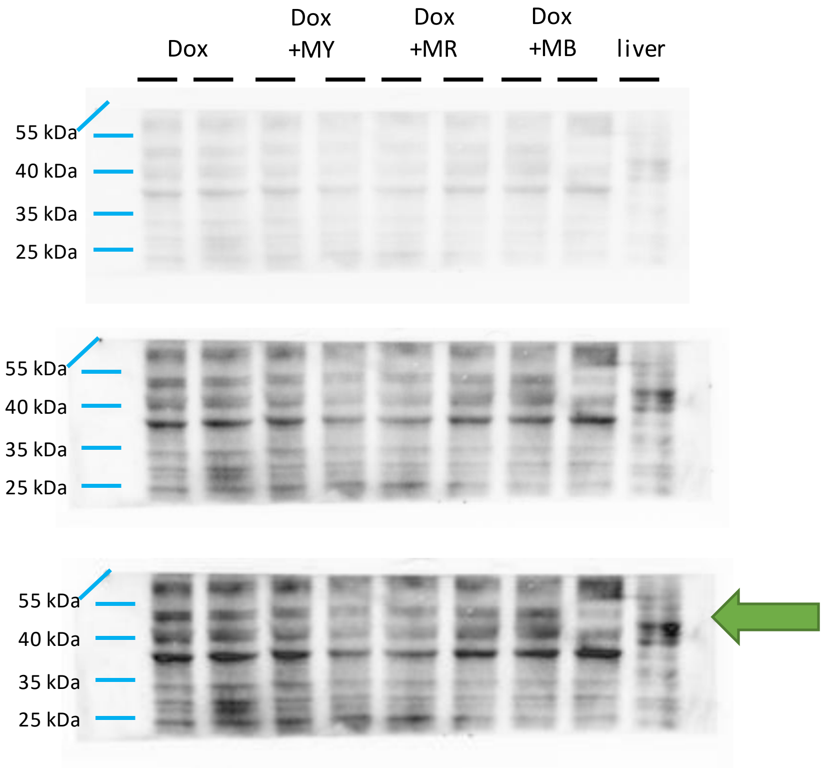

Used for Figure 8A

Used for Figure 10A

4.1

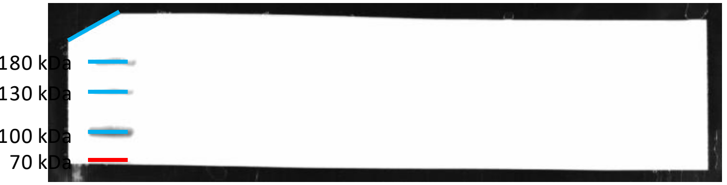

4.2

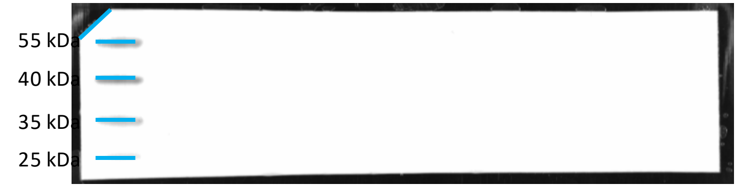

Raw WB MIA PaCa-2

gel electrophoresis

Gel 5

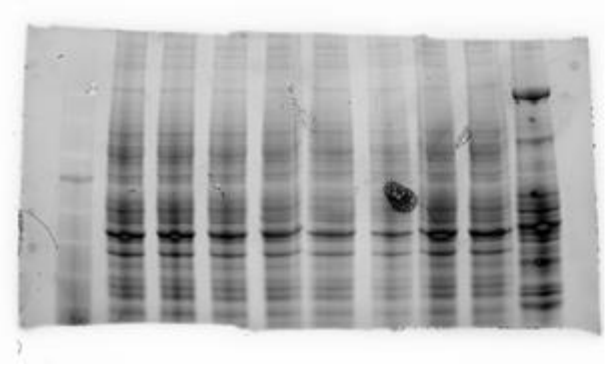

post-transfer membrane

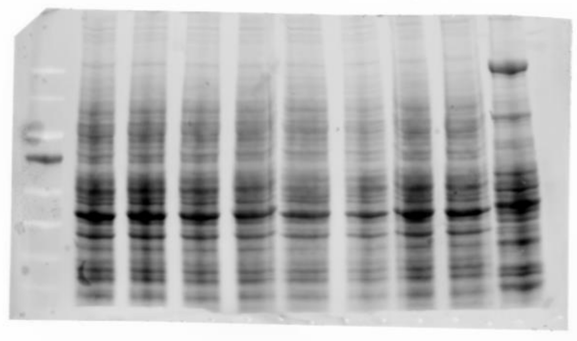

post-transfer membrane

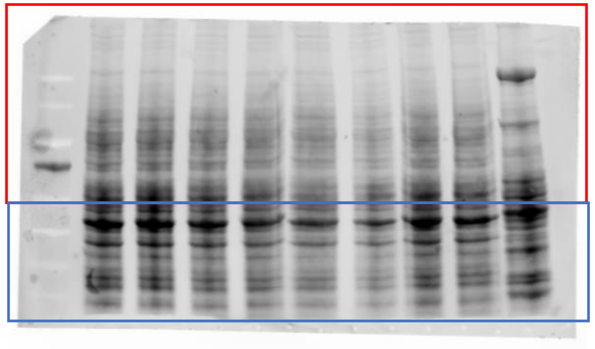

5.1

5.2

ChemiDoc image of the post-transfer membrane

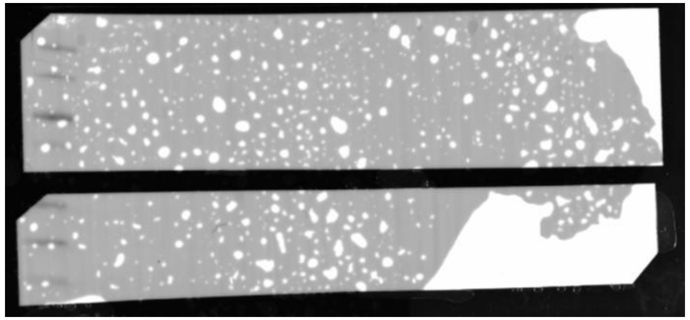

5.1

5.2

Abbreviations: Liver, mouse liver tissue (positive control), DOX, doxorubicin, MY, yellow maca, MR, red maca, MB, black maca,.

Raw WB MIA PaCa-2

5.1 not used

Dox | Dox +MY | Dox +MR | Dox +MB | liver

5.2 GAPDH
anti-mouse

Dox | Dox +MY | Dox +MR | Dox +MB | liver

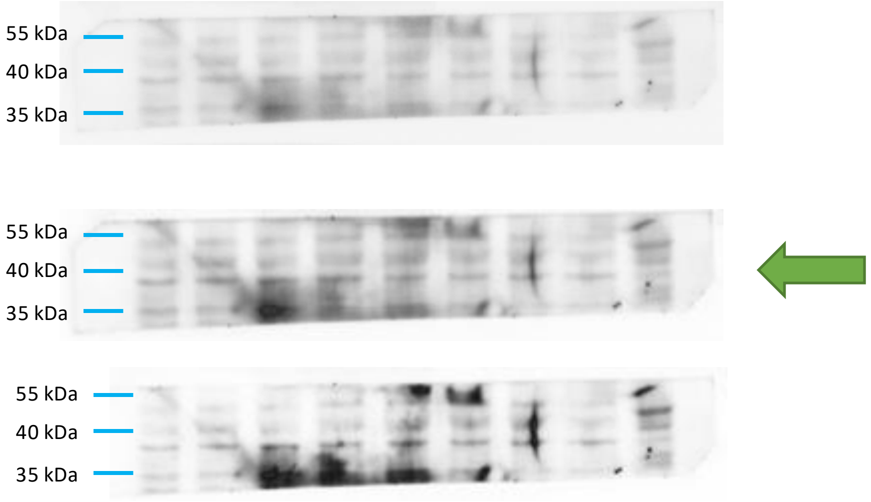

not used

5.1

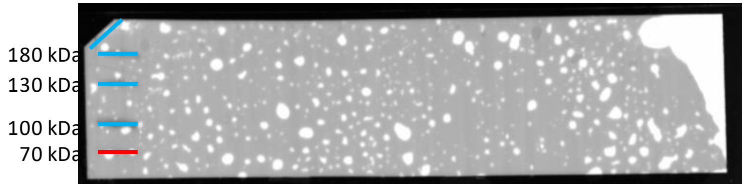

Used for Figure 7B, 8B, 10B

5.2

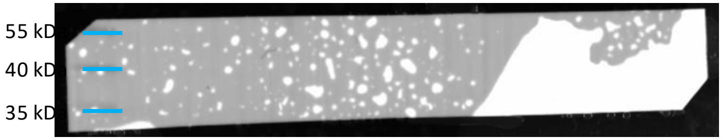

Raw WB MIA PaCa-2

gel electrophoresis

Gel 6

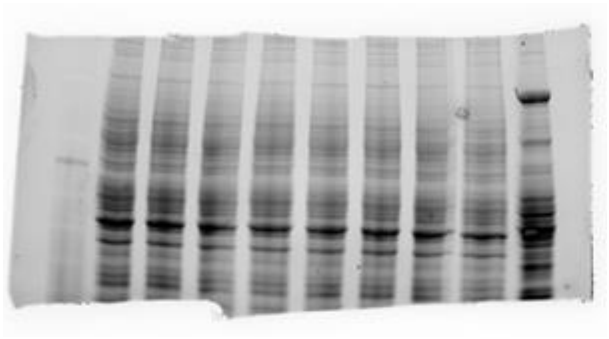

post-transfer membrane

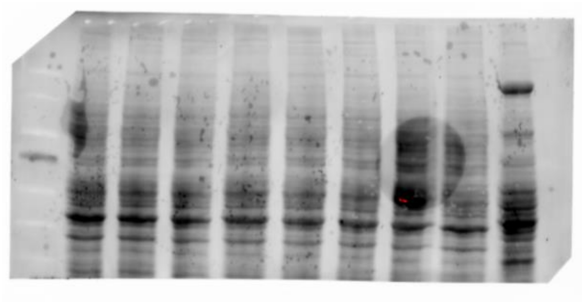

post-transfer membrane

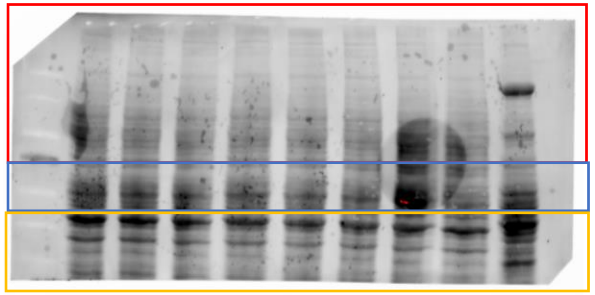

ChemiDoc image of the post-transfer membrane

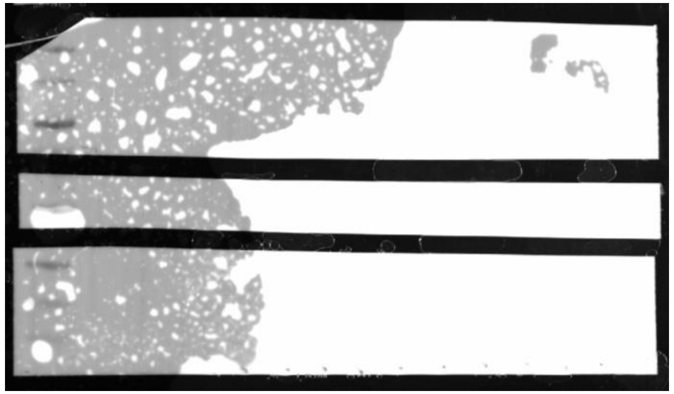

Abbreviations: Liver, mouse liver tissue (positive control), DOX, doxorubicin, MY, yellow maca, MR, red maca, MB, black maca,.

Raw WB MIA PaCa-2

6.1 PI3K
anti-rabbit

Dox | Dox +MY | Dox +MR | Dox +MB | liver

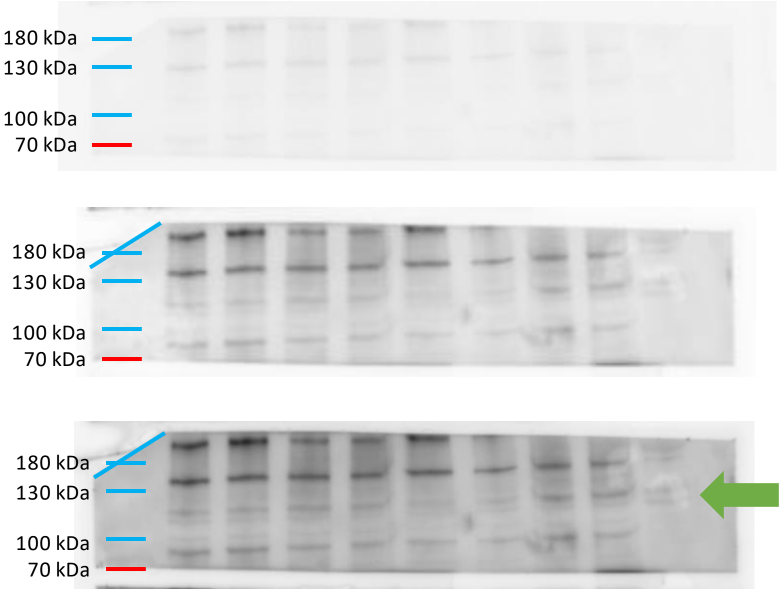

Used for Figure 8B

6.2 not used

Dox | Dox +MY | Dox +MR | Dox +MB | liver

6.3 not used

Dox | Dox +MY | Dox +MR | Dox +MB | liver

6.1

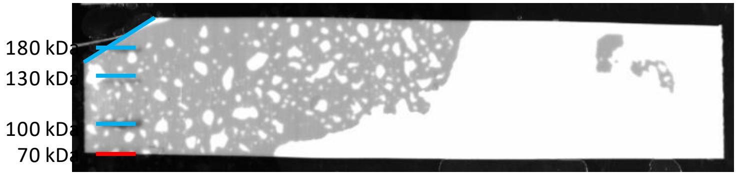

6.2

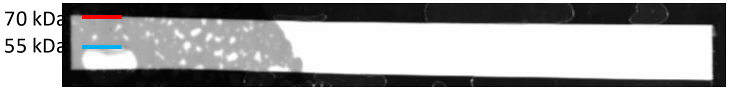

6.3

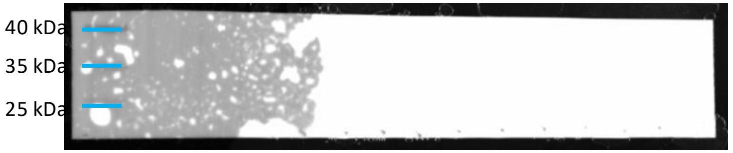

Raw WB MIA PaCa-2

gel electrophoresis

Gel 7

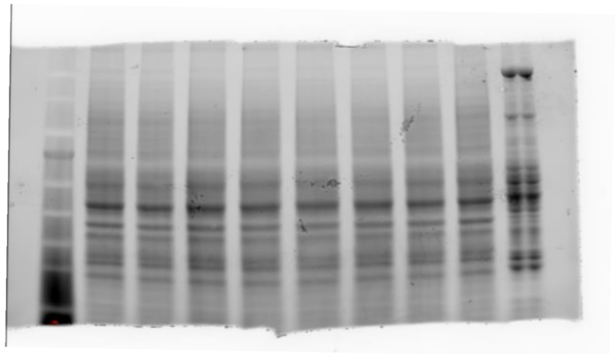

post-transfer membrane

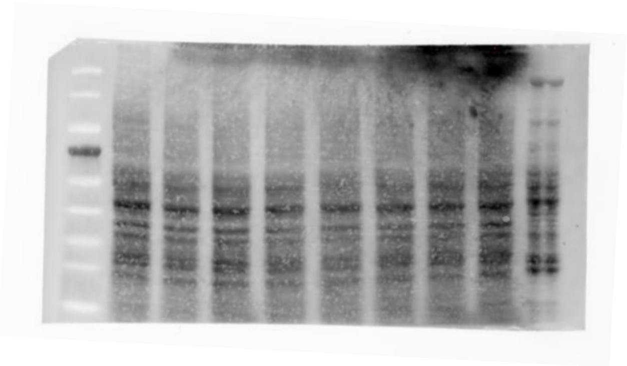

post-transfer membrane

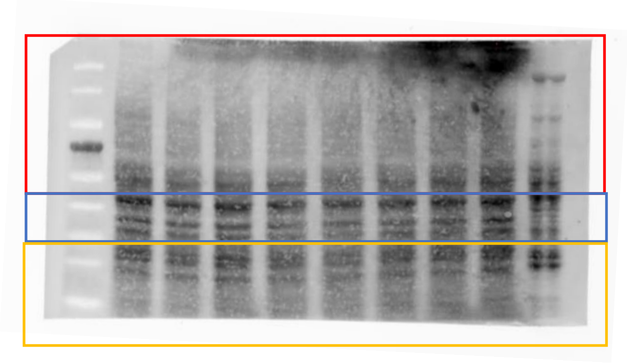

ChemiDoc image of the post-transfer membrane

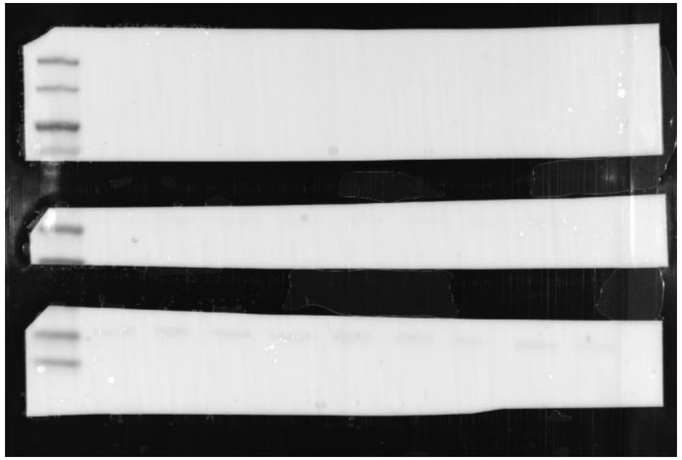

Abbreviations: Liver, mouse liver tissue (positive control), DOX, doxorubicin, MY, yellow maca, MR, red maca, MB, black maca,.

Raw WB MIA PaCa-2

7.1 FAP
anti-rabbit

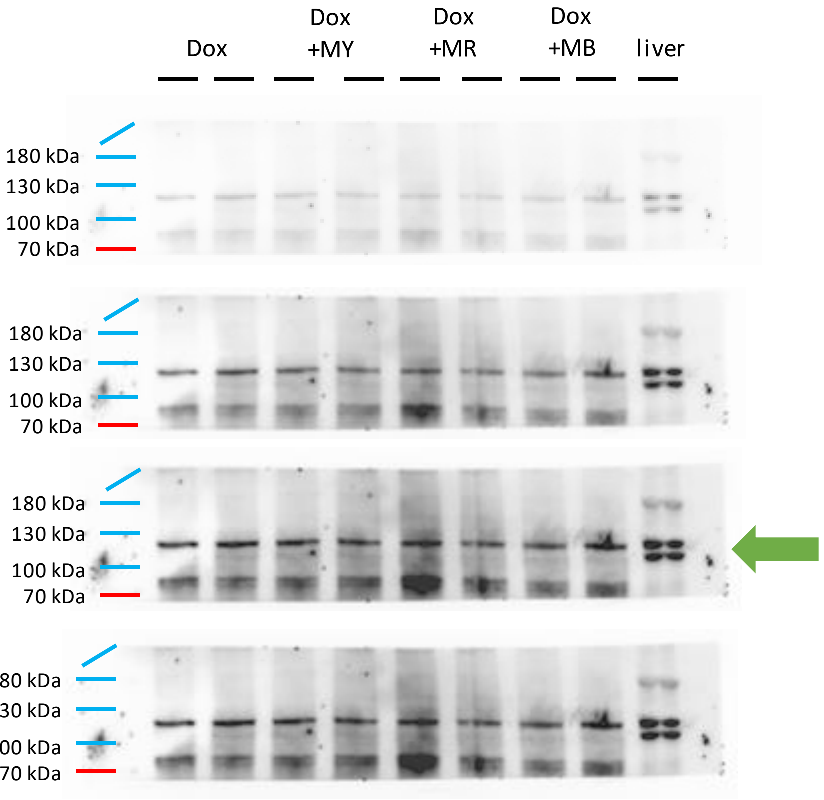

Used for Figure 8B

7.2 ATGL
anti-rabbit

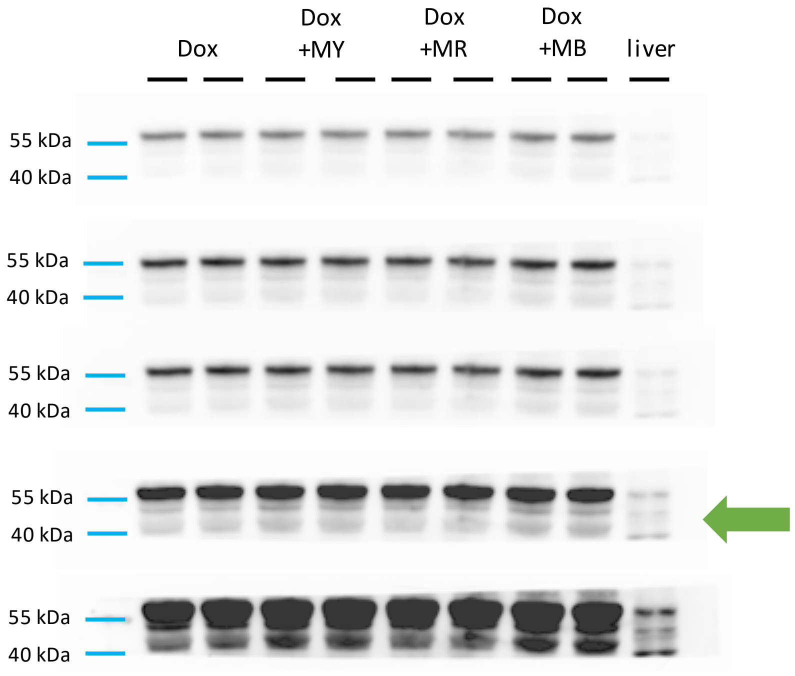

Used for Figure 10B

7.3 CASP3
anti-rabbit

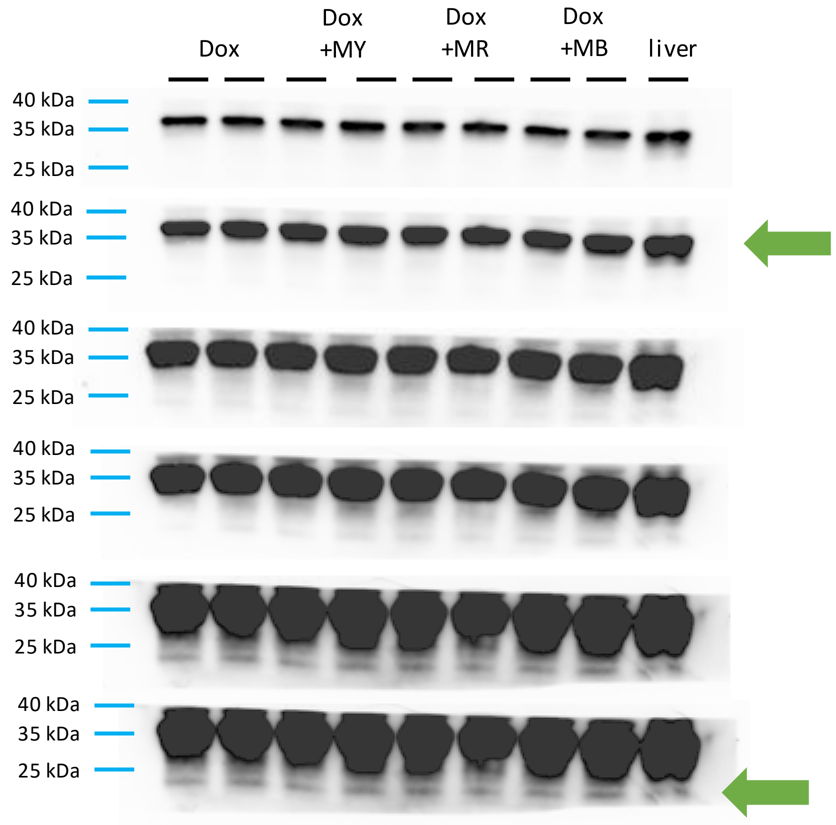

Used for Figure 7B

7.1

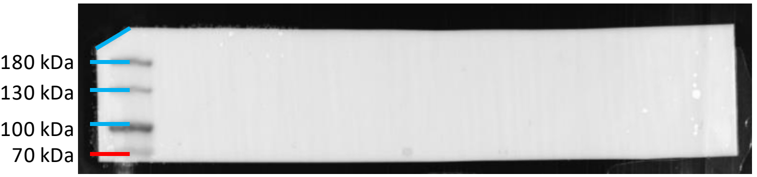

7.2

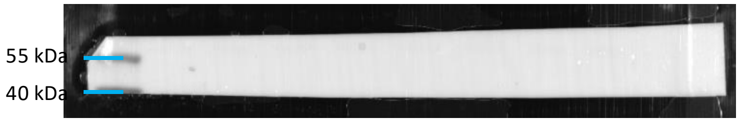

7.3

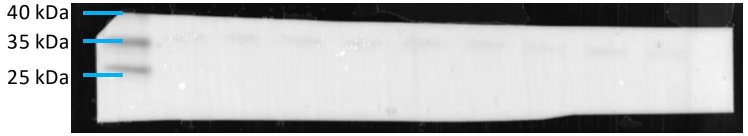

Raw WB MIA PaCa-2

gel electrophoresis

Gel 8

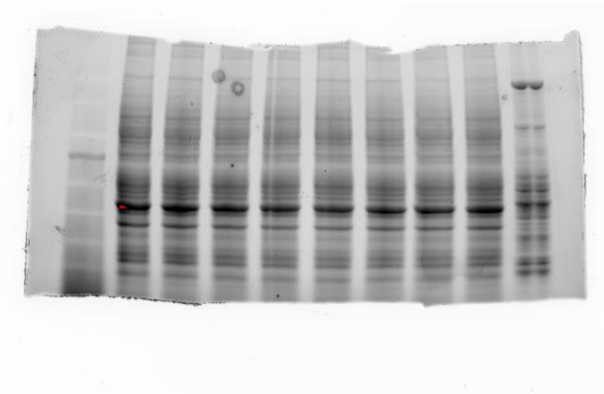

post-transfer membrane

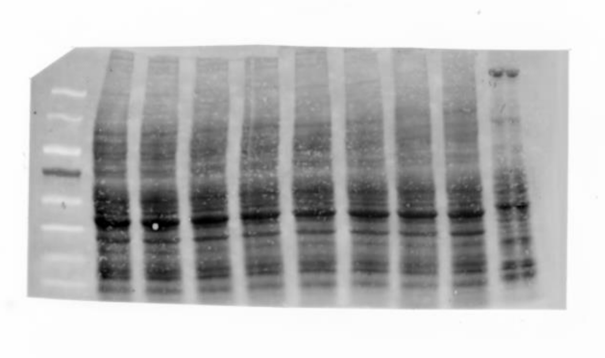

post-transfer membrane

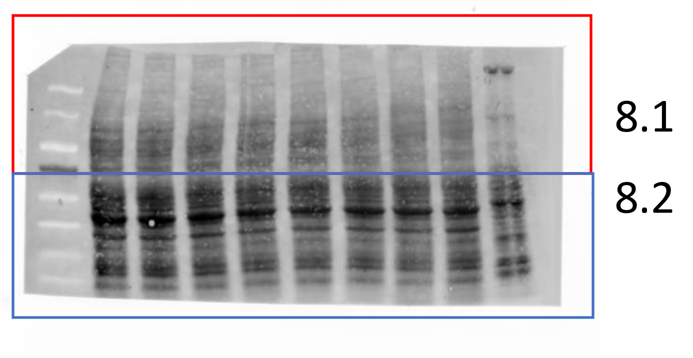

ChemiDoc image of the post-transfer membrane

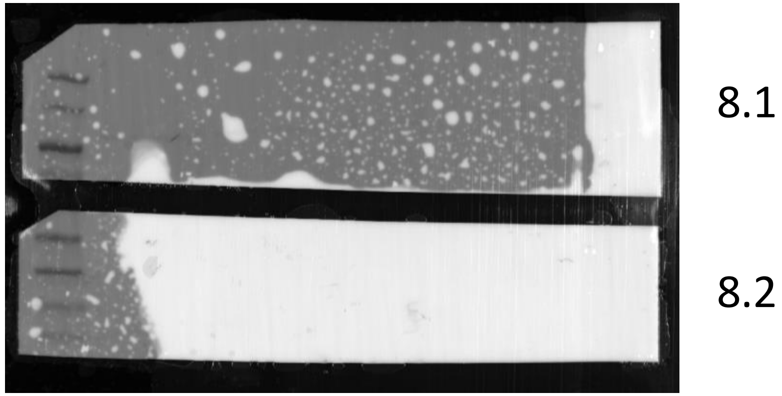

Abbreviations: Liver, mouse liver tissue (positive control), DOX, doxorubicin, MY, yellow maca, MR, red maca, MB, black maca,.

Raw WB MIA PaCa-2

8.1 FOXO1
anti-rabbit

Dox | Dox +MY | Dox +MR | Dox +MB | liver

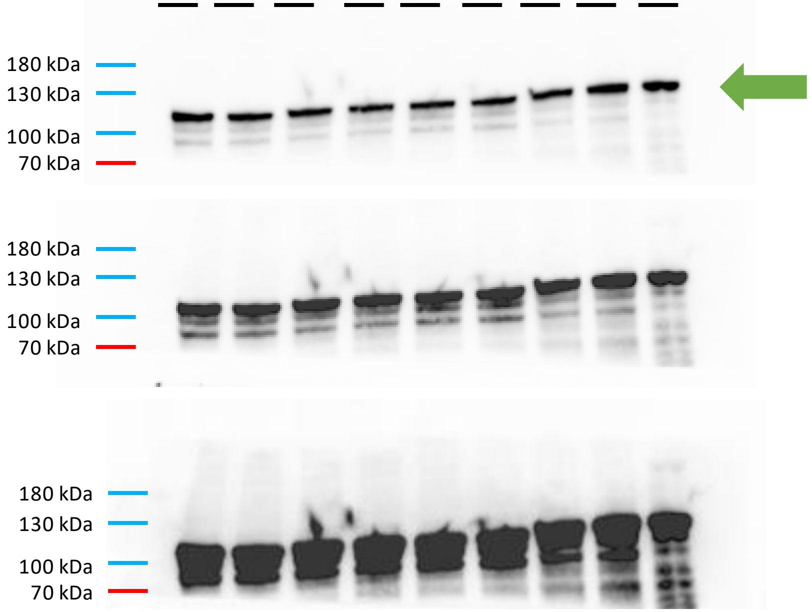

8.2 not used

Dox | Dox +MY | Dox +MR | Dox +MB | liver

Used for Figure 8B

8.1

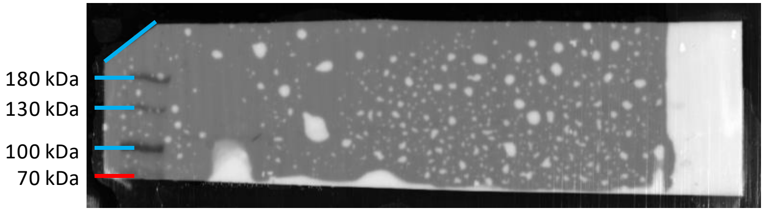

Not used

8.2

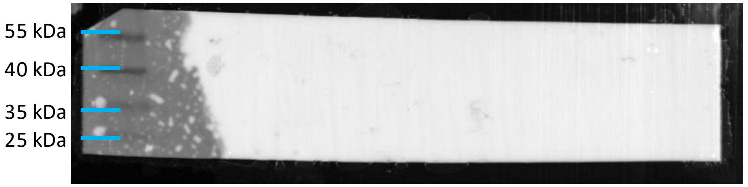

Raw WB MIA PaCa-2

gel electrophoresis

Gel 9

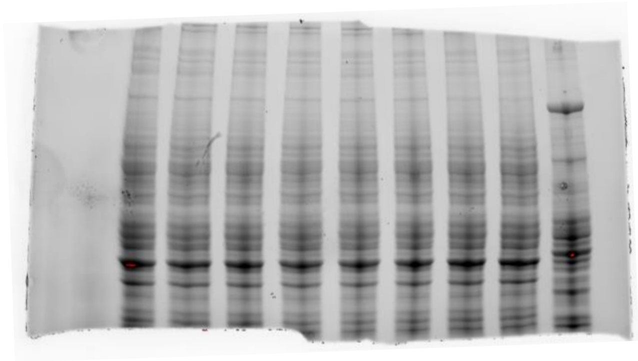

post-transfer membrane

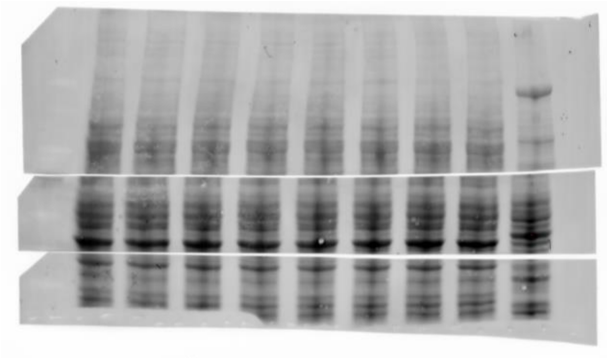

post-transfer membrane

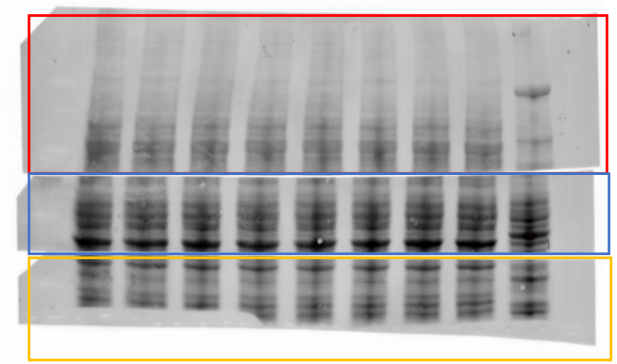

ChemiDoc image of the post-transfer membrane

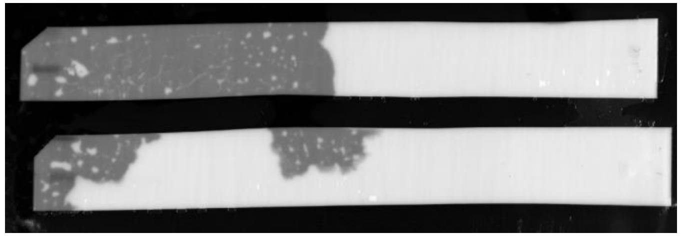

Abbreviations: Liver, mouse liver tissue (positive control), DOX, doxorubicin, MY, yellow maca, MR, red maca, MB, black maca,.

Raw WB MIA PaCa-2

9.1 not used

Dox | Dox +MY | Dox +MR | Dox +MB | liver

9.2 PLIN3
anti-mouse

Dox | Dox +MY | Dox +MR | Dox +MB | liver

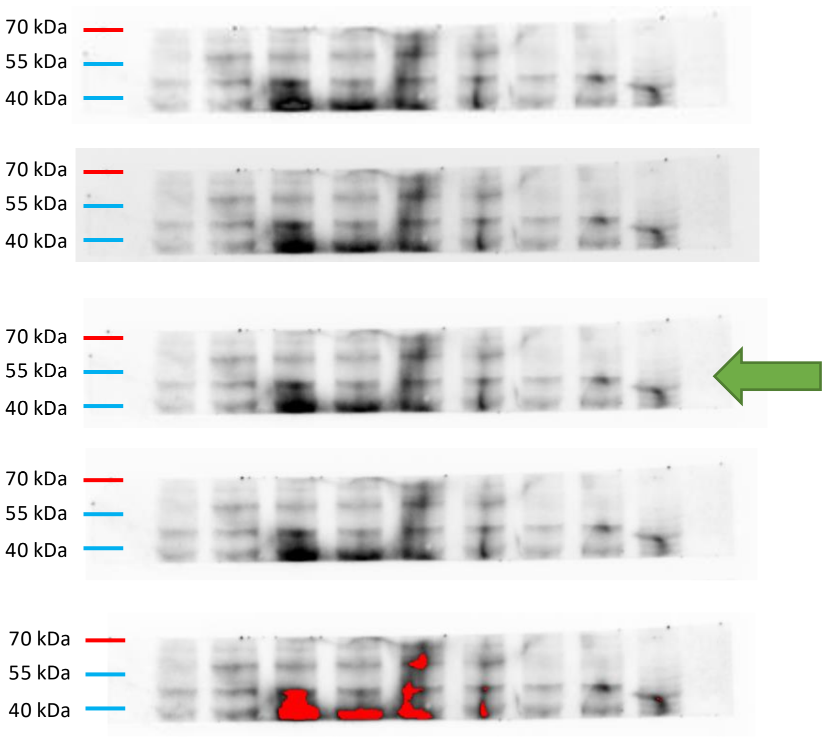

Used for Figure 10B

9.3 ABHD5
anti-mouse

Dox | Dox +MY | Dox +MR | Dox +MB | liver

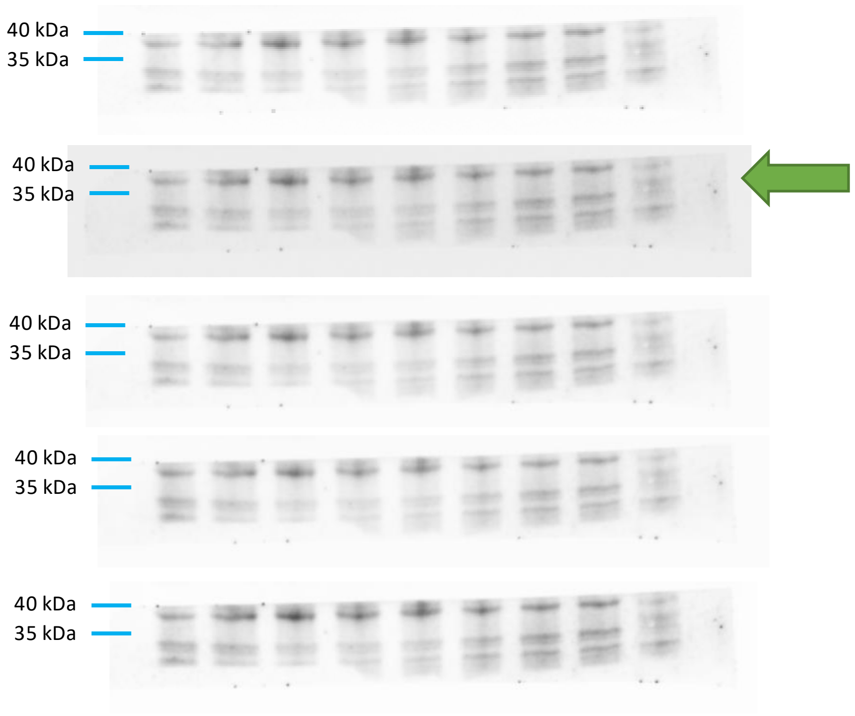

Used for Figure 10B

9.2

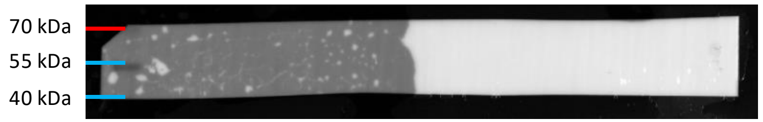

9.3

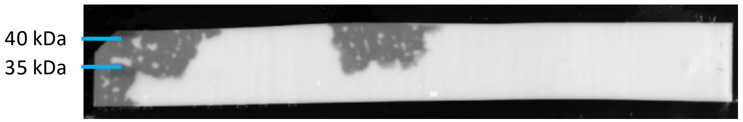

Raw WB PANC-1

gel electrophoresis

Gel 10

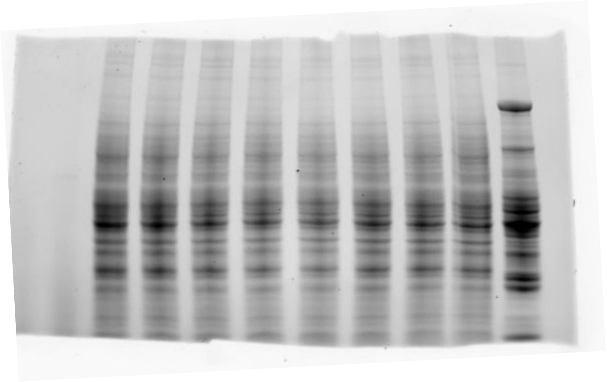

post-transfer membrane

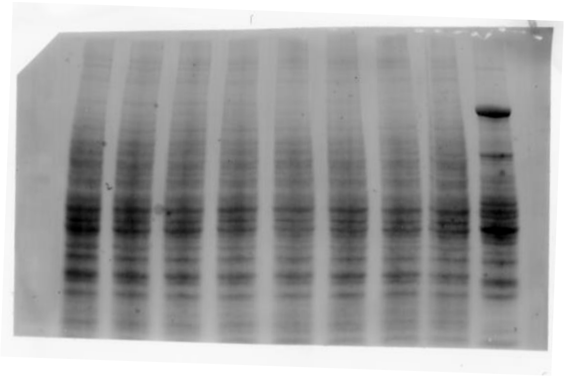

post-transfer membrane

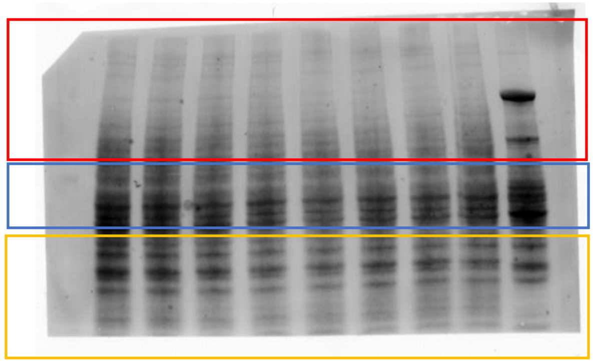

10.1

10.2

10.3

ChemiDoc image of the post-transfer membrane

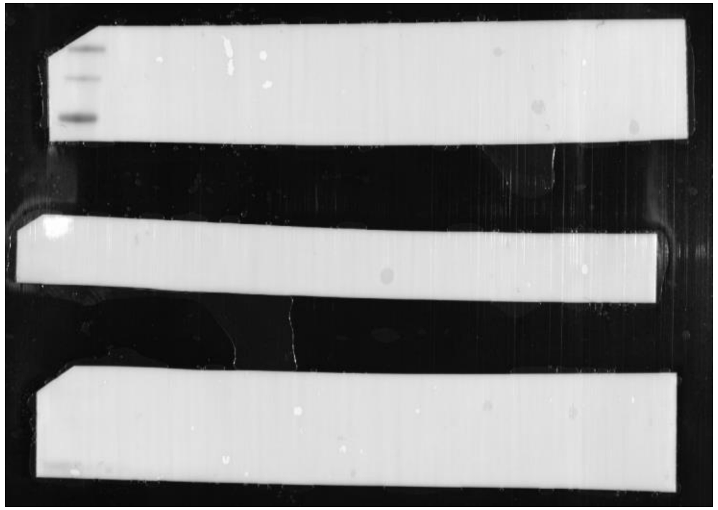

10.1

10.2

10.3

Abbreviations: Liver, mouse liver tissue (positive control), DOX, doxorubicin, MY, yellow maca, MR, red maca, MB, black maca,.

Raw WB PANC-1

10.1 FAP
anti-rabbit

Dox | Dox +MY | Dox +MR | Dox +MB | liver

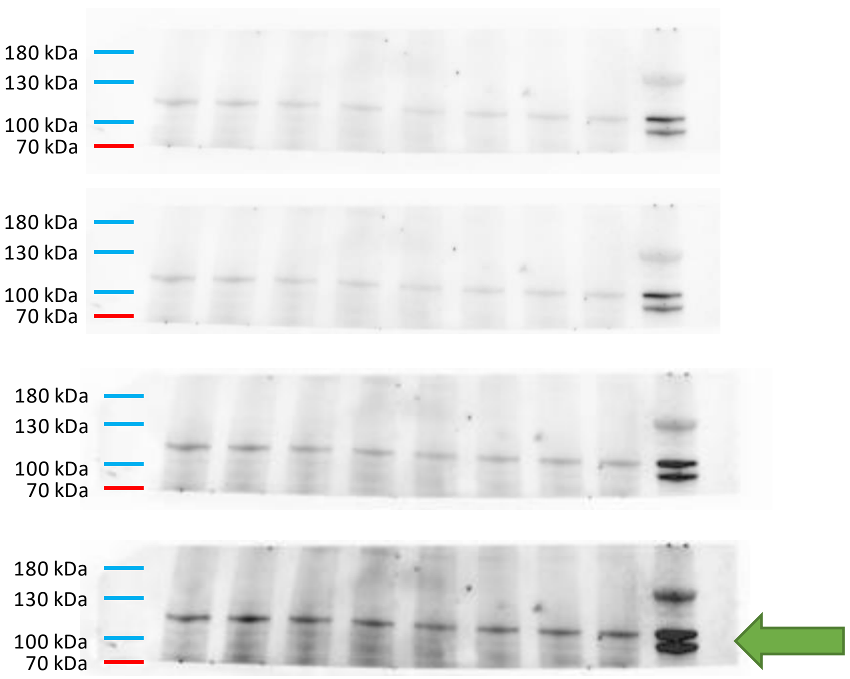

Used for Figure 8C

10.1

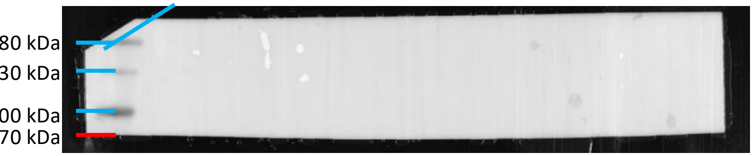

10.2 not used

Dox | Dox +MY | Dox +MR | Dox +MB | liver

not used

10.2

10.3 ABHD5
anti-mouse

Dox | Dox +MY | Dox +MR | Dox +MB | liver

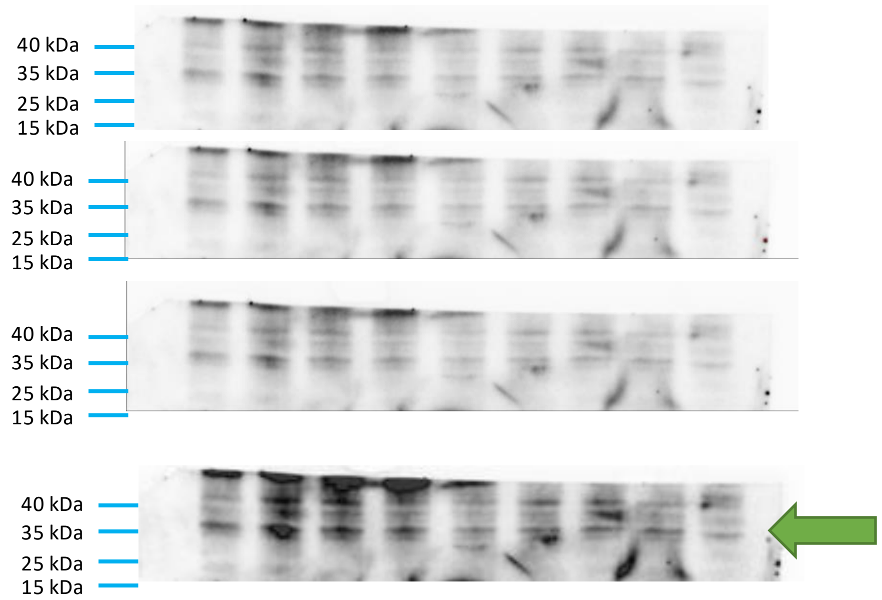

Used for Figure 10C

10.3

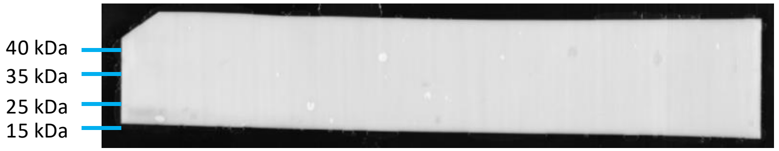

Raw WB PANC-1

gel electrophoresis

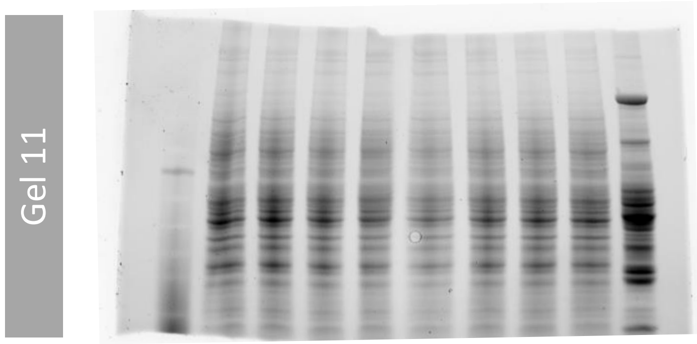

post-transfer membrane

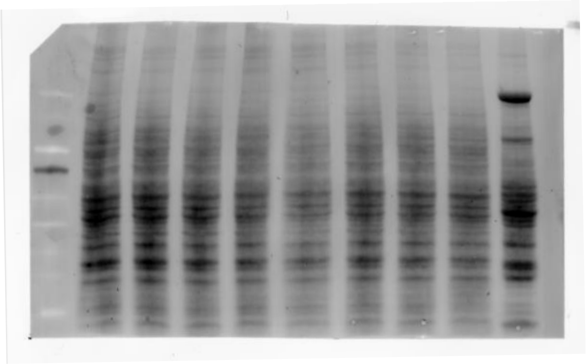

post-transfer membrane

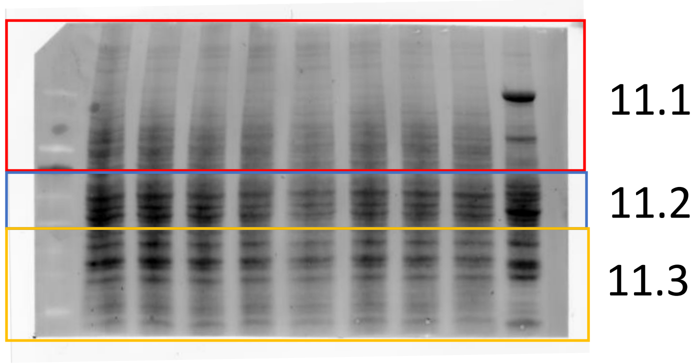

ChemiDoc image of the post-transfer membrane

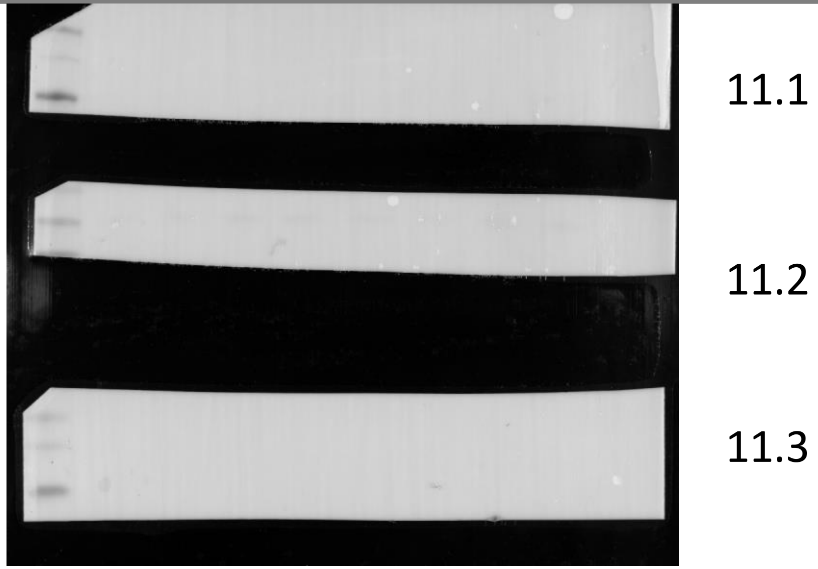

Abbreviations: Liver, mouse liver tissue (positive control), DOX, doxorubicin, MY, yellow maca, MR, red maca, MB, black maca,.

Raw WB PANC-1

11.1 PI3K
anti-rabbit

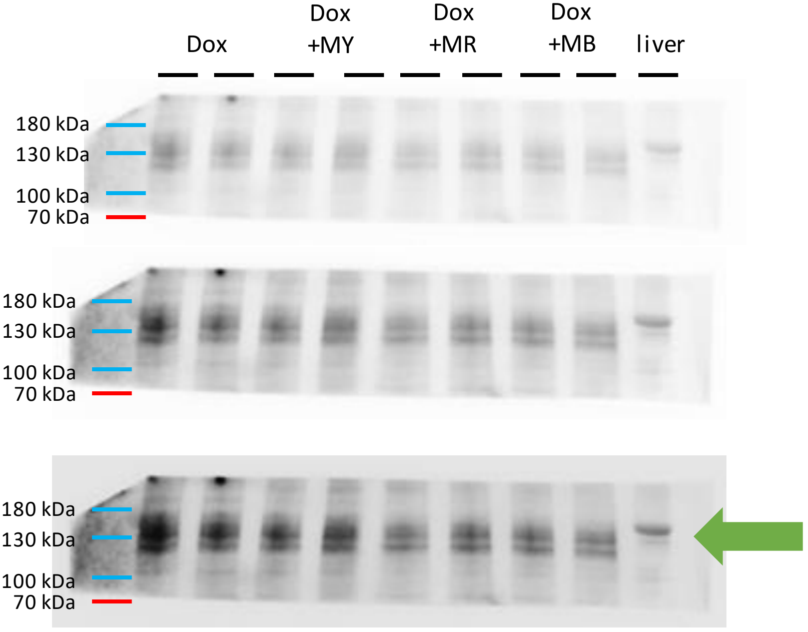

11.2 ATGL
anti-rabbit

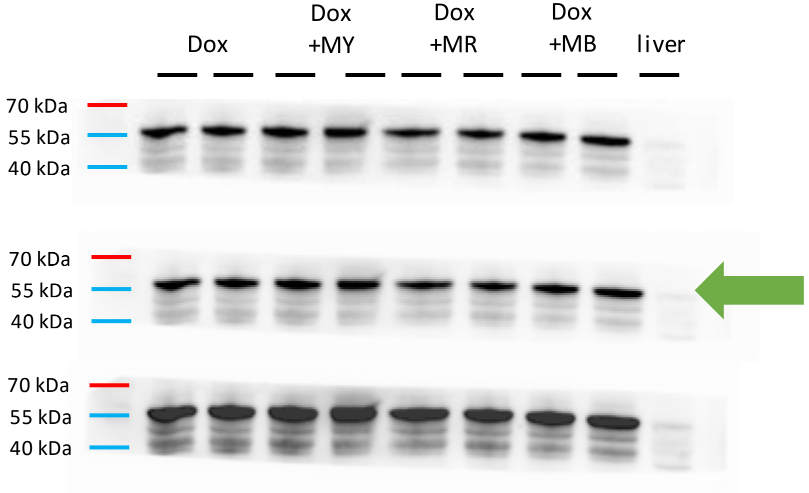

11.3 GAPDH
anti-mouse

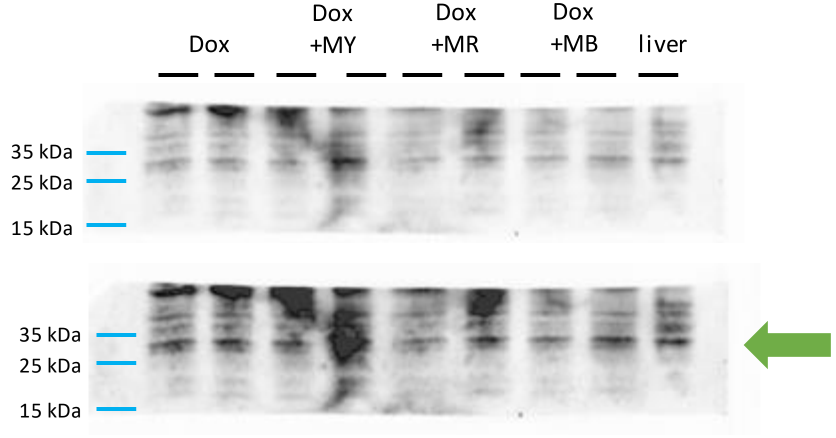

Used for Figure 8C

Used For Figure 10C

Used for Figure 7C, 8C AND 10C

11.1

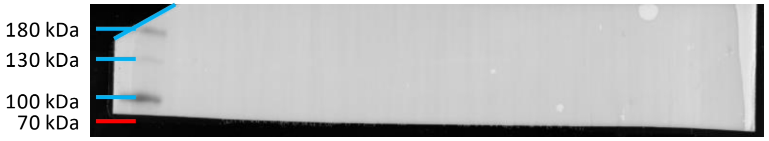

11.2

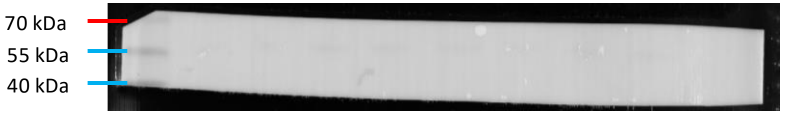

11.3

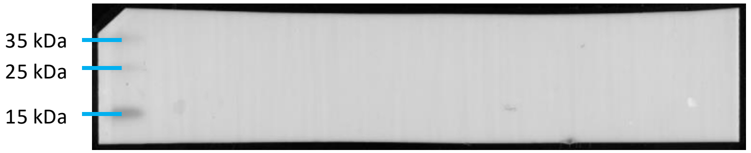

Raw WB PANC-1

gel electrophoresis

Gel 12

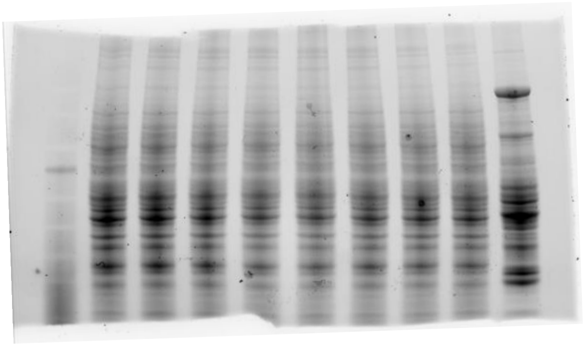

post-transfer membrane

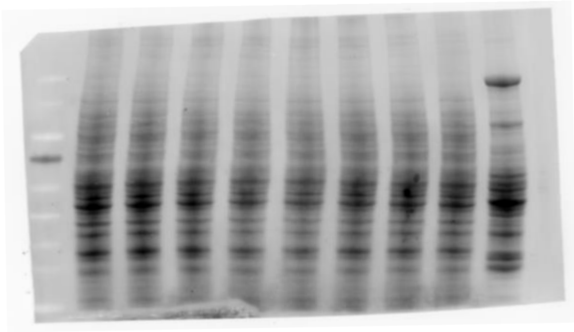

post-transfer membrane

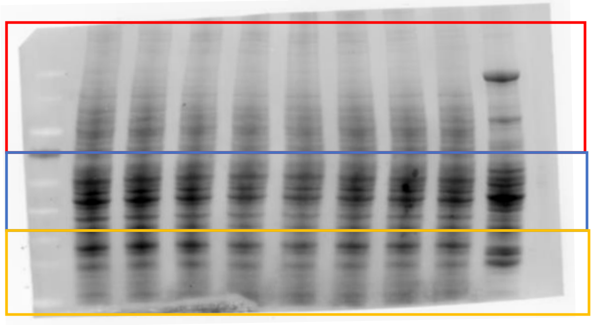

12.1

12.2

12.3

ChemiDoc image of the post-transfer membrane

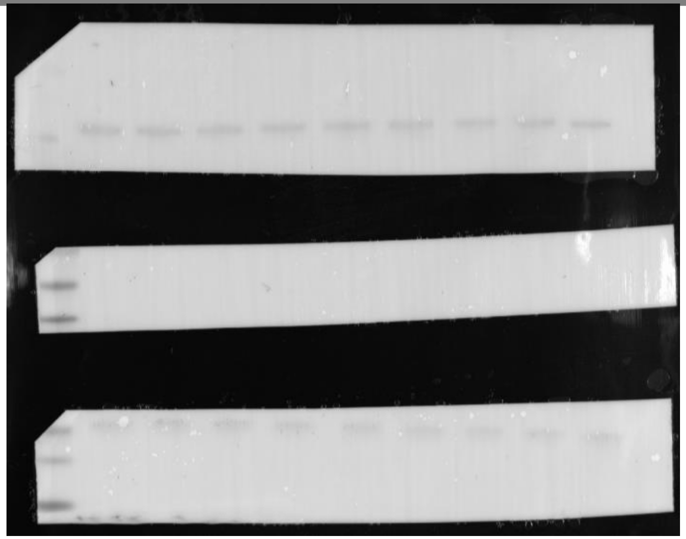

12.1

12.2

12.3

Abbreviations: Liver, mouse liver tissue (positive control), DOX, doxorubicin, MY, yellow maca, MR, red maca, MB, black maca,.

Raw WB PANC-1

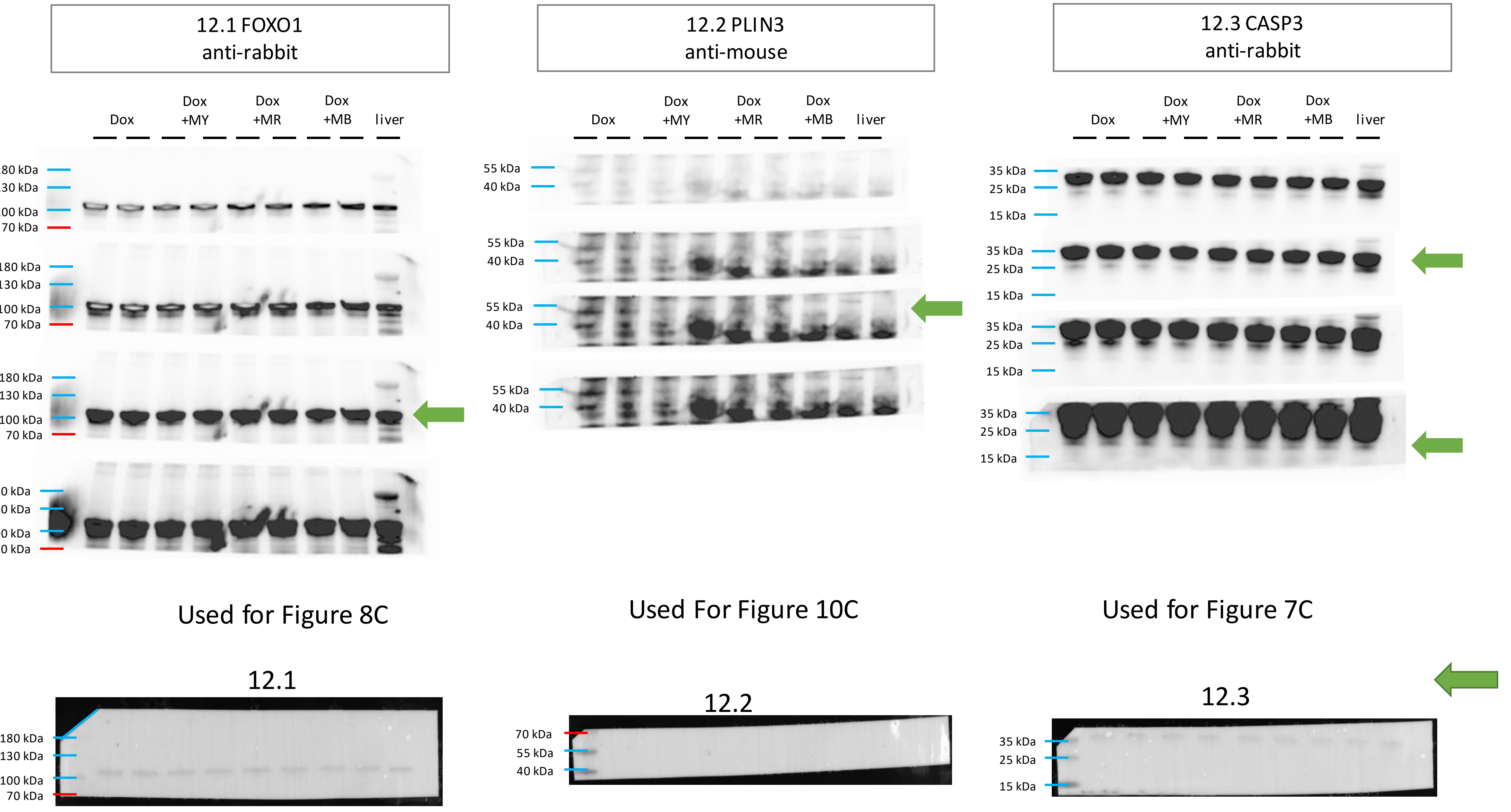

12.1 FOXO1
anti-rabbit
12.2 PLIN3
anti-mouse
12.3 CASP3
anti-rabbit
Dox
Dox +MY
Dox +MR
Dox +MB
liver
180 kDa
130 kDa
100 kDa
70 kDa
55 kDa
40 kDa
35 kDa
25 kDa
15 kDa
Used for Figure 8C
Used For Figure 10C
Used for Figure 7C
12.1
12.2
12.3

Western blot membrane images obtained after stripping and subsequent re-incubation with primary and secondary antibodies are presented. These images document the results of membrane stripping and reprobing procedures performed during the Western blot experiments included in this manuscript.

WB BxPc-3 after stripping and re-incubated with GAPDH antibody

2.3 ABHD5→GAPDH
anti-mouse

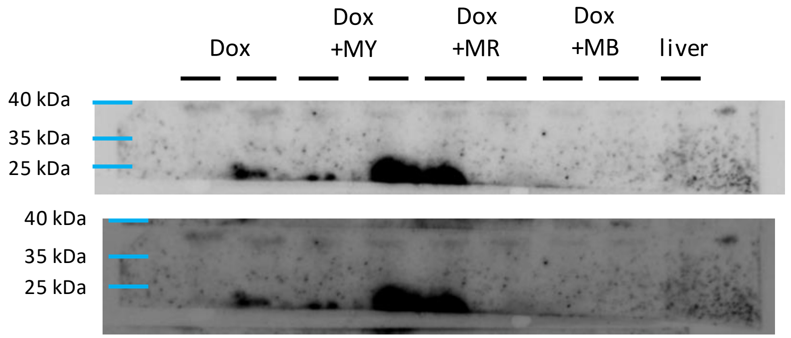

2.3 CASP3 →GAPDH
anti-mouse

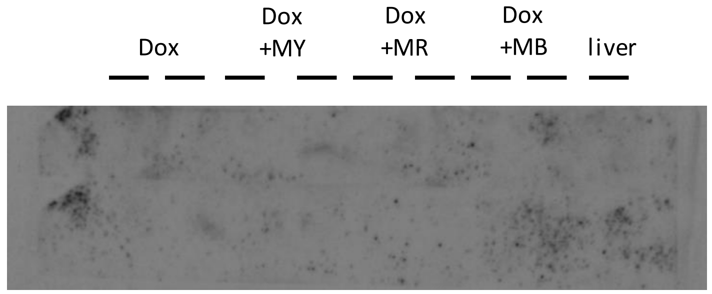

4.2 PLIN3 →GAPDH
anti-mouse

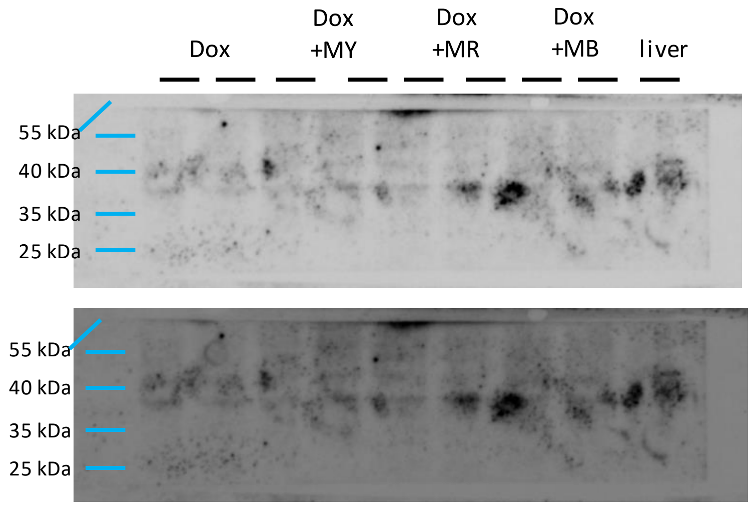

2.3

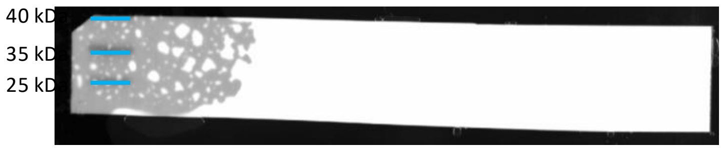

3.3

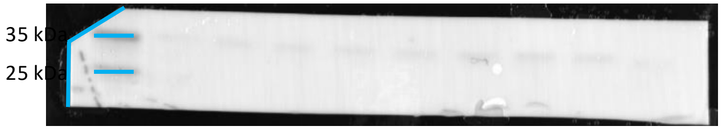

4.2

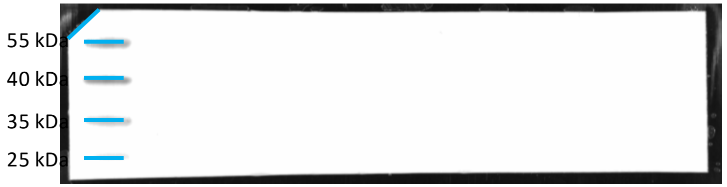

WB MIA PaCa-2 after stripping and re-incubated with GAPDH antibody

6.3 ABHD5 →GAPDH
anti-mouse

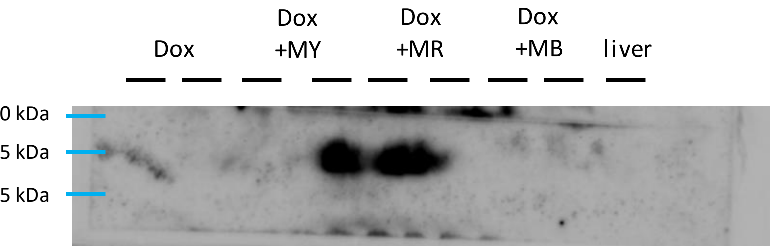

6.3

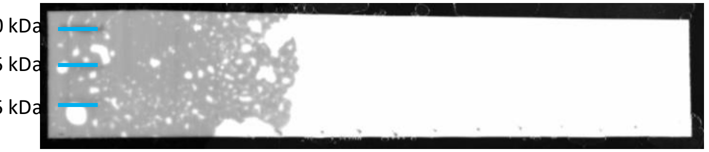

7.3 CASP3 →GAPDH
anti-mouse

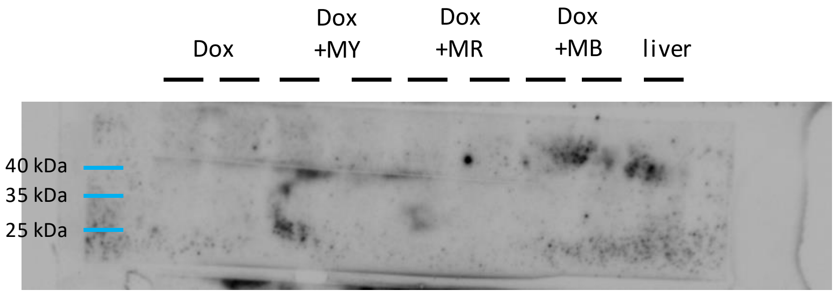

7.3

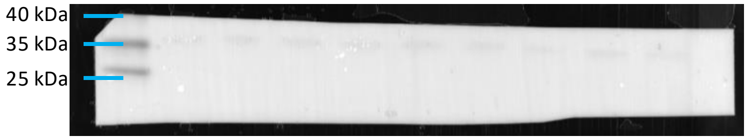

8.2 PLIN3 →GAPDH
anti-mouse

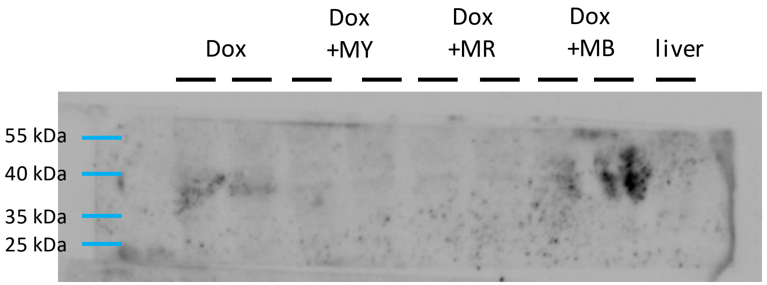

8.2

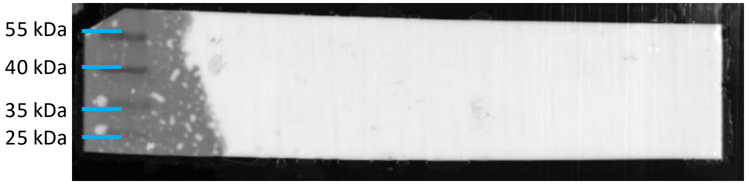

9.3 ABHD5 →GAPDH
anti-mouse

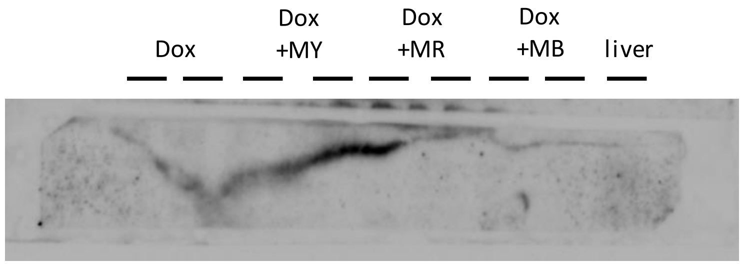

9.3

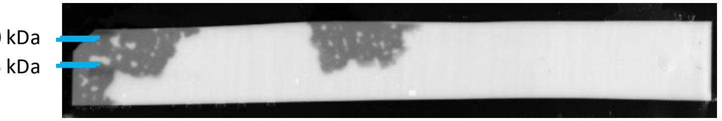

WB PANC-1 after stripping and re-incubated with GAPDH antibody

10.3 ABHD5 →GAPDH
anti-mouse

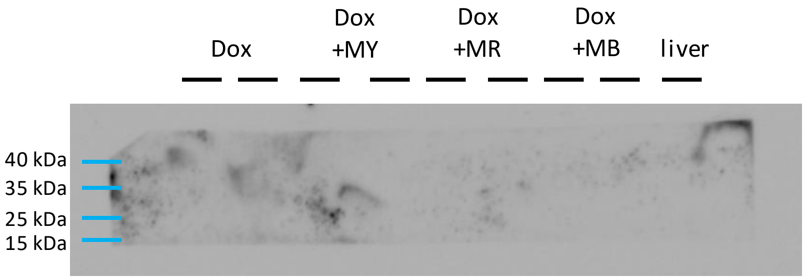

Used for Figure 10C

12.3 CASP3 →GAPDH
anti-mouse

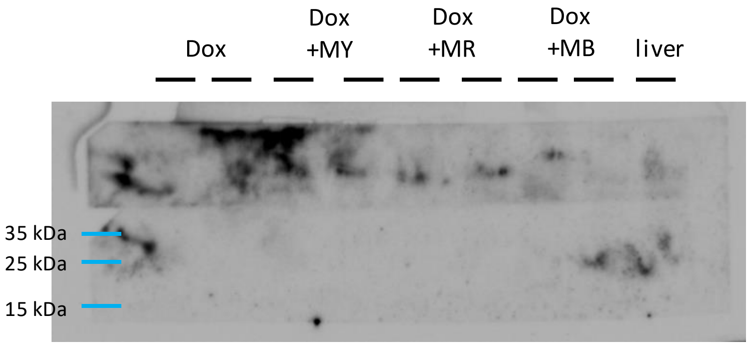

Used for Figure 7C

10.3

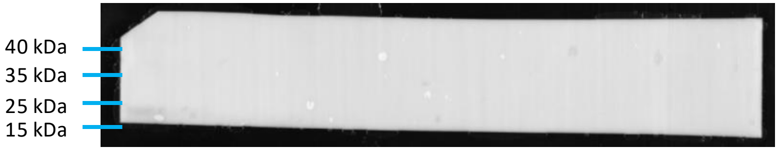

12.3

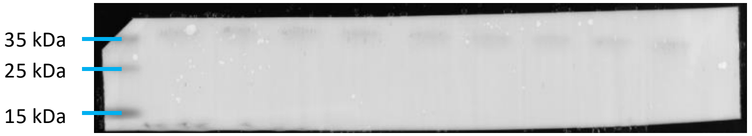

Supplement: Supplementary file 1 [file molecules-31-04011-s001.zip › Full-length uncropped Western blot images for all Western blot experiments.pdf]
