# Supplementary material for: Yellow, Red, and Black Lepidium meyenii Preparations Differentially Modulate Doxorubicin Response and Lipid Metabolism-Related Changes in Pancreatic Cancer Cells
Source: Molecules. 2026 Nov 6;31(21):4011. doi: 10.3390/molecules31214011 (PMC13765015; doi:10.3390/molecules31214011)
Supplement: Supplementary file 1 [file molecules-31-04011-s001.zip › MACA_BLACK Gamma SDectrosc0DV Analvsis Report.pdf]

LVis - Gamma Spectroscopy Analysis Report

**Name:**	**MACA_BLACK.lvm**<br>*C:\LVis\Measurements\GAMMA 1\MACA\MACA_BLACK.LVM*		
**Detector:**	**GAMMA 1**		
**Counting Routine:**	**cylinder_sample**		
**Batch ID:**	**MACA_BLACK**		
**Generator ID:**	**MACA_BLACK**		
**Category:**	**MACA_BLACK**	**User:**	LVis Admin (D)
**Start:**	24.09.2025 12:31:38	**Analyzed:**	16.03.2026 10:32:46
**Reference date/time:**	-	**Age of Sample:**	-
**Live Time:**	177607 s	**Total Countrate:**	3.75 counts/s
**Real Time:**	177739 s	**Dead Time:**	0.07 %
**Sample mass:**	50.50 g	**spec. Activity in:**	Bq/kg
**Sample volume:**	2.00 cm	**Reference:**	1.00
**Activity Scaling Factor:**	1,9802E+01	**Add. Uncertainties:**	0.00 %

Analysis Report according to ISO 11929

Nuclide	Detection Limit [Bq/kg]	Decision Threshold [Bq/kg]	Confidence Limits [Bq/kg]		Best Estimate corrected [Bq/kg]	1-σ Total Uncert.	
			lower	upper		[Bq/kg]	%
**K-40**	2.3303E+01	1.1557E+01	3.5603E+02	3.972E+02	3.7662E+02	1.0488E+01	2.78
**Th-234**	1.5166E+01	7.5155E+00					
**Pa-234m**	1.1488E+02	5.6626E+01					
**Th-230**	5.395E+01	2.643E+01					
**Ra-226**	1,5978E+01	7,9347E+00					
**Pb-214**	1,9796E+00	9,8112E-01					
**Bi-214**	7.5739E+00	3.7132E+00					
**Pb-210**	2,9092E+01	1,4444E+01					
**U-235D**	8.4149E+00	4.1744E+00					
**Th-227**	2.7551E+00	1.3602E+00					
**Ra-223**	3,5158E+00	1,7406E+00					
**Rn-219**	1.6914E+00	8.2431E-01					
**Pb-211**	1.4437E+01	7.1241E+00					
**Bi-211**	5,6767E+00	2,8143E+00					
**Ac-228**	2.532E+00	1.2412E+00					
**Ra-224**	1,3067E+01	6,478E+00					
**Pb-212**	1.3671E+00	6,7832E-01					
**Bi-212**	1.0149E+01	4.9919E+00					
**Tl-208**	1,0145E+00	5,0112E-01					
**Cs-137**	6.8822E-01	3.383E-01					
**Annihil**	1.2436E+00	6.1906E-01					

H = Halflife limit exceeded

Sum [Bq/kg]: 3,7662E+02 +/- 1,0488E+01

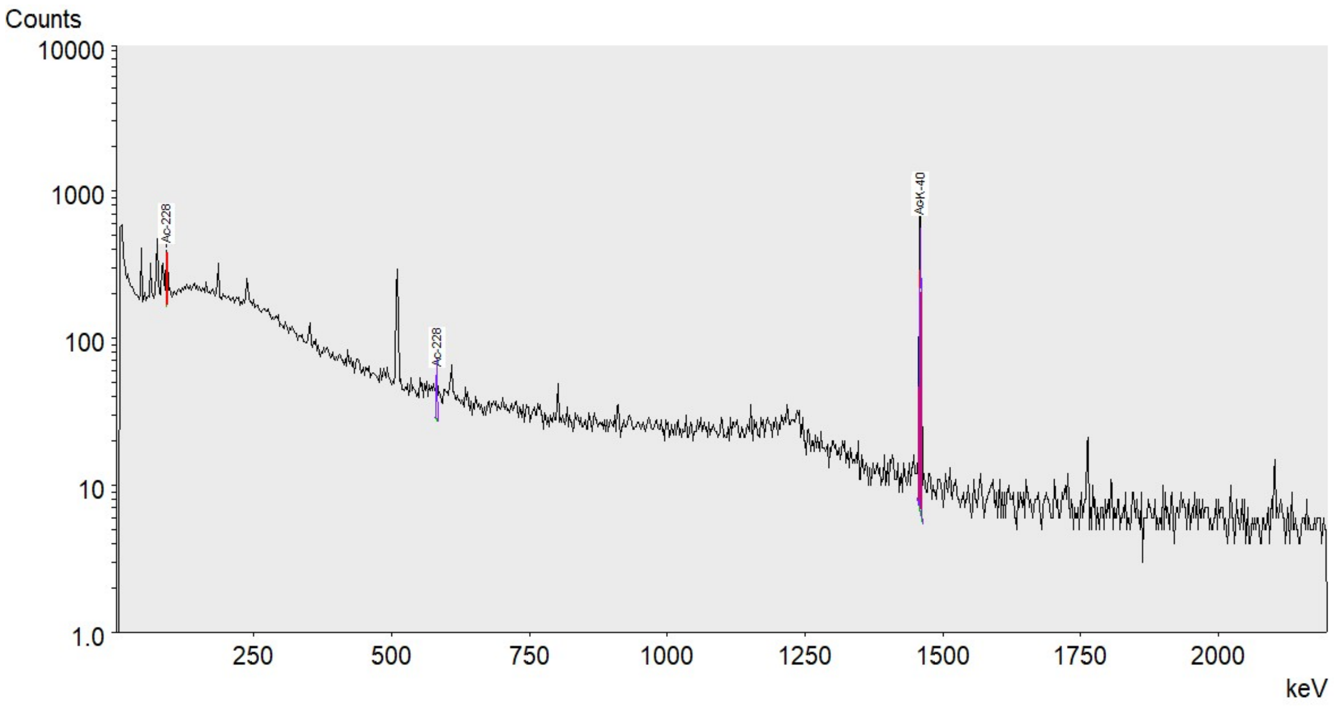

All analysis peaks

Energy [keV]	FWHM [keV]	FW10M [keV]	Gross Area [counts]	Net Area [counts]	Net - PBC [counts]	Intensity [cps]	1-σ Uncert. [%]	Nuclide	Efficiency [%]	Info
57.84	1.16	1.30	1,184	97	97	5,449E-04	45.57	Ac-228	4.26	s
72.81	0.73	1.39	4,304	677	100	5,658E-04	105.5	Tl-208	5.73	P
77.24	0.48	1.14	4,034	720	382	2,151E-03	26.60	Ac-228	6.01	s
84.78	0.94	1.73	6,570	1,210	134	7,544E-04	103.4	Tl-208	6.33	P s
90.03	0.40	1.83	3,732	294	294	1,657E-03	35.56	Ac-228	6.46	s
**92.64**	**1.03**	**1.80**	**5,729**	**1,752**	**1,752**	**9,866E-03**	**7.05**	**Ac-228**	**6.53**	**s**
100.56	0.13	0.23	1,221	19	19	1,086E-04	235.4	Ac-228	6.59	s
105.96	1.20	1.42	1,351	105	105	5,936E-04	44.72	Ac-228	6.59	s
108.48	0.47	0.60	1,290	2	2	8,817E-06	2,990	Ac-228	6.58	s
128.91	0.20	1.06	1,423	108	108	6,091E-04	44.75	Ac-228	6.33	s
135.40	0.36	0.51	1,459	122	122	6,846E-04	40.25	Ac-228	6.22	s
153.17	1.10	1.51	1,410	99	99	5,572E-04	48.75	Ac-228	5.85	s
168.29	0.65	1.48	1,342	140	140	7,888E-04	33.35	Ac-228	5.56	s
173.98	2.47	2.57	1,323	20	20	1,151E-04	231.3	Ac-228	5.45	s
191.00	1.66	1.83	1,213	18	18	1,006E-04	253.3	Ac-228	5.12	s
204.26	0.25	0.37	1,558	40	40	2,268E-04	128.9	Ac-228	4.90	s
211.36	0.64	0.82	1,543	96	96	5,426E-04	53.20	Tl-208	4.77	s
224.12	0.20	0.60	1,455	54	54	3,023E-04	93.25	Ac-228	4.58	s
231.27	0.19	1.71	1,490	142	142	7,991E-04	35.24	Ac-228	4.47	s
233.45	1.11	1.26	1,401	61	61	3,437E-04	80.34	Tl-208	4.44	s
257.89	2.37	2.56	1,376	16	16	9,25E-05	298.0	Ac-228	4.12	s
270.03	0.68	1.40	1,292	58	58	3,28E-04	80.83	Ac-228	3.98	s
332.31	2.23	2.34	840	73	73	4,124E-04	51.35	Ac-228	3.39	s
337.76	1.41	1.70	819	50	50	2,79E-04	75.40	Ac-228	3.35	s
356.64	0.49	0.58	678	6	6	3,389E-05	571.2	Ac-228	3.22	s
377.89	0.37	0.47	639	25	25	1,42E-04	131.6	Ac-228	3.08	s
416.30	0.95	1.04	532	31	31	1,754E-04	93.82	Ac-228	2.87	
471.43	1.52	1.61	498	16	16	9,241E-05	180.4	Ac-228	2.61	s
475.32	2.84	3.28	484	4	4	2,393E-05	690.1	Ac-228	2.60	s
486.26	0.29	0.47	510	31	31	1,727E-04	97.05	Tl-208	2.55	s
503.43	2.09	2.20	474	41	41	2,295E-04	70.06	Ac-228	2.49	s
510.36	1.35	2.06	10,042	4,571	362	2,041E-03	44.29	Tl-208	2.46	s
519.79	1.13	1.27	436	39	39	2,185E-04	70.50	Ac-228	2.43	
523.24	1.30	1.42	406	7	7	3,832E-05	394.1	Ac-228	2.42	
540.63	0.13	0.24	419	41	41	2,308E-04	65.28	Ac-228	2.36	s
572.65	0.16	0.44	419	76	76	4,283E-04	34.48	Ac-228	2.27	s
**582.78**	**1.75**	**2.30**	**1,811**	**613**	**323**	**1,819E-03**	**22.32**	**Ac-228**	**2.24**	**s**
586.19	0.51	0.62	1,176	182	182	1,022E-03	34.61	Tl-208	2.23	s

All analysis peaks

Energy [keV]	FWHM [keV]	FW10M [keV]	Gross Area [counts]	Net Area [counts]	Net - PBC [counts]	Intensity [cps]	1-σ Uncert. [%]	Nuclide	Efficiency [%]	Info
629.08	1.20	1.29	344	35	35	1,984E-04	66.91	Ac-228	2.12	
639.37	1.05	1.25	305	8	8	4,755E-05	267.3	Ac-228	2.10	
648.63	0.61	0.76	319	60	60	3,393E-04	37.02	Ac-228	2.08	s
651.81	1.36	1.68	327	62	62	3,498E-04	36.30	Ac-228	2.07	
697.87	1.42	1.60	288	7	7	4,022E-05	307.2	Ac-228	1.97	
705.95	0.15	0.33	281	21	21	1,187E-04	101.7	Tl-208	1.96	s
722.85	1.68	1.78	258	2	2	1,089E-05	1,078	Tl-208	1.92	s
727.37	0.52	1.08	331	68	68	3,817E-04	33.38	Ac-228	1.92	s
755.53	0.51	0.66	308	27	27	1,533E-04	84.95	Ac-228	1.86	s
762.51	1.23	1.27	278	5	5	2,595E-05	485.0	Tl-208	1.85	
795.15	0.13	0.24	600	73	73	4,124E-04	53.26	Ac-228	1.80	s
808.79	0.93	1.39	719	80	80	4,504E-04	58.05	Tl-208	1.78	s
824.95	0.19	0.44	255	3	3	1,522E-05	793.9	Ac-228	1.75	s
839.12	2.67	2.88	296	43	43	2,412E-04	52.33	Ac-228	1.73	s
853.89	1.68	2.03	254	35	35	1,964E-04	59.63	Ac-228	1.71	
859.35	1.79	2.42	977	246	162	9,121E-04	31.71	Tl-208	1.70	s
873.07	0.00	-1.00	245	14	14	7,916E-05	144.4	Ac-228	1.68	s
874.93	0.14	0.45	244	11	11	6,291E-05	181.6	Ac-228	1.68	s
886.96	0.36	0.92	253	16	16	8,945E-05	129.8	Ac-228	1.66	s
901.24	0.00	-1.00	236	7	7	4,059E-05	278.0	Ac-228	1.64	s
904.85	0.34	0.51	715	68	68	3,801E-04	67.01	Ac-228	1.64	s
910.45	1.62	2.29	894	187	26	1,476E-04	183.7	Ac-228	1.63	P
922.19	3.32	3.37	267	44	44	2,481E-04	46.98	Ac-228	1.62	s
944.89	0.76	0.84	235	16	16	8,914E-05	125.2	Ac-228	1.59	s
964.30	0.64	0.82	262	29	29	1,614E-04	72.39	Ac-228	1.56	s
968.35	1.48	2.01	643	196	104	5,868E-04	33.22	Ac-228	1.56	
1,020.45	1.05	1.17	216	14	14	7,612E-05	140.9	Ac-228	1.50	s
1,040.90	0.26	0.38	234	40	40	2,265E-04	48.14	Ac-228	1.48	s
1,056.29	0.57	0.62	230	11	11	6,362E-05	174.4	Ac-228	1.46	s
1,061.76	0.16	0.30	281	32	32	1,803E-04	69.05	Ac-228	1.46	s
1,095.77	0.64	0.71	261	13	13	7,493E-05	162.4	Ac-228	1.42	s
1,135.44	2.08	2.24	285	75	75	4,199E-04	28.19	Ac-228	1.38	s
1,161.67	0.59	0.99	269	1	1	8,238E-06	1,482	Tl-208	1.36	s
1,164.31	2.15	2.21	251	32	32	1,78E-04	64.41	Ac-228	1.36	s
1,176.45	0.49	0.93	270	3	3	1,486E-05	822.2	Ac-228	1.35	s
1,215.41	1.53	1.78	296	5	5	2,661E-05	479.6	Ac-228	1.31	
1,246.22	0.16	0.62	251	38	38	2,14E-04	53.32	Ac-228	1.29	s
1,249.93	1.11	1.24	215	24	24	1,372E-04	77.64	Ac-228	1.28	s
1,286.78	0.61	0.69	190	4	4	2,524E-05	404.4	Ac-228	1.26	s
1,306.91	0.50	0.95	176	17	17	9,459E-05	102.2	Ac-228	1.24	s
1,348.04	0.12	0.22	145	23	23	1,275E-04	67.95	Ac-228	1.21	s
1,374.33	0.24	0.55	101	8	8	4,266E-05	172.9	Ac-228	1.19	s
1,401.17	0.40	0.68	108	12	12	6,855E-05	110.2	Ac-228	1.17	s
1,430.32	4.31	4.51	115	1	1	6,886E-06	1,158	Ac-228	1.15	s
**1,459.13**	**1.79**	**3.22**	**11,216**	**4,029**	**4,029**	**2,268E-02**	**3.37**	**Ac-228**	**1.13**	**D M s**
**1,460.82**	**1.79**	**3.22**	**13,228**	**7,841**	**6,014**	**3,386E-02**	**2.20**	**K-40**	**1.13**	**P D M s**
1,547.89	0.90	1.41	87	27	27	1,544E-04	42.04	Ac-228	1.08	s
1,572.87	0.18	0.50	91	28	28	1,557E-04	42.78	Ac-228	1.06	s
1,588.46	0.59	1.19	140	54	20	1,1E-04	69.15	Ac-228	1.05	s
1,625.88	1.08	1.40	74	16	16	8,797E-05	70.04	Ac-228	1.03	s
1,630.35	2.81	2.89	78	22	22	1,233E-04	50.42	Ac-228	1.03	s
1,638.10	2.44	2.52	79	5	5	2,534E-05	259.1	Ac-228	1.02	s
1,676.80	0.62	0.81	64	14	14	7,848E-05	72.81	Ac-228	1.00	s
1,703.24	0.86	1.09	74	10	10	5,772E-05	108.0	Ac-228	0.99	s
1,722.82	0.00	-1.00	69	4	4	2,071E-05	296.3	Ac-228	0.98	s
1,739.34	0.09	0.18	55	1	1	6,1E-06	908.9	Ac-228	0.97	s
1,757.13	1.50	1.66	66	8	8	4,376E-05	141.9	Ac-228	0.96	s
1,869.99	0.91	1.11	55	5	5	3,089E-05	175.9	Ac-228	0.90	s
1,886.09	1.13	1.48	55	8	8	4,452E-05	119.3	Ac-228	0.90	s

P = Peak-Background-Substraction; D = Peak area deconvoluted; E = Energy Duplication; M = Peak is part of a multiplet; s = Peak fails shape tests

Suggestions for n. ident. Peaks are based on the library "Suspected.lib" located in the config directory of LVis

Summary of identified library nuclides with peak analysis results

K-40 **Activity:** **3,7662E+02 Bq/kg** **CL: 1,1557E+01 Bq/kg** **MDA: 2,3303E+01 Bq/kg**

N°	Energy [keV]	Peakenergy [keV]	ΔE [keV]	Emission [%]		Eff. [%]	GEO Corr.	Net Area [counts]	1-σ Count.	Peakactivity [Bq/kg]		MDA [Bq/kg]	FWHM [keV]	Info
				uncorr.	corr.					time of count	Decay corr.			
1	1,460.82	1,460.82	0.00	10.550	10.550	1.131	0.6703	6,014	2.20	3,7662E+02	3,7662E+02	2,3303E+01	1,79	K G (!

Library Peak Codes:
G - Gamma Ray
X - X-Ray
P - Positron Decay
S - Single-Escape
D - Double-Escape
K - Key Line
A - Not in Average

Analysis Codes:
<> - Peak is too wide, but only one peak in library.
! - Peak is part of a multiplet and this area went negative during deconvolution.
>< - Peak is too narrow.
@ - Peak is too wide at FW25M, but ok at FWHM.
% - Peak fails sensitivity test.
$ - Peak identified, but first peak of this nuclide failed one or more qualification tests.
+ - Peak activity higher than counting uncertainty range.
- - Peak activity lower than counting uncertainty range.
= - Peak outside analysis energy range.
& - Calculated peak centroid is not close enough to the library energy centroid for positive identification.
} - Peak is too close to another for the activity to be found directly.
(= This peak was used in the nuclide activity average

Acquired, Analyzed & Saved by:

LVis Admin (D) @ GAMMA-SPEC

Reviewed & Approved by:

-
