# Supplementary material for: Yellow, Red, and Black Lepidium meyenii Preparations Differentially Modulate Doxorubicin Response and Lipid Metabolism-Related Changes in Pancreatic Cancer Cells
Source: Molecules. 2026 Nov 6;31(21):4011. doi: 10.3390/molecules31214011 (PMC13765015; doi:10.3390/molecules31214011)
Supplement: Supplementary file 1 [file molecules-31-04011-s001.zip › MACA_RED Gamma SDectrosc0DV Analvsis Report.pdf]

LVis - Gamma Spectroscopy Analysis Report

**Name:**	**MACA_RED_cylinder.lvm**
	C:\LVis\Measurements\GAMMA 1\cylinder_sample\MACA_RED_cylinder.lvm
**Detector:**	**GAMMA 1**
**Counting Routine:**	**cylinder_sample**
**Batch ID:**	**MACA_RED**

**Generator ID:**			
**Category:**		**User:**	LVis Admin (D)
**Start:**	19.09.2025 11:43:24	**Analyzed:**	16.03.2026 14:13:50
**Reference date/time:**	-	**Age of Sample:**	-
**Live Time:**	251891 s	**Total Countrate:**	3.48 counts/s
**Real Time:**	252080 s	**Dead Time:**	0.07 %
**Sample mass:**	50.60 g	**spec. Activity in:**	Bq/kg
**Sample volume:**	2.00 cm	**Reference:**	1.00
**Activity Scaling Factor:**	1,9763E+01	**Add. Uncertainties:**	0.00 %

Analysis Report according to ISO 11929

Nuclide	Detection Limit [Bq/kg]	Decision Threshold [Bq/kg]	Confidence Limits [Bq/kg] lower	upper	Best Estimate corrected [Bq/kg]	1-σ Total Uncert. [Bq/kg]	%
**K-40**	1.3318E+01	6.5938E+00	4.8942E+02	5.3198E+02	5.107E+02	1.0843E+01	2.12
**Th-234**	1.0497E+01	5.2011E+00					
**Pa-234m**	7.3897E+01	3.6376E+01					
**Th-230**	4.568E+01	2.2445E+01					
**Ra-226**	1,1274E+01	5,5986E+00					
**Pb-214**	1.4942E+00	7.4092E-01					
**Bi-214**	6,6147E+00	3,2545E+00					
**Pb-210**	2,172E+01	1,0787E+01					
**U-235D**	6.6529E+00	3.3029E+00					
**Th-227**	1,8909E+00	9,332E-01					
**Ra-223**	3.2601E+00	1.6175E+00					
**Rn-219**	1.085E+00	5.2742E-01					
**Pb-211**	1.0497E+01	5.1817E+00					
**Bi-211**	4.0788E+00	2.0224E+00					
**Ac-228**	2,1498E+00	1,0572E+00					
**Ra-224**	9,447E+00	4,6844E+00					
**Pb-212**	1,0894E+00	5,4095E-01					
**Bi-212**	6,6728E+00	3,2781E+00					
**Tl-208**	8.7188E-01	4.3157E-01					
**Cs-137**	6,4339E-01	3,1753E-01					
**Annihil**	9.8153E-01	4.8875E-01					

H = Halflife limit exceeded

Sum [Bq/kg]: 5,107E+02 +/- 1,0843E+01

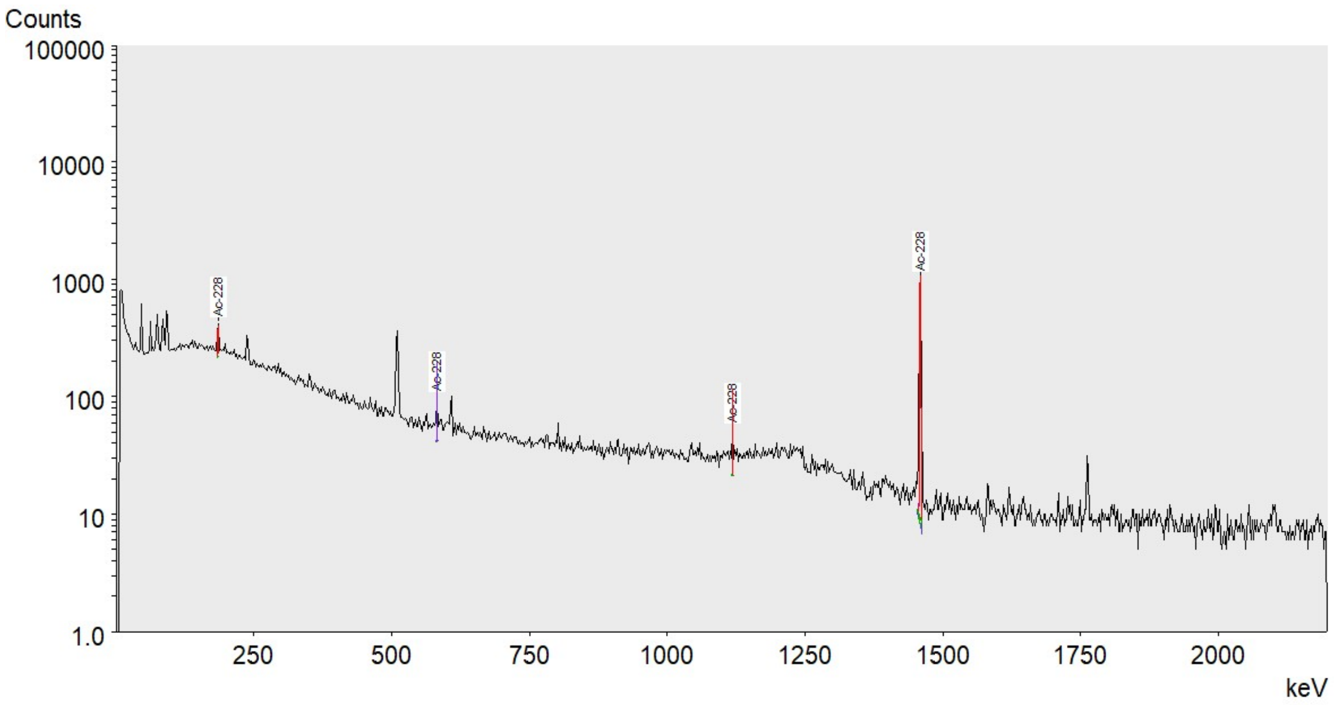

All analysis peaks

Energy [keV]	FWHM [keV]	FW10M [keV]	Gross Area [counts]	Net Area [counts]	Net - PBC [counts]	Intensity [cps]	1-σ Uncert. [%]	Nuclide	Efficiency [%]	Info
56.02	0.62	0.66	1,488	44	44	1,755E-04	113.1	Ac-228	4.15	
84.72	1.03	1.98	8,803	1,675	148	5,893E-04	111.7	Tl-208	6.33	P s
89.78	0.30	0.50	2,946	132	132	5,221E-04	61.80	Ac-228	6.46	s
99.31	0.37	0.52	1,580	36	36	1,417E-04	144.5	Ac-228	6.59	s
109.33	0.54	0.87	1,629	30	30	1,205E-04	172.7	Ac-228	6.58	s
114.67	0.39	0.49	1,722	88	88	3,513E-04	60.50	Ac-228	6.54	s
135.30	0.75	0.90	1,715	39	39	1,531E-04	139.3	Ac-228	6.22	
136.97	0.94	0.98	1,692	36	36	1,436E-04	147.6	Ac-228	6.17	
141.23	0.51	0.70	2,154	98	98	3,871E-04	64.91	Ac-228	6.11	s
153.83	0.50	0.49	1,709	35	35	1,392E-04	153.1	Ac-228	5.85	s
168.96	0.97	1.59	1,666	50	50	1,97E-04	106.6	Ac-228	5.56	
**185.43**	**1.02**	**1.55**	**6,018**	**1,409**	**1,409**	**5,592E-03**	**8.90**	**Ac-228**	**5.25**	
204.69	0.93	1.33	1,901	8	8	3,131E-05	730.6	Ac-228	4.90	
208.93	0.77	1.08	1,937	75	75	2,981E-04	76.87	Ac-228	4.81	
212.43	0.20	0.72	1,893	29	29	1,144E-04	199.0	Tl-208	4.77	s
215.42	0.13	0.23	1,818	6	6	2,247E-05	995.8	Ac-228	4.72	s
258.42	1.59	1.75	1,527	11	11	4,348E-05	471.3	Ac-228	4.12	s
269.62	3.03	3.10	1,425	6	6	2,301E-05	860.7	Ac-228	3.98	s
277.88	0.99	1.05	1,390	30	30	1,177E-04	165.6	Tl-208	3.90	
281.29	0.94	1.06	1,345	55	55	2,174E-04	87.81	Ac-228	3.85	
328.12	1.03	1.31	1,168	108	108	4,271E-04	41.19	Ac-228	3.43	
338.09	1.94	2.15	1,106	24	24	9,719E-05	178.9	Ac-228	3.35	s
341.28	0.72	0.89	1,051	46	46	1,815E-04	92.92	Ac-228	3.33	s
397.86	0.18	0.36	821	60	60	2,372E-04	62.44	Ac-228	2.96	s
399.46	0.52	0.68	767	27	27	1,059E-04	136.3	Ac-228	2.95	s
408.73	0.87	1.27	781	69	69	2,725E-04	51.29	Ac-228	2.90	
415.73	2.39	2.47	716	30	30	1,18E-04	114.4	Ac-228	2.87	s
452.75	0.85	0.96	661	53	53	2,085E-04	61.74	Ac-228	2.69	s
462.33	0.36	1.75	840	125	125	4,978E-04	29.86	Ac-228	2.65	s
471.72	0.00	-1.00	764	74	74	2,921E-04	49.14	Ac-228	2.61	s
478.22	0.50	1.01	719	51	51	2,043E-04	68.49	Ac-228	2.58	s
488.43	0.27	0.44	663	17	17	6,913E-05	196.5	Ac-228	2.54	s
515.80	0.47	0.61	1,388	129	129	5,124E-04	46.72	Ac-228	2.45	s
519.15	1.09	1.23	596	7	7	2,634E-05	490.5	Ac-228	2.43	
541.88	0.46	1.55	534	39	39	1,531E-04	78.75	Ac-228	2.36	s
548.34	1.84	1.92	574	34	34	1,359E-04	92.29	Ac-228	2.34	s
564.20	0.18	0.69	538	12	12	4,839E-05	253.0	Ac-228	2.30	s
**583.07**	**0.74**	**1.92**	**2,644**	**922**	**510**	**2,025E-03**	**16.87**	**Ac-228**	**2.24**	**s**

All analysis peaks

Energy [keV]	FWHM [keV]	FW10M [keV]	Gross Area [counts]	Net Area [counts]	Net - PBC [counts]	Intensity [cps]	1-σ Uncert. [%]	Nuclide	Efficiency [%]	Info	
583.07	0.74	1.92	2,644	510	98	3,905E-04	97.48	Tl-208	2.24	P	s
588.00	2.77	2.77	1,324	106	106	4,214E-04	59.74	Tl-208	2.23		s
610.44	0.07	0.13	293	3	3	1,191E-05	697.6	Ac-228	2.17		s
616.56	0.18	0.32	483	17	17	6,689E-05	172.9	Ac-228	2.15		s
619.75	0.54	0.89	472	31	31	1,22E-04	90.65	Ac-228	2.14		s
624.06	0.14	0.30	506	68	68	2,719E-04	41.50	Ac-228	2.14		s
627.21	0.51	0.81	442	15	15	5,902E-05	182.5	Ac-228	2.13		s
630.34	0.22	0.62	466	31	31	1,232E-04	89.14	Ac-228	2.12		s
651.07	1.01	1.15	428	5	5	2,012E-05	528.9	Tl-208	2.07		
651.14	1.37	1.48	461	74	74	2,929E-04	36.54	Ac-228	2.07		
666.12	0.16	0.47	408	6	6	2,324E-05	446.9	Ac-228	2.04		s
673.31	1.53	1.69	383	4	4	1,542E-05	652.8	Ac-228	2.02		
704.83	0.95	1.10	411	30	30	1,179E-04	87.28	Tl-208	1.96		s
726.93	0.78	0.96	397	29	29	1,141E-04	88.65	Ac-228	1.92		s
737.88	0.77	0.84	434	23	23	9,034E-05	121.8	Ac-228	1.90		s
749.28	1.26	1.38	434	53	53	2,094E-04	51.70	Tl-208	1.88		
756.17	0.65	0.75	414	21	21	8,473E-05	126.8	Ac-228	1.86		s
763.16	0.10	0.19	418	9	9	3,43E-05	317.1	Tl-208	1.85		s
775.35	0.80	2.72	434	43	43	1,706E-04	63.78	Ac-228	1.83		s
776.01	0.61	1.60	432	25	25	1,006E-04	109.0	Ac-228	1.83		s
782.39	0.13	0.23	393	19	19	7,465E-05	140.3	Ac-228	1.82		s
791.75	0.44	0.60	386	21	21	8,374E-05	123.8	Ac-228	1.80		s
794.82	1.25	1.59	443	68	68	2,702E-04	40.20	Ac-228	1.80		
808.06	0.15	0.69	1,148	91	91	3,593E-04	68.04	Tl-208	1.78		s
830.42	0.00	-1.00	348	4	4	1,54E-05	645.6	Ac-228	1.74		s
860.35	3.65	4.55	1,338	313	194	7,708E-04	31.19	Tl-208	1.70		s
870.53	0.34	0.77	358	27	27	1,09E-04	89.04	Ac-228	1.68		s
874.79	0.59	0.82	326	12	12	4,957E-05	188.2	Ac-228	1.68		s
876.80	0.23	0.39	386	66	66	2,634E-04	37.44	Ac-228	1.67		s
884.14	0.27	0.75	341	11	11	4,21E-05	226.9	Tl-208	1.67		s
911.04	1.38	1.75	1,305	432	203	8,074E-04	26.35	Ac-228	1.63		
919.46	0.25	0.41	328	15	15	5,959E-05	156.9	Ac-228	1.62		s
922.07	0.23	0.31	347	30	30	1,185E-04	80.42	Ac-228	1.62		s
927.55	0.18	0.83	353	30	30	1,197E-04	80.31	Tl-208	1.61		s
968.57	1.42	1.56	1,356	288	158	6,275E-04	40.09	Ac-228	1.56		
980.41	5.15	5.17	336	14	14	5,374E-05	176.3	Ac-228	1.55		s
989.05	0.75	0.85	350	53	53	2,095E-04	45.05	Ac-228	1.54		s
1,016.86	0.00	-1.00	308	14	14	5,716E-05	158.3	Ac-228	1.50		s
1,019.24	0.71	0.92	304	21	21	8,202E-05	109.1	Ac-228	1.50		s
1,039.12	0.00	-1.00	310	10	10	4,038E-05	225.6	Ac-228	1.48		s
1,043.01	0.14	0.34	327	13	13	5,286E-05	176.8	Ac-228	1.48		s
1,052.08	0.12	0.22	312	2	2	7,067E-06	1,301	Ac-228	1.47		s
1,065.63	0.25	0.43	345	12	12	4,798E-05	206.3	Ac-228	1.45		s
1,074.19	0.49	0.57	350	3	3	1,144E-05	877.7	Ac-228	1.44		s
1,103.67	0.14	0.25	365	24	24	9,354E-05	108.2	Ac-228	1.41		s
**1,119.63**	**0.29**	**2.55**	**924**	**220**	**220**	**8,734E-04**	**21.39**	**Ac-228**	**1.40**		**s**
1,134.80	0.79	0.92	347	7	7	2,891E-05	336.9	Ac-228	1.38		s
1,143.26	1.05	1.13	378	30	30	1,204E-04	83.35	Ac-228	1.38		s
1,175.52	0.00	-1.00	352	4	4	1,747E-05	562.3	Ac-228	1.35		s
1,185.06	2.27	2.68	395	31	31	1,225E-04	83.73	Tl-208	1.34		s
1,214.49	0.40	0.56	379	27	27	1,091E-04	92.29	Ac-228	1.31		s
1,247.63	0.13	0.24	338	17	17	6,601E-05	144.6	Ac-228	1.29		s
1,287.53	0.74	0.90	277	38	38	1,507E-04	56.29	Ac-228	1.25		s
1,310.41	0.26	0.42	233	14	14	5,717E-05	138.5	Ac-228	1.24		s
1,400.29	1.44	1.56	157	1	1	4,834E-06	1,360	Ac-228	1.17		
1,416.10	0.21	0.52	150	19	19	7,629E-05	82.10	Ac-228	1.16		s
1,452.23	0.96	1.11	264	31	31	1,213E-04	75.53	Ac-228	1.14		s
**1,460.12**	**1.69**	**3.13**	**17,383**	**14,126**	**11,534**	**4,579E-02**	**1.26**	**K-40**	**1.13**	**P**	
**1,460.12**	**1.69**	**3.12**	**14,685**	**14,052**	**14,052**	**5,579E-02**	**0.92**	**Ac-228**	**1.13**		
1,548.95	1.81	2.15	115	8	8	3,053E-05	183.0	Ac-228	1.08		
1,554.47	0.27	0.56	120	5	5	2,15E-05	266.4	Ac-228	1.07		s
1,558.64	0.60	0.74	108	3	3	1,349E-05	405.2	Ac-228	1.07		s
1,580.02	0.59	0.67	118	7	7	2,663E-05	212.9	Ac-228	1.06		s
1,588.17	0.24	0.57	317	68	18	7,254E-05	142.1	Ac-228	1.05		s
1,626.14	1.52	1.76	116	12	12	4,885E-05	113.7	Ac-228	1.03		
1,629.91	1.17	1.25	114	17	17	6,738E-05	80.83	Ac-228	1.03		s

All analysis peaks

Energy [keV]	FWHM [keV]	FW10M [keV]	Gross Area [counts]	Net Area [counts]	Net - PBC [counts]	Intensity [cps]	1-σ Uncert. [%]	Nuclide	Efficiency [%]	Info
1,666.49	1.35	2.12	114	23	23	9,197E-05	58.46	Ac-228	1.01	s
1,684.55	1.68	1.73	94	6	6	2,309E-05	218.8	Ac-228	1.00	
1,699.84	0.96	1.10	92	9	9	3,555E-05	139.9	Ac-228	0.99	s
1,823.22	0.33	0.49	80	2	2	9,323E-06	505.1	Ac-228	0.93	s
1,871.22	2.94	3.02	99	28	28	1,098E-04	44.84	Ac-228	0.90	s
1,930.27	0.36	0.48	68	4	4	1,565E-05	269.4	Ac-228	0.88	s
1,952.26	0.75	0.87	78	4	4	1,609E-05	281.3	Ac-228	0.87	s
1,965.47	0.18	0.52	80	16	16	6,401E-05	69.35	Ac-228	0.86	s

P = Peak-Background-Substraction; D = Peak area deconvoluted; E = Energy Duplication; M = Peak is part of a multiplet; s = Peak fails shape tests

Suggestions for n. ident. Peaks are based on the library "Suspected.lib" located in the config directory of LVis

Summary of identified library nuclides with peak analysis results

K-40 **Activity: 5,107E+02 Bq/kg** **CL: 6,5938E+00 Bq/kg** **MDA: 1,3318E+01 Bq/kg**

N°	Energy [keV]	Peakenergy [keV]	ΔE [keV]	Emission [%] uncorr.	Emission [%] corr.	Eff. [%]	GEO Corr.	Net Area [counts]	1-σ Count.	Peakactivity [Bq/kg] time of count	Peakactivity [Bq/kg] Decay corr.	MDA [Bq/kg]	FWHM [keV]	Info
1	1,460.82	1,460.12	0.70	10.550	10.550	1.131	0.6734	11,534	1.26	5,107E+02	5,107E+02	1,3318E+01	1,69	K G (

Library Peak Codes:
G - Gamma Ray
X - X-Ray
P - Positron Decay
S - Single-Escape
D - Double-Escape
K - Key Line
A - Not in Average

Analysis Codes:
<> - Peak is too wide, but only one peak in library.
! - Peak is part of a multiplet and this area went negative during deconvolution.
>< - Peak is too narrow.
@ - Peak is too wide at FW25M, but ok at FWHM.
% - Peak fails sensitivity test.
$ - Peak identified, but first peak of this nuclide failed one or more qualification tests.
+ - Peak activity higher than counting uncertainty range.
- - Peak activity lower than counting uncertainty range.
= - Peak outside analysis energy range.
& - Calculated peak centroid is not close enough to the library energy centroid for positive identification.
} - Peak is too close to another for the activity to be found directly.
(= This peak was used in the nuclide activity average

Acquired, Analyzed & Saved by:

LVis Admin (D) @ GAMMA-SPEC

Reviewed & Approved by:

-
