# Supplementary material for: Yellow, Red, and Black Lepidium meyenii Preparations Differentially Modulate Doxorubicin Response and Lipid Metabolism-Related Changes in Pancreatic Cancer Cells
Source: Molecules. 2026 Nov 6;31(21):4011. doi: 10.3390/molecules31214011 (PMC13765015; doi:10.3390/molecules31214011)
Supplement: Supplementary file 1 [file molecules-31-04011-s001.zip › MACA_YELLOW Gamma SDectrosc0DV Analvsis Report.pdf]

LVis - Gamma Spectroscopy Analysis Report

**Name:**	**MACA_YELLOW.lvm**<br>*C:\LVis\Measurements\GAMMA 1\cylinder_sample\MACA_YELLOW.lvm*
**Detector:**	**GAMMA 1**
**Counting Routine:**	**cylinder_sample**
**Batch ID:**	**MACA_YELLOW**
**Generator ID:**	**MACA_YELLOW**
**Category:**	**MACA_YELLOW**

**Start:**	9/26/2025 13:59:54	**Analyzed:**	9/29/2025 15:17:26
**Reference date/time:**	-	**Age of Sample:**	-
**Live Time:**	263547 s	**Total Countrate:**	3.45 counts/s
**Real Time:**	263743 s	**Dead Time:**	0.07 %
**Sample mass:**	51.50 g	**spec. Activity in:**	Bq/kg
**Sample volume:**	2.00 cm	**Reference:**	1.00
**Activity Scaling Factor:**	1.942E+001	**Add. Uncertainties:**	0.00 %

Analysis Report according to ISO 11929

Nuclide	Detection Limit [Bq/kg]	Decision Threshold [Bq/kg]	Confidence Limits [Bq/kg]		Best Estimate corrected [Bq/kg]	1-σ Total Uncert.	
			lower	upper		[Bq/kg]	%
**K-40**	1.349E+001	6.665E+000	4.728E+002	5.411E+002	5.070E+002	1.740E+001	3.43
**Th-234**	1.129E+001	5.589E+000					
**Pa-234m**	9.885E+001	4.875E+001					
**Th-230**	2.875E+001	1.399E+001					
**Ra-226**	1.384E+001	6.866E+000					
**Pb-214**	1.173E+000	5.789E-001					
**Bi-214**	6.626E+000	3.255E+000					
**Pb-210**	1.720E+001	8.527E+000					
**U-235D**	5.233E+000	2.587E+000					
**Th-227**	2.067E+000	1.019E+000					
**Ra-223**	3.557E+000	1.762E+000					
**Rn-219**	1.642E+000	8.037E-001					
**Pb-211**	1.157E+001	5.708E+000					
**Bi-211**	3.201E+000	1.580E+000					
**Ac-228**	2.796E+000	1.377E+000					
**Ra-224**	8.513E+000	4.208E+000					
**Pb-212**	1.144E+000	5.672E-001					
**Bi-212**	8.276E+000	4.070E+000					
**Tl-208**	7.545E-001	3.721E-001					
**Cs-137**	3.615E-001	1.763E-001					
**Annihil**	1.063E+000	5.283E-001					

H = Halflife limit exceeded

Sum [Bq/kg]: 5.070E+002 +/- 1.740E+001

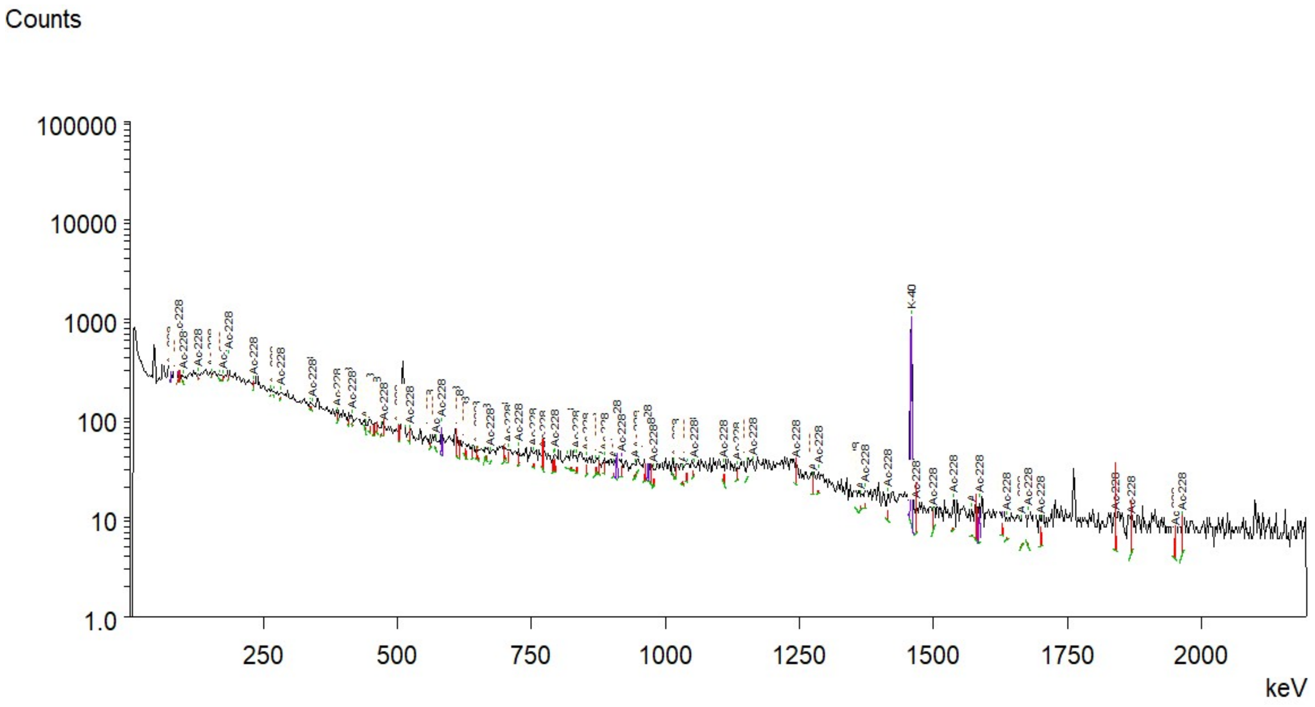

All analysis peaks

Energy [keV]	FWHM [keV]	FW10M [keV]	Gross Area [counts]	Net Area [counts]	Net - PBC [counts]	Intensity [cps]	1-σ Uncert. [%]	Nuclide	Efficiency [%]	Info
77.09	0.88	1.09	4,238	376	376	1.43E-003	27.67	Ac-228	6.01	
89.88	1.53	1.67	4,613	307	307	1.16E-003	37.46	Ac-228	6.46	s
**92.64**	**1.05**	**1.95**	**9,086**	**2,500**	**2,500**	**9.49E-003**	**6.72**	**Ac-228**	**6.53**	**s**
100.13	1.55	1.55	1,663	96	96	3.66E-004	54.53	Ac-228	6.59	s
129.41	0.68	0.84	1,744	49	49	1.87E-004	110.1	Ac-228	6.33	
153.60	0.24	0.42	1,794	31	31	1.19E-004	174.9	Ac-228	5.85	s
168.27	1.46	1.66	1,757	65	65	2.47E-004	83.29	Ac-228	5.56	s
173.99	0.38	0.63	1,734	75	75	2.85E-004	71.52	Ac-228	5.45	s
184.34	0.17	0.49	2,186	36	36	1.36E-004	178.8	Ac-228	5.25	s
231.18	0.23	0.36	1,820	77	77	2.91E-004	72.95	Ac-228	4.47	s
263.37	0.35	0.53	1,496	6	6	2.41E-005	804.0	Ac-228	4.05	s
269.67	2.95	3.02	1,483	6	6	2.31E-005	836.3	Ac-228	3.98	s
282.44	1.09	1.14	1,425	49	49	1.85E-004	101.9	Ac-228	3.85	
338.17	1.27	1.47	1,184	107	107	4.04E-004	41.88	Ac-228	3.35	s
341.14	1.39	1.47	1,077	19	19	7.14E-005	229.7	Ac-228	3.33	s
389.54	0.18	0.60	865	25	25	9.61E-005	152.6	Ac-228	3.01	s
409.26	0.75	0.90	830	81	81	3.06E-004	44.94	Ac-228	2.90	s
415.00	1.07	1.21	801	46	46	1.75E-004	77.86	Ac-228	2.87	
440.50	0.40	0.58	687	19	19	7.14E-005	177.6	Ac-228	2.75	s
447.46	0.55	1.11	691	48	48	1.81E-004	69.52	Ac-228	2.71	s
456.91	0.66	1.04	835	109	109	4.14E-004	34.32	Ac-228	2.67	s
462.20	0.19	0.96	807	30	30	1.16E-004	123.6	Ac-228	2.65	s
470.31	0.75	0.92	742	24	24	9.04E-005	151.7	Ac-228	2.62	s
474.63	0.16	0.41	763	38	38	1.46E-004	95.01	Ac-228	2.60	s
503.56	0.45	0.59	1,775	93	93	3.53E-004	77.98	Ac-228	2.49	s
515.46	0.00	-1.00	608	1	1	5.42E-006	2,305	Ac-228	2.45	s
523.36	0.13	0.23	644	22	22	8.25E-005	154.8	Ac-228	2.42	s
562.17	0.29	0.70	558	14	14	5.49E-005	217.1	Ac-228	2.30	s
571.49	1.91	2.05	557	30	30	1.15E-004	103.2	Ac-228	2.27	s
583.23	1.44	2.17	2,157	444	13	0.00E+000	609.1	Ac-228	2.24	P
611.10	0.14	0.26	1,121	103	103	3.91E-004	52.56	Ac-228	2.17	s
615.87	0.21	1.00	509	81	81	3.08E-004	35.83	Ac-228	2.15	s
625.90	0.29	1.12	471	16	16	6.18E-005	171.8	Ac-228	2.13	s
629.31	0.15	0.91	429	10	10	3.79E-005	267.8	Ac-228	2.12	s
641.18	0.19	0.61	442	13	13	5.04E-005	204.6	Ac-228	2.10	s
648.95	0.75	1.43	510	90	90	3.43E-004	31.29	Ac-228	2.08	s
651.57	0.88	0.99	426	18	18	6.72E-005	150.1	Ac-228	2.07	s
663.09	0.50	0.60	418	2	2	6.34E-006	1,588	Ac-228	2.04	s
664.90	0.96	1.00	431	17	17	6.52E-005	155.7	Ac-228	2.04	s

All analysis peaks

Energy [keV]	FWHM [keV]	FW10M [keV]	Gross Area [counts]	Net Area [counts]	Net - PBC [counts]	Intensity [cps]	1-σ Uncert. [%]	Nuclide	Efficiency [%]	Info
675.55	0.66	0.96	397	3	3	1.27E-005	770.0	Ac-228	2.02	s
699.51	0.26	0.36	430	37	37	1.39E-004	72.37	Ac-228	1.97	s
701.87	1.00	1.12	448	30	30	1.15E-004	89.25	Ac-228	1.96	s
707.41	0.59	0.86	430	43	43	1.63E-004	61.29	Ac-228	1.95	s
727.07	1.15	1.61	451	85	85	3.23E-004	31.20	Ac-228	1.92	
755.72	1.11	1.82	438	27	27	1.03E-004	101.9	Ac-228	1.86	
772.01	0.24	1.50	451	58	58	2.21E-004	47.53	Ac-228	1.83	s
791.48	0.56	0.72	409	41	41	1.54E-004	65.65	Ac-228	1.80	s
794.60	1.04	1.29	449	76	76	2.90E-004	35.84	Ac-228	1.80	s
816.43	2.52	2.60	404	3	3	1.16E-005	882.4	Ac-228	1.76	s
826.31	1.71	1.76	414	30	30	1.14E-004	89.75	Ac-228	1.75	s
830.40	0.54	0.89	383	2	2	8.61E-006	1,161	Ac-228	1.74	s
835.62	1.80	2.07	413	50	50	1.90E-004	53.04	Ac-228	1.73	s
853.59	0.90	1.00	395	47	47	1.80E-004	54.94	Ac-228	1.71	s
872.79	1.03	1.17	385	42	42	1.58E-004	60.47	Ac-228	1.68	s
872.87	1.04	1.24	395	32	32	1.23E-004	78.97	Ac-228	1.68	s
876.93	0.24	0.53	365	19	19	7.21E-005	130.5	Ac-228	1.67	s
887.03	0.66	1.21	394	40	40	1.50E-004	64.37	Ac-228	1.66	s
903.77	1.14	1.23	352	21	21	7.91E-005	116.7	Ac-228	1.64	s
910.56	2.32	2.92	1,747	372	133	0.00E+000	56.72	Ac-228	1.63	P s
918.35	0.52	0.72	362	28	28	1.06E-004	88.00	Ac-228	1.62	s
945.09	1.98	2.07	355	33	33	1.24E-004	74.28	Ac-228	1.59	s
949.03	1.11	1.30	366	8	8	3.09E-005	306.5	Ac-228	1.58	s
962.82	0.19	0.29	1,153	118	118	4.48E-004	51.83	Ac-228	1.56	s
968.27	1.45	2.28	1,316	249	113	0.00E+000	55.02	Ac-228	1.56	P
973.91	0.38	0.60	1,203	104	104	3.93E-004	63.39	Ac-228	1.55	s
978.98	1.44	1.64	307	40	40	1.51E-004	56.27	Ac-228	1.55	
1,015.04	4.20	4.44	353	20	20	7.45E-005	124.1	Ac-228	1.50	s
1,019.99	0.12	0.22	331	7	7	2.66E-005	339.7	Ac-228	1.50	s
1,033.10	2.12	2.17	302	29	29	1.12E-004	75.90	Ac-228	1.49	s
1,041.55	0.72	1.11	324	30	30	1.12E-004	78.29	Ac-228	1.48	s
1,052.62	0.14	0.29	336	4	4	1.64E-005	556.0	Ac-228	1.47	s
1,055.10	2.68	2.73	354	24	24	8.99E-005	102.8	Ac-228	1.46	s
1,110.79	1.41	1.50	381	58	58	2.19E-004	44.26	Ac-228	1.41	
1,135.07	0.59	0.78	385	32	32	1.22E-004	79.50	Ac-228	1.38	s
1,155.39	0.17	0.34	365	1	1	5.54E-006	1,729	Ac-228	1.37	s
1,164.79	0.00	-1.00	357	2	2	6.67E-006	1,422	Ac-228	1.36	s
1,244.84	0.22	0.35	352	31	31	1.17E-004	78.86	Ac-228	1.29	s
1,278.27	0.35	0.60	275	21	21	8.04E-005	101.9	Ac-228	1.26	s
1,285.50	1.30	1.38	272	12	12	4.42E-005	185.4	Ac-228	1.26	s
1,355.81	0.68	0.98	180	3	3	1.03E-005	651.0	Ac-228	1.20	s
1,363.95	0.77	0.96	182	7	7	2.53E-005	266.0	Ac-228	1.20	s
1,376.26	0.40	0.54	189	4	4	1.61E-005	426.2	Ac-228	1.19	s
1,415.49	0.41	0.60	146	8	8	2.89E-005	207.5	Ac-228	1.16	s
**1,460.14**	**1.77**	**3.24**	**17,723**	**14,322**	**11,610**	**0.00E+000**	**1.27**	**K-40**	**1.13**	**P**
1,469.58	0.25	0.45	148	32	32	1.20E-004	48.90	Ac-228	1.13	s
1,503.32	0.16	0.58	133	5	5	1.98E-005	291.2	Ac-228	1.10	s
1,537.84	3.44	3.50	136	11	11	4.24E-005	136.2	Ac-228	1.08	s
1,574.12	1.91	2.03	112	3	3	1.01E-005	528.0	Ac-228	1.06	
1,581.90	0.71	1.26	366	63	63	2.41E-004	51.82	Ac-228	1.06	s
1,587.34	2.20	2.42	376	113	61	0.00E+000	48.61	Ac-228	1.05	P
1,631.43	0.59	0.83	121	10	10	3.80E-005	143.3	Ac-228	1.03	s
1,637.94	0.74	0.88	102	2	2	6.91E-006	736.2	Ac-228	1.02	s
1,667.13	3.18	3.19	92	5	5	2.00E-005	239.2	Ac-228	1.01	s
1,678.27	0.50	0.64	89	1	1	4.08E-006	1,165	Ac-228	1.00	s
1,702.95	0.90	1.69	107	20	20	7.51E-005	66.73	Ac-228	0.99	s
1,841.35	0.06	0.10	92	14	14	5.46E-005	85.89	Ac-228	0.92	s
1,871.73	0.31	0.52	97	25	25	9.33E-005	50.39	Ac-228	0.90	s
1,951.34	0.42	0.80	80	14	14	5.46E-005	77.95	Ac-228	0.87	s
1,965.37	0.15	0.46	77	8	8	3.04E-005	140.5	Ac-228	0.86	s

P = Peak-Background-Substraction; D = Peak area deconvoluted; E = Energy Duplication; M = Peak is part of a multiplet; s = Peak fails shape tests

Suggestions for n. ident. Peaks are based on the library "Suspected.lib" located in the config directory of LVis

Summary of identified library nuclides with peak analysis results

Ra-226 Activity: < MDA CL: 6.866E+000 Bq/kg MDA: 1.384E+001 Bq/kg

N°	Energy [keV]	Peakenergy [keV]	ΔE [keV]	Emission [%] uncorr.	Emission [%] corr.	Eff. [%]	GEO Corr.	Net Area [counts]	1-σ Count.	Peakactivity [Bq/kg] time of count	Peakactivity [Bq/kg] Decay corr.	MDA [Bq/kg]	FWHM [keV]	Info
1	94.85				0.110	6.546	0.6499					1.438E+002		K X ><

Pb-214 Activity: < MDA CL: 5.789E-001 Bq/kg MDA: 1.173E+000 Bq/kg

N°	Energy [keV]	Peakenergy [keV]	ΔE [keV]	Emission [%] uncorr.	Emission [%] corr.	Eff. [%]	GEO Corr.	Net Area [counts]	1-σ Count.	Peakactivity [Bq/kg] time of count	Peakactivity [Bq/kg] Decay corr.	MDA [Bq/kg]	FWHM [keV]	Info
1	351.93				35.561	3.249	0.6735					1.173E+000		K G ><
2	295.22			18.414	18.430	3.716	0.6692					2.621E+000		G
3	242.00			7.268	7.249	4.323	0.6638					3.420E+000		G ><
4	785.96			1.064	1.063	1.812	0.6939					4.387E+001		G ><
5	53.23			1.060	0.949	3.625	0.6624					4.635E+001		G %
6	839.04			0.587	0.636	1.729	0.6953					7.412E+001		A G ><
7	258.87			0.532	0.478	4.108	0.6654					8.518E+001		A G %
8	487.09			0.433	0.386	2.551	0.6818					1.144E+002		A G ><
9	580.13			0.369	0.331	2.248	0.6863					1.444E+002		A G ><
10	274.80			0.362	0.323	3.925	0.6671					3.967E+001		A G ><
11	480.43			0.337	0.337	2.576	0.6814					1.220E+002		A G %
12	462.00			0.213	0.213	2.651	0.6804					2.457E+002		A G ><
13	533.66			0.182	0.210	2.387	0.6843					1.186E+002		A G ><
14	314.32			0.077	0.077	3.542	0.6706					5.899E+002		A G %
15	196.20			0.069	0.069	5.033	0.6590					4.319E+002		A G ><
16	765.96			0.053	0.053	1.845	0.6931					1.022E+003		A G ><
17	543.81			0.050	0.045	2.355	0.6848					1.031E+003		A G ><
18	137.45			0.045	0.045	6.183	0.6523					3.648E+002		A G ><
19	305.26			0.032	0.027	3.622	0.6700					1.282E+003		A G ><
20	323.83			0.029	0.029	3.462	0.6715					1.378E+003		A G ><
21	141.30			0.027	0.027	6.108	0.6527					4.874E+002		A G ><
22	538.41			0.020	0.020	2.372	0.6846					2.056E+003		A G %
23	170.07			0.015	0.015	5.527	0.6560					2.071E+003		A G %
24	205.68			0.011	0.010	4.870	0.6600					2.840E+003		A G %
25	216.47			0.010	0.010	4.694	0.6611					1.200E+003		A G ><
26	74.82			6.260	6.260	5.865	0.6526					6.111E+000		A X
27	77.11			10.470	10.470	5.997	0.6523					2.729E+000		A X ><

K-40 Activity: 5.070E+002 Bq/kg CL: 6.665E+000 Bq/kg MDA: 1.349E+001 Bq/kg

N°	Energy [keV]	Peakenergy [keV]	ΔE [keV]	Emission [%] uncorr.	Emission [%] corr.	Eff. [%]	GEO Corr.	Net Area [counts]	1-σ Count.	Peakactivity [Bq/kg] time of count	Peakactivity [Bq/kg] Decay corr.	MDA [Bq/kg]	FWHM [keV]	Info
1	**1,460.82**	**1,460.14**	**0.68**	**10.550**	**10.550**	**1.131**	**0.7072**	**11,610**	**1.27**	**5.070E+002**	**5.070E+002**	**1.349E+001**	**1.77**	K G (

Th-234 Activity: < MDA CL: 5.589E+000 Bq/kg MDA: 1.129E+001 Bq/kg

N°	Energy [keV]	Peakenergy [keV]	ΔE [keV]	Emission [%] uncorr.	Emission [%] corr.	Eff. [%]	GEO Corr.	Net Area [counts]	1-σ Count.	Peakactivity [Bq/kg] time of count	Peakactivity [Bq/kg] Decay corr.	MDA [Bq/kg]	FWHM [keV]	Info
1	63.30				3.750	4.918	0.6571					1.129E+001		K G
2	92.38			2.180	2.180	6.510	0.6502					1.785E+001		A G
3	92.80			2.150	2.150	6.517	0.6502					1.808E+001		A G
4	112.81			0.215	0.215	6.554	0.6501					1.483E+002		A G %

Pa-234m Activity: < MDA CL: 4.875E+001 Bq/kg MDA: 9.885E+001 Bq/kg

N°	Energy [keV]	Peakenergy [keV]	ΔE [keV]	Emission [%] uncorr.	Emission [%] corr.	Eff. [%]	GEO Corr.	Net Area [counts]	1-σ Count.	Peakactivity [Bq/kg] time of count	Peakactivity [Bq/kg] Decay corr.	MDA [Bq/kg]	FWHM [keV]	Info
1	1,001.03				0.851	1.521	0.6991					9.885E+001		K G ><
2	766.36			0.323	0.322	1.845	0.6931					1.693E+002		G ><
3	98.44			0.316	0.316	6.580	0.6494					8.193E+001		A X ><
4	94.67			0.197	0.197	6.544	0.6499					8.006E+001		A X ><
5	111.23			0.115	0.115	6.567	0.6499					2.116E+002		A X ><

Th-230 Activity: < MDA CL: 1.399E+001 Bq/kg MDA: 2.875E+001 Bq/kg

N°	Energy [keV]	Peakenergy [keV]	ΔE [keV]	Emission [%] uncorr.	Emission [%] corr.	Eff. [%]	GEO Corr.	Net Area [counts]	1-σ Count.	Peakactivity [Bq/kg] time of count	Peakactivity [Bq/kg] Decay corr.	MDA [Bq/kg]	FWHM [keV]	Info
1	67.67				0.380	5.338	0.6546					2.875E+001		K G ><

Ra-226 Activity: < MDA CL: 6.866E+000 Bq/kg MDA: 1.384E+001 Bq/kg

N°	Energy [keV]	Peakenergy [keV]	ΔE [keV]	Emission [%] uncorr.	Emission [%] corr.	Eff. [%]	GEO Corr.	Net Area [counts]	1-σ Count.	Peakactivity [Bq/kg] time of count	Peakactivity [Bq/kg] Decay corr.	MDA [Bq/kg]	FWHM [keV]	Info
1	186.21				3.555	5.215	0.6578					1.384E+001		K G &
2	83.78			0.317	0.317	6.292	0.6513					1.311E+002		A X &
3	81.07			0.192	0.192	6.187	0.6517					1.539E+002		A X %

Pb-214 Activity: < MDA CL: 5.789E-001 Bq/kg MDA: 1.173E+000 Bq/kg

N°	Energy [keV]	Peakenergy [keV]	ΔE [keV]	Emission [%] uncorr.	Emission [%] corr.	Eff. [%]	GEO Corr.	Net Area [counts]	1-σ Count.	Peakactivity [Bq/kg] time of count	Peakactivity [Bq/kg] Decay corr.	MDA [Bq/kg]	FWHM [keV]	Info
1	87.35				3.590	6.402	0.6509					8.511E+000		K X
2	90.08			1.100	1.100	6.467	0.6506					2.637E+001		A X %

Bi-214 Activity: < MDA CL: 3.255E+000 Bq/kg MDA: 6.626E+000 Bq/kg

N°	Energy [keV]	Peakenergy [keV]	ΔE [keV]	Emission [%] uncorr.	Emission [%] corr.	Eff. [%]	GEO Corr.	Net Area [counts]	1-σ Count.	Peakactivity [Bq/kg] time of count	Peakactivity [Bq/kg] Decay corr.	MDA [Bq/kg]	FWHM [keV]	Info

1	1,764.49		15.356	0.956	0.7112	6.626E+000	K	G		
2	609.31	45.490	40.784	2.171	0.6873	1.287E+000	K	G		
3	1,120.29	14.910	13.226	1.398	0.7019	6.151E+000		G		><
4	1,238.11	5.831	5.203	1.294	0.7043	1.604E+001		G		><
5	2,204.21	4.913	4.930	0.763	0.6591			G	=	
6	768.36	4.892	4.281	1.841	0.6933	8.216E+000	A	G		><
7	1,377.67	3.968	4.139	1.188	0.7061	1.326E+001	A	G		><
8	934.06	3.100	2.738	1.600	0.6975	2.252E+001	A	G		><
9	1,729.59	2.844	3.494	0.974	0.7108	1.772E+001	A	G		><
10	1,407.98	2.389	2.129	1.167	0.7065	1.993E+001	A	G		><
11	1,509.23	2.128	1.936	1.100	0.7078	2.434E+001	A	G		><
12	1,847.42	2.025	2.285	0.915	0.7123	3.246E+001	A	G		><
13	1,155.19	1.635	1.463	1.366	0.7027	6.081E+001	A	G		><
14	2,447.86	1.548	1.575	0.678	0.6591		A	G	=	
15	665.45	1.530	1.236	2.039	0.6894	4.924E+001	A	G		><
16	1,280.96	1.435	1.279	1.260	0.7048	4.641E+001	A	G		><
17	1,401.50	1.330	1.185	1.171	0.7064	5.921E+001	A	G		><
18	806.17	1.262	1.073	1.779	0.6946	3.237E+001	A	G		><
19	2,118.55	1.158	1.253	0.797	0.7152	4.194E+001	A	G		><
20	1,661.28	1.048	1.013	1.011	0.7099	5.336E+001	A	G		><
21	1,385.31	0.795	0.726	1.182	0.7062	4.086E+001	A	G		><
22	1,583.22	0.707	0.633	1.055	0.7088	9.273E+001	A	G		><
23	703.11	0.479	0.382	1.961	0.6909	1.038E+002	A	G		><
24	1,207.68	0.454	0.361	1.320	0.7039	9.843E+001	A	G		><
25	1,538.50	0.401	0.357	1.082	0.7083	2.113E+002	A	G		><
26	388.88	0.394	0.323	3.013	0.6763	1.367E+002	A	G		><
27	719.86	0.393	0.311	1.928	0.6914	2.256E+002	A	G		><
28	964.08	0.363	0.327	1.564	0.6982	2.139E+002	A	G		><
29	1,838.36	0.343	0.321	0.919	0.7122	1.361E+002	A	G		><
30	1,051.96	0.324	0.275	1.466	0.7003	2.657E+002	A	G		><
31	1,599.31	0.322	0.287	1.046	0.7090	1.370E+002	A	G		><
32	786.10	0.310	0.275	1.811	0.6939	1.696E+002	A	G		><
33	2,293.40	0.306	0.316	0.731	0.6591		A	G	=	
34	1,543.32	0.302	0.440	1.079	0.7083	3.663E+001	A	G		><
35	386.77	0.000	0.250	3.026	0.6761	5.148E+001	A	G		><
36	454.77	0.288	0.227	2.682	0.6800	1.682E+002	A	G		%
37	1,594.73	0.274	0.252	1.048	0.7090	2.646E+002	A	G		&
38	1,069.96	0.271	0.230	1.448	0.7007	3.868E+002	A	G		%
39	1,133.66	0.254	0.224	1.386	0.7022	2.518E+002	A	G		><
40	1,683.99	0.217	0.194	0.998	0.7101	2.864E+002	A	G		><
41	1,873.16	0.212	0.215	0.903	0.7127	3.161E+002	A	G		><
42	405.74	0.171	0.171	2.919	0.6774	1.633E+002	A	G		><
43	821.18	0.166	0.147	1.756	0.6949	2.656E+002	A	G		><
44	1,895.92	0.146	0.131	0.892	0.7130	3.100E+002	A	G		><
45	469.76	0.133	0.112	2.619	0.6809	3.593E+002	A	G		><
46	826.30	0.129	0.109	1.748	0.6950	3.006E+002	A	G		><
47	752.84	0.126	0.103	1.868	0.6926	4.104E+002	A	G		><
48	348.92	0.123	0.123	3.271	0.6733	2.775E+002	A	G		><
49	273.80	0.120	0.120	3.936	0.6670	1.862E+002	A	G		><
50	1,103.64	0.106	0.090	1.414	0.7015	5.354E+002	A	G		><
51	1,303.76	0.105	0.084	1.242	0.7051	1.665E+002	A	G		><
52	474.41	0.092	0.075	2.600	0.6811	4.526E+002	A	G		><
53	543.00	0.088	0.078	2.358	0.6848	5.905E+002	A	G		><
54	683.22	0.084	0.068	2.001	0.6901	3.183E+002	A	G		><
55	2,109.92	0.084	0.080	0.800	0.7151	7.503E+002	A	G		><
56	1,890.30	0.078	0.122	0.895	0.7129	3.335E+002	A	G		><
57	1,316.96	0.077	0.065	1.232	0.7053	5.452E+002	A	G		><
58	710.67	0.076	0.064	1.946	0.6911	8.209E+002	A	G		><
59	1,104.79	0.073	0.073	1.413	0.7015	4.910E+002	A	G		><
60	572.76	0.071	0.056	2.269	0.6860	2.860E+002	A	G		><
61	2,052.94	0.069	0.062	0.823	0.7147	7.915E+002	A	G		><
62	697.90	0.067	0.067	1.971	0.6906	1.929E+002	A	G		><
63	904.29	0.066	0.054	1.638	0.6968	7.487E+002	A	G		><
64	333.35	0.063	0.063	3.386	0.6721	5.951E+002	A	G		><
65	280.95	0.062	0.050	3.860	0.6677	7.282E+002	A	G		><
66	1,032.37	0.061	0.049	1.487	0.6999	8.923E+002	A	G		><
67	536.77	0.061	0.049	2.377	0.6844	9.526E+002	A	G		%
68	221.00	0.059	0.059	4.624	0.6616	2.904E+002	A	G		><
69	461.00	0.058	0.058	2.656	0.6804	9.007E+002	A	G		><
70	633.14	0.055	0.046	2.112	0.6882	1.122E+003	A	G		><
71	615.73	0.055	0.055	2.155	0.6876	6.094E+002	A	G		><
72	1,172.98	0.055	0.044	1.350	0.7031	1.824E+003	A	G		><
73	649.18	0.054	0.044	2.075	0.6888	1.311E+003	A	G		><
74	661.10	0.054	0.044	2.048	0.6893	7.020E+002	A	G		><
75	1,479.15	0.051	0.046	1.119	0.7074	1.125E+003	A	G		><
76	704.90	0.051	0.051	1.957	0.6908	6.758E+002	A	G		><
77	1,898.70	0.049	0.044	0.891	0.7130	9.153E+002	A	G		%
78	1,657.00	0.047	0.043	1.013	0.7098	8.124E+002	A	G		><
79	2,089.70	0.044	0.040	0.808	0.7150	3.114E+002	A	G		><
80	2,010.78	0.043	0.095	0.841	0.7144	3.907E+002	A	G		><
81	930.20	0.043	0.043	1.605	0.6974	7.489E+002	A	G		><
82	740.73	0.043	0.036	1.890	0.6922	1.418E+003	A	G		><
83	815.00	0.039	0.033	1.765	0.6948	1.379E+003	A	G		><
84	2,192.58	0.038	0.066	0.768	0.6591		A	G	=	

85	733.80			0.038	0.029	1.902	0.6920					1.618E+003		A G ><
86	722.98			0.037	0.029	1.922	0.6916					1.093E+003		A G ><
87	1,130.29			0.036	0.036	1.389	0.7021					6.471E+002		A G ><
88	832.39			0.035	0.035	1.739	0.6952					6.724E+002		A G ><
89	547.60			0.034	0.034	2.343	0.6852					2.101E+003		A G ><
90	639.67			0.034	0.034	2.097	0.6885					1.552E+003		A G ><
91	334.78			0.033	0.033	3.375	0.6723					4.574E+002		A G ><
92	2,694.70			0.033	0.035	0.603	0.6591							A G =
93	1,935.50			0.032	0.029	0.874	0.7135					1.912E+003		A G ><
94	452.92			0.031	0.031	2.690	0.6799					1.425E+003		A G ><
95	487.95			0.028	0.028	2.547	0.6818					1.885E+003		A G ><
96	617.00			0.027	0.023	2.152	0.6876					1.474E+003		A G ><
97	2,331.30			0.026	0.023	0.717	0.6591							A G =
98	396.01			0.026	0.020	2.973	0.6769					2.707E+003		A G ><
99	304.20			0.025	0.025	3.632	0.6700					1.276E+003		A G ><
100	1,067.20			0.024	0.021	1.451	0.7007					2.841E+003		A G ><
101	1,711.00			0.023	0.023	0.984	0.7105					1.973E+003		A G ><
102	1,045.60			0.023	0.023	1.473	0.7002					2.839E+003		A G %
103	915.74			0.023	0.019	1.624	0.6971					2.785E+003		A G ><
104	2,769.90			0.023	0.023	0.582	0.6591							A G =
105	3,053.88			0.022	0.022	0.510	0.6591							A G =
106	1,341.49			0.021	0.021	1.214	0.7056					9.264E+002		A G ><
107	2,021.60			0.021	0.019	0.837	0.7145					3.184E+003		A G ><
108	485.92			0.021	0.021	2.555	0.6818					2.097E+003		A G ><
109	873.07			0.019	0.019	1.680	0.6961					3.527E+003		A G ><
110	501.96			0.018	0.016	2.495	0.6826					1.137E+003		A G ><
111	1,226.70			0.018	0.015	1.304	0.7042					4.651E+003		A G ><
112	595.23			0.017	0.017	2.207	0.6868					1.691E+003		A G ><
113	658.70			0.017	0.017	2.054	0.6892					2.106E+003		A G ><
114	943.34			0.017	0.017	1.589	0.6977					3.922E+003		A G ><
115	524.60			0.017	0.015	2.417	0.6838					8.328E+002		A G ><
116	630.79			0.017	0.017	2.118	0.6882					3.120E+003		A G ><
117	519.90			0.017	0.017	2.433	0.6836					2.942E+003		A G ><
118	2,266.51			0.016	0.020	0.740	0.6591							A G =
119	268.80			0.016	0.016	3.992	0.6665					2.492E+003		A G ><
120	699.82			0.016	0.016	1.967	0.6907					4.031E+003		A G %
121	1,021.00			0.016	0.016	1.499	0.6996					4.199E+003		A G ><
122	939.60			0.016	0.016	1.593	0.6976					3.102E+003		A G ><
123	847.16			0.016	0.016	1.717	0.6955					1.059E+003		A G ><
124	976.18			0.015	0.012	1.550	0.6985					2.494E+003		A G ><
125	788.60			0.015	0.015	1.807	0.6939					3.966E+003		A G %
126	1,665.80			0.015	0.015	1.008	0.7099					2.421E+003		A G ><
127	2,978.90			0.014	0.014	0.528	0.6591							A G =
128	2,147.90			0.013	0.029	0.785	0.6591							A G =
129	2,921.90			0.013	0.014	0.542	0.6591							A G =
130	1,284.00			0.013	0.013	1.258	0.7049					6.584E+003		A G ><
131	394.05			0.013	0.010	2.984	0.6766					2.546E+003		A G ><
132	1,167.30			0.012	0.012	1.355	0.7030					6.481E+003		A G %
133	1,330.00			0.012	0.012	1.222	0.7055					3.353E+003		A G ><
134	708.80			0.012	0.012	1.950	0.6910					3.897E+003		A G ><
135	878.03			0.012	0.012	1.674	0.6963					4.122E+003		A G ><
136	252.80			0.012	0.012	4.183	0.6649					1.086E+003		A G ><
137	62.50			0.012	0.011	4.832	0.6571					4.722E+003		A G &
138	1,636.30			0.011	0.011	1.025	0.7096					5.248E+003		A G ><
139	991.49			0.011	0.011	1.532	0.6989					3.005E+003		A G ><
140	494.20			0.011	0.011	2.524	0.6821					4.081E+003		A G ><
141	528.00			0.011	0.011	2.406	0.6839					4.587E+003		A G ><
142	1,813.73			0.011	0.010	0.931	0.7119					2.186E+003		A G ><
143	2,880.30			0.010	0.010	0.553	0.6591							A G =
144	961.61			0.010	0.010	1.567	0.6982					4.741E+003		A G ><
145	1,118.90			0.010	0.010	1.400	0.7019					8.127E+003		A G ><
146	76.86			0.426	0.426	5.983	0.6523					6.723E+001		A X ><
147	79.29			0.710	0.710	6.107	0.6520					3.082E+001		A X ><
148	89.81			0.244	0.244	6.461	0.6506					1.190E+002		A X %
149	92.62			0.076	0.076	6.514	0.6502					5.117E+002		A X

Pb-210

Activity: < MDA **CL: 8.527E+000 Bq/kg** **MDA: 1.720E+001 Bq/kg**

N°	Energy [keV]	Peakenergy [keV]	ΔE [keV]	Emission [%] uncorr.	Emission [%] corr.	Eff. [%]	GEO Corr.	Net Area [counts]	1-σ Count.	Peakactivity [Bq/kg] time of count	Peakactivity [Bq/kg] Decay corr.	MDA [Bq/kg]	FWHM [keV]	Info
1	46.54				4.252	2.502	0.6678					1.720E+001		K G

U-235D

Activity: < MDA **CL: 2.587E+000 Bq/kg** **MDA: 5.233E+000 Bq/kg**

N°	Energy [keV]	Peakenergy [keV]	ΔE [keV]	Emission [%] uncorr.	Emission [%] corr.	Eff. [%]	GEO Corr.	Net Area [counts]	1-σ Count.	Peakactivity [Bq/kg] time of count	Peakactivity [Bq/kg] Decay corr.	MDA [Bq/kg]	FWHM [keV]	Info
1	163.35				5.240	5.662	0.6552					5.233E+000		K G %
2	185.72			57.000	57.000	5.224	0.6578					8.863E-001		A G
3	143.77			10.940	10.940	6.060	0.6530					2.948E+000		A G ><
4	84.21			6.700	6.700	6.307	0.6513					6.376E+000		A G
5	205.32			5.020	5.020	4.876	0.6600					5.774E+000		A G ><
6	300.07			2.470	2.470	3.670	0.6696					1.956E+001		A G ><
7	302.65			2.200	2.200	3.646	0.6699					7.526E+000		A G ><
8	283.69			1.700	1.700	3.831	0.6680					3.195E+001		A G ><
9	109.13			2.350	2.350	6.581	0.6498					1.228E+001		A G ><

10	330.06			1.400	1.400	3.412	0.6719					3.277E+001		A G ><
11	202.12			1.080	1.080	4.930	0.6597					3.550E+001		A G ><
12	81.23			0.905	0.905	6.193	0.6517					3.260E+001		A G %
13	194.94			0.630	0.630	5.056	0.6587					4.123E+001		A G ><
14	58.57			0.480	0.480	4.370	0.6597					7.214E+001		A G ><
15	102.27			0.441	0.441	6.596	0.6493					6.416E+001		A G %
16	82.09			0.418	0.418	6.228	0.6516					2.744E+001		A G ><
17	182.62			0.390	0.390	5.283	0.6574					9.793E+001		A G ><
18	72.74			0.380	0.380	5.730	0.6530					9.465E+001		A G ><
19	46.35			0.223	0.223	2.467	0.6678					3.106E+002		A G
20	140.76			0.200	0.200	6.119	0.6526					5.911E+001		A G ><
21	260.19			0.188	0.188	4.092	0.6656					1.942E+002		A G ><
22	340.74			0.181	0.181	3.330	0.6727					2.271E+002		A G ><
23	357.12			0.175	0.175	3.214	0.6739					2.411E+002		A G ><
24	99.28			0.137	0.137	6.585	0.6491					1.730E+002		A G %
25	221.39			0.118	0.118	4.618	0.6616					3.629E+002		A G ><
26	255.77			0.112	0.112	4.146	0.6652					1.859E+002		A G ><
27	312.92			0.102	0.102	3.554	0.6706					4.437E+002		A G %
28	354.46			0.100	0.100	3.232	0.6737					3.724E+002		A G ><
29	96.84			0.110	0.110	6.567	0.6496					8.136E+001		A G ><
30	96.09			0.091	0.091	6.560	0.6497					2.076E+002		A G ><
31	150.94			0.090	0.090	5.915	0.6538					1.344E+002		A G ><
32	52.73			0.085	0.085	3.549	0.6628					6.553E+002		A G %
33	135.66			0.080	0.080	6.217	0.6521					2.421E+002		A G ><
34	54.51			0.110	0.110	3.816	0.6619					1.275E+002		A G ><
35	240.88			0.074	0.074	4.338	0.6638					4.736E+002		A G ><
36	77.34			0.073	0.073	6.009	0.6523					3.890E+002		A G ><
37	44.15			0.065	0.065	2.057	0.6699					3.536E+002		A G ><
38	273.14			0.060	0.060	3.944	0.6669					6.894E+002		A G ><
39	277.32			0.060	0.060	3.898	0.6673					7.782E+002		A G %
40	42.86			0.060	0.060	1.811	0.6719					9.368E+002		A G ><
41	124.91			0.059	0.059	6.403	0.6510					5.796E+002		A G ><
42	42.01			0.056	0.056	1.649	0.6719					8.867E+002		A G ><
43	246.83			0.055	0.055	4.259	0.6643					5.223E+002		A G %
44	275.40			0.080	0.080	3.919	0.6672					2.811E+002		A G ><
45	74.94			0.051	0.051	5.872	0.6526					7.491E+002		A G
46	379.30			0.050	0.050	3.070	0.6755					5.063E+002		A G ><
47	63.72			0.070	0.070	4.961	0.6571					5.993E+002		A G
48	243.08			0.048	0.048	4.308	0.6639					6.582E+002		A G ><
49	217.94			0.040	0.040	4.671	0.6613					1.159E+003		A G %
50	291.65			0.040	0.040	3.751	0.6689					9.529E+002		A G ><
51	387.84			0.040	0.040	3.019	0.6762					5.969E+002		A G ><
52	345.92			0.040	0.040	3.292	0.6730					1.550E+003		A G ><
53	390.27			0.040	0.040	3.005	0.6764					1.488E+003		A G %
54	233.50			0.038	0.038	4.440	0.6629					1.265E+003		A G %
55	327.13			0.038	0.038	3.435	0.6717					1.073E+003		A G ><
56	198.89			0.036	0.036	4.986	0.6592					1.121E+003		A G ><
57	407.81			0.036	0.036	2.908	0.6776					1.544E+003		A G ><
58	51.21			0.034	0.034	3.308	0.6638					5.028E+002		A G ><
59	183.48			0.034	0.034	5.266	0.6574					1.144E+003		A G ><
60	145.94			0.032	0.032	6.016	0.6532					7.493E+002		A G ><
61	115.45			0.030	0.030	6.529	0.6503					8.583E+002		A G %
62	100.84			0.030	0.030	6.592	0.6491					7.891E+002		A G %
63	57.19			0.030	0.030	4.190	0.6602					7.771E+002		A G ><
64	41.40			0.000	0.029	1.532	0.6728					7.530E+002		A G ><
65	215.28			0.029	0.029	4.713	0.6611					9.467E+002		A G ><
66	120.35			0.026	0.026	6.469	0.6507					1.247E+003		A G ><
67	134.03			0.025	0.025	6.247	0.6518					1.480E+003		A G %
68	74.15			0.024	0.024	5.823	0.6526					1.548E+003		A G &
69	116.82			0.023	0.023	6.513	0.6504					5.330E+002		A G ><
70	174.15			0.018	0.018	5.446	0.6565					2.460E+003		A G ><
71	64.45			0.018	0.018	5.036	0.6560					1.389E+003		A G ><
72	136.55			0.012	0.012	6.200	0.6521					1.428E+003		A G ><
73	144.39			0.012	0.012	6.047	0.6530					2.716E+003		A G ><
74	246.04			0.011	0.011	4.269	0.6643					3.936E+003		A G %
75	286.55			0.010	0.010	3.802	0.6682					2.127E+003		A G ><
76	93.35			5.800	5.800	6.526	0.6502					7.905E+000		A X &
77	89.95			4.570	4.570	6.465	0.6506					6.351E+000		A X %
78	105.55			2.060	2.060	6.595	0.6495					1.629E+001		A X %
79	90.89			1.260	1.260	6.484	0.6504					8.280E+000		A X ><
80	87.77			0.770	0.770	6.413	0.6509					3.961E+001		A X
81	95.87			0.590	0.590	6.558	0.6497					3.203E+001		A X ><
82	102.80			0.450	0.450	6.596	0.6493					6.287E+001		A X %
83	92.29			0.370	0.370	6.509	0.6502					1.052E+002		A X
84	108.36			0.210	0.210	6.585	0.6498					1.374E+002		A X ><
85	106.17			0.170	0.170	6.593	0.6496					1.829E+002		A X %
86	111.89			0.071	0.071	6.562	0.6501					4.326E+002		A X %

Th-227 **Activity: < MDA** **CL: 1.019E+000 Bq/kg** **MDA: 2.067E+000 Bq/kg**

N°	Energy [keV]	Peakenergy [keV]	ΔE [keV]	Emission [%] uncorr.	Emission [%] corr.	Eff. [%]	GEO Corr.	Net Area [counts]	1-σ Count.	Peakactivity [Bq/kg] time of count	Peakactivity [Bq/kg] Decay corr.	MDA [Bq/kg]	FWHM [keV]	Info
1	235.96				12.422	4.405	0.6631					2.067E+000		K G ><
2	50.13			8.200	7.350	3.130	0.6645					5.686E+000		A G %

3	256.23	6.800	6.795	4.140	0.6652	3.068E+000	A	G	><
4	329.85	2.900	2.906	3.414	0.6719	1.578E+001	A	G	%
5	299.98	2.160	2.166	3.671	0.6696	2.231E+001	A	G	><
6	289.59	1.900	1.900	3.771	0.6687	2.384E+001	A	G	><
7	79.69	1.900	1.798	6.126	0.6520	1.213E+001	A	G	><
8	286.09	1.700	1.859	3.806	0.6682	1.143E+001	A	G	><
9	93.88	1.480	1.382	6.533	0.6499	1.293E+001	A	G	><
10	210.62	1.220	1.176	4.788	0.6605	3.062E+001	A	G	><
11	304.50	1.120	1.145	3.629	0.6700	2.844E+001	A	G	><
12	334.37	1.110	1.119	3.378	0.6721	3.359E+001	A	G	><
13	254.63	0.690	0.674	4.160	0.6649	5.192E+001	A	G	><
14	113.11	0.190	0.541	6.551	0.6501	5.896E+001	A	G	%
15	272.91	0.500	0.500	3.946	0.6669	8.265E+001	A	G	><
16	312.69	0.500	0.500	3.556	0.6706	9.042E+001	A	G	%
17	314.85	0.480	0.483	3.537	0.6708	7.417E+001	A	G	%
18	234.76	0.440	0.431	4.422	0.6630	3.340E+001	A	G	><
19	250.27	0.440	0.430	4.215	0.6646	1.281E+002	A	G	%
20	296.50	0.430	0.421	3.704	0.6692	1.296E+002	A	G	&
21	49.82	0.420	0.420	3.078	0.6645	1.001E+002	A	G	%
22	342.55	0.340	0.342	3.317	0.6728	7.646E+001	A	G	><
23	206.08	0.250	0.241	4.863	0.6602	1.226E+002	A	G	><
24	204.14	0.220	0.214	4.896	0.6600	1.347E+002	A	G	><
25	43.77	0.208	0.208	1.985	0.6699	1.489E+002	A	G	><
26	62.45	0.200	0.175	4.827	0.6577	1.493E+002	A	G	><
27	117.20	0.194	0.183	6.509	0.6505	1.665E+002	A	G	%
28	113.11	0.190	0.187	6.551	0.6501	1.707E+002	A	G	%
29	281.42	0.174	0.170	3.855	0.6677	2.167E+002	A	G	><
30	204.98	0.160	0.156	4.881	0.6600	1.850E+002	A	G	><
31	141.42	0.116	0.116	6.106	0.6527	1.135E+002	A	G	><
32	252.50	0.108	0.104	4.186	0.6648	1.399E+002	A	G	><
33	350.54	0.107	0.107	3.259	0.6735	4.615E+002	A	G	><
34	218.90	0.107	0.107	4.656	0.6613	4.495E+002	A	G	%
35	262.87	0.105	0.103	4.061	0.6659	3.978E+002	A	G	><
36	61.44	0.088	0.087	4.714	0.6581	3.231E+002	A	G	><
37	100.27	0.082	0.082	6.590	0.6491	2.888E+002	A	G	%
38	212.70	0.019	0.066	4.754	0.6608	5.404E+002	A	G	><
39	96.03	0.068	0.061	6.560	0.6497	3.119E+002	A	G	><
40	292.41	0.064	0.063	3.743	0.6689	6.029E+002	A	G	><
41	68.74	0.057	0.051	5.429	0.6542	3.860E+002	A	G	><
42	43.80	0.054	0.047	1.991	0.6699	6.537E+002	A	G	><
43	279.80	0.053	0.054	3.872	0.6676	5.665E+002	A	G	><
44	44.22	0.052	0.052	2.070	0.6699	4.413E+002	A	G	><
45	362.63	0.050	0.050	3.176	0.6743	1.094E+003	A	G	><
46	219.00	0.049	0.048	4.655	0.6613	1.001E+003	A	G	%
47	285.52	0.043	0.043	3.812	0.6682	5.716E+002	A	G	><
48	284.24	0.039	0.039	3.825	0.6680	1.367E+003	A	G	><
49	184.65	0.035	0.034	5.244	0.6578	1.449E+003	A	G	&
50	314.75	0.034	0.034	3.538	0.6708	1.099E+003	A	G	%
51	134.60	0.033	0.030	6.236	0.6520	1.145E+003	A	G	><
52	319.24	0.031	0.031	3.500	0.6711	1.012E+003	A	G	><
53	270.56	0.028	0.027	3.972	0.6666	1.175E+003	A	G	><
54	41.93	0.028	0.024	1.633	0.6719	2.033E+003	A	G	><
55	75.01	0.026	0.026	5.877	0.6526	1.468E+003	A	G	
56	99.58	0.025	0.025	6.587	0.6491	9.477E+002	A	G	%
57	64.35	0.025	0.022	5.026	0.6559	1.224E+003	A	G	><
58	94.97	0.024	0.023	6.548	0.6499	6.800E+002	A	G	><
59	72.85	0.024	0.023	5.738	0.6530	1.587E+003	A	G	><
60	383.51	0.024	0.024	3.045	0.6759	1.132E+003	A	G	><
61	248.10	0.024	0.024	4.242	0.6644	5.244E+002	A	G	><
62	201.64	0.023	0.022	4.938	0.6597	1.713E+003	A	G	><
63	140.60	0.021	0.019	6.122	0.6526	6.277E+002	A	G	><
64	175.80	0.020	0.020	5.414	0.6568	1.316E+003	A	G	><
65	212.70	0.019	0.016	4.754	0.6608	2.190E+003	A	G	><
66	289.77	0.019	0.018	3.769	0.6687	2.509E+003	A	G	><
67	308.40	0.017	0.016	3.594	0.6704	2.163E+003	A	G	><
68	173.45	0.017	0.017	5.460	0.6563	2.572E+003	A	G	%
69	44.40	0.016	0.016	2.104	0.6699	1.429E+003	A	G	><
70	50.85	0.015	0.014	3.249	0.6640	1.041E+003	A	G	><
71	40.20	0.015	0.012	1.305	0.6734	5.351E+003	A	G	><
72	300.50	0.014	0.015	3.666	0.6696	3.309E+003	A	G	><
73	168.36	0.014	0.013	5.561	0.6558	2.303E+003	A	G	%
74	164.52	0.014	0.014	5.639	0.6553	1.615E+003	A	G	><
75	48.30	0.014	0.012	2.817	0.6661	1.824E+003	A	G	><
76	138.40	0.014	0.014	6.165	0.6524	1.896E+003	A	G	><
77	123.58	0.014	0.013	6.423	0.6509	1.923E+003	A	G	><
78	73.63	0.014	0.013	5.790	0.6530	2.759E+003	A	G	><
79	200.50	0.013	0.012	4.958	0.6595	2.154E+003	A	G	><
80	197.56	0.013	0.011	5.009	0.6592	3.583E+003	A	G	><
81	99.60	0.013	0.013	6.587	0.6491	1.822E+003	A	G	%
82	246.12	0.012	0.012	4.268	0.6643	3.659E+003	A	G	%
83	117.50	0.012	0.012	6.505	0.6505	2.548E+003	A	G	%
84	346.45	0.012	0.016	3.288	0.6731	2.336E+003	A	G	><
85	150.14	0.011	0.010	5.931	0.6537	1.731E+003	A	G	><
86	77.40	0.010	0.010	6.012	0.6523	2.821E+003	A	G	><

87	69.80			0.010	0.010	5.514	0.6536					3.713E+003		A G %
88	379.40			0.010	0.010	3.070	0.6755					2.532E+003		A G ><
89	324.88			0.010	0.010	3.454	0.6715					4.054E+003		A G ><
90	306.10			0.010	0.010	3.614	0.6701					3.931E+003		A G ><
91	59.60			0.010	0.010	4.498	0.6593					3.432E+003		A G ><
92	267.05			0.010	0.009	4.012	0.6663					4.062E+003		A G ><
93	88.47			2.960	2.960	6.431	0.6507					3.111E+000		A X ><
94	85.43			1.810	1.810	6.346	0.6513					2.136E+001		A X &
95	100.10			1.040	0.934	6.589	0.6491					2.534E+001		A X %
96	103.31			0.340	0.297	6.597	0.6493					9.524E+001		A X %

Ra-223 Activity: < MDA CL: 1.762E+000 Bq/kg MDA: 3.557E+000 Bq/kg

N°	Energy [keV]	Peakenergy [keV]	ΔE [keV]	Emission [%] uncorr.	corr.	Eff. [%]	GEO Corr.	Net Area [counts]	1-σ Count.	Peakactivity [Bq/kg] time of count	Decay corr.	MDA [Bq/kg]	FWHM [keV]	Info
1	269.46				14.230	3.985	0.6666					3.557E+000		K G %
2	154.21			5.840	5.840	5.849	0.6542					5.986E+000		A G ><
3	323.87			4.060	4.060	3.462	0.6715					9.745E+000		A G ><
4	144.27			3.360	3.360	6.050	0.6530					9.616E+000		A G ><
5	338.28			2.850	2.850	3.349	0.6725					1.355E+001		A G ><
6	445.03			1.280	1.280	2.725	0.6794					4.612E+001		A G ><
7	122.32			1.238	1.238	6.442	0.6508					1.385E+001		A G ><
8	158.63			0.713	0.713	5.758	0.6547					4.832E+001		A G %
9	83.78			24.500	24.500	6.292	0.6513					1.696E+000		A X &
10	81.07			14.860	14.860	6.187	0.6517					1.988E+000		A X %
11	94.85			8.500	8.500	6.546	0.6499					1.857E+000		A X ><
12	97.90			2.720	2.720	6.576	0.6494					9.524E+000		A X ><

Rn-219 Activity: < MDA CL: 8.037E-001 Bq/kg MDA: 1.642E+000 Bq/kg

N°	Energy [keV]	Peakenergy [keV]	ΔE [keV]	Emission [%] uncorr.	corr.	Eff. [%]	GEO Corr.	Net Area [counts]	1-σ Count.	Peakactivity [Bq/kg] time of count	Decay corr.	MDA [Bq/kg]	FWHM [keV]	Info
1	271.23				11.070	3.965	0.6667					1.642E+000		K G ><
2	401.81			6.750	6.750	2.940	0.6772					9.270E+000		G ><
3	79.29			0.900	0.900	6.107	0.6520					2.431E+001		A X ><
4	76.86			0.540	0.540	5.983	0.6523					5.304E+001		A X ><

Pb-211 Activity: < MDA CL: 5.708E+000 Bq/kg MDA: 1.157E+001 Bq/kg

N°	Energy [keV]	Peakenergy [keV]	ΔE [keV]	Emission [%] uncorr.	corr.	Eff. [%]	GEO Corr.	Net Area [counts]	1-σ Count.	Peakactivity [Bq/kg] time of count	Decay corr.	MDA [Bq/kg]	FWHM [keV]	Info
1	404.83				3.830	2.924	0.6773					1.157E+001		K G ><
2	831.98			3.500	3.500	1.740	0.6951					1.752E+001		G ><
3	427.15			1.810	1.810	2.809	0.6785					1.758E+001		A G ><
4	766.68			0.620	0.620	1.844	0.6931					8.787E+001		A G ><
5	704.67			0.470	0.470	1.958	0.6909					7.332E+001		A G ><

Bi-211 Activity: < MDA CL: 1.580E+000 Bq/kg MDA: 3.201E+000 Bq/kg

N°	Energy [keV]	Peakenergy [keV]	ΔE [keV]	Emission [%] uncorr.	corr.	Eff. [%]	GEO Corr.	Net Area [counts]	1-σ Count.	Peakactivity [Bq/kg] time of count	Decay corr.	MDA [Bq/kg]	FWHM [keV]	Info
1	351.03				13.000	3.256	0.6735					3.201E+000		K G ><
2	70.83			0.726	0.726	5.594	0.6532					1.679E+001		A X ><
3	72.87			1.225	1.225	5.739	0.6530					2.931E+001		A X ><
4	82.60			0.417	0.417	6.248	0.6515					3.008E+001		A X ><

Ac-228 Activity: < MDA CL: 1.377E+000 Bq/kg MDA: 2.796E+000 Bq/kg

N°	Energy [keV]	Peakenergy [keV]	ΔE [keV]	Emission [%] uncorr.	corr.	Eff. [%]	GEO Corr.	Net Area [counts]	1-σ Count.	Peakactivity [Bq/kg] time of count	Decay corr.	MDA [Bq/kg]	FWHM [keV]	Info
1	911.20	910.56	0.64	26.200	25.504	1.629	0.6970	133	56.72	1.647E+000	1.647E+000	2.796E+000	2.32	K G %
2	968.96	968.27	0.69	15.900	15.482	1.558	0.6983	113	55.02	2.418E+000	2.418E+000	4.088E+000	1.45	G ><
3	338.32	338.17	0.15	11.400	11.065	3.348	0.6725	107	41.88	1.425E+000	1.425E+000	2.906E+000	1.27	G ><
4	964.79	962.82	1.97	4.990	4.710	1.563	0.6982	118	51.83	8.246E+000	8.246E+000	1.408E+001	0.19	G ><
5	463.00	462.20	0.80	4.450	3.915	2.647	0.6804	30	123.60	1.473E+000	1.473E+000	8.794E+000	0.19	G ><
6	794.94	794.60	0.35	4.310	3.747	1.797	0.6942	76	35.84	5.809E+000	5.809E+000	9.812E+000	1.04	A G ><
7	209.25			3.970	3.685	4.810	0.6604					7.559E+000		A G ><
8	270.24	269.67	0.58	3.550	3.092	3.976	0.6666	6	836.28	2.431E-001	2.431E-001	9.929E+000	2.95	A G %
9	1,588.20	1,587.34	0.86	3.060	3.082	1.052	0.7089	61	48.61	9.900E+000	9.900E+000	1.430E+001	2.20	A G ><
10	328.00			3.040	2.650	3.428	0.6717					1.215E+001		A G ><
11	129.07	129.41	-0.35	2.500	2.148	6.335	0.6513	49	110.13	1.734E+000	1.734E+000	9.569E+000	0.68	A G ><
12	409.46	409.26	0.20	2.020	1.763	2.899	0.6776	81	44.94	7.873E+000	7.873E+000	1.713E+001	0.75	A G ><
13	835.70	835.62	0.09	1.700	1.540	1.734	0.6952	50	53.04	9.624E+000	9.624E+000	2.486E+001	1.80	A G ><
14	772.29	772.01	0.28	1.520	1.310	1.834	0.6934	58	47.53	1.240E+001	1.240E+001	2.817E+001	0.24	A G ><
15	1,630.62	1,631.43	-0.81	1.520	1.530	1.028	0.7095	10	143.33	3.327E+000	3.327E+000	2.359E+001	0.59	A G ><
16	99.50			1.260	1.260	6.586	0.6491					1.500E+001		A G %
17	755.31	755.72	-0.40	1.030	0.926	1.864	0.6928	27	101.95	8.058E+000	8.058E+000	4.009E+001	1.11	A G ><
18	840.37			0.970	0.843	1.727	0.6953					4.877E+001		A G ><
19	562.50	562.17	0.32	0.890	0.874	2.299	0.6856	14	217.10	3.637E+000	3.637E+000	3.908E+001	0.29	A G ><
20	904.20	903.77	0.43	0.780	0.686	1.639	0.6968	21	116.69	9.521E+000	9.521E+000	5.502E+001	1.14	A G ><
21	153.97	153.60	0.36	0.754	0.671	5.853	0.6541	31	174.91	3.861E+000	3.861E+000	3.395E+001	0.24	A G ><
22	726.88	727.07	-0.19	0.680	0.586	1.915	0.6917	85	31.20	3.859E+001	3.859E+001	5.729E+001	1.15	A G ><
23	1,580.53	1,581.90	-1.37	0.620	0.621	1.057	0.7088	63	51.82	5.049E+001	5.049E+001	8.602E+001	0.71	A G ><
24	830.48	830.40	0.08	0.610	0.516	1.742	0.6951	2	1,160.55	1.293E+000	1.293E+000	7.563E+001	0.54	A G ><
25	1,247.10			0.524	0.460	1.287	0.7044					1.078E+002		A G ><
26	1,501.59	1,503.32	-1.73	0.513	0.496	1.105	0.7078	5	291.23	4.968E+000	4.968E+000	7.151E+001	0.16	A G ><

27	508.95			0.510	0.491	2.471	0.6830					1.195E+002		A G ><
28	782.14			0.500	0.470	1.818	0.6937					8.091E+001		A G ><
29	57.75			0.470	0.408	4.265	0.6603					6.800E+001		A G ><
30	1,638.27	1,637.94	0.33	0.460	0.461	1.024	0.7096	2	736.22	2.020E+000	2.020E+000	7.517E+001	0.74	A G ><
31	340.97	341.14	-0.17	0.405	0.347	3.329	0.6727	19	229.70	8.086E+000	8.086E+000	9.249E+001	1.39	A G ><
32	332.37			0.370	0.324	3.394	0.6722					1.003E+002		A G ><
33	1,110.60	1,110.79	-0.19	0.311	0.459	1.408	0.7017	58	44.26	4.610E+001	4.610E+001	9.786E+001	1.41	A G ><
34	199.40			0.299	0.266	4.977	0.6595					1.004E+002		A G ><
35	958.59			0.290	0.291	1.570	0.6981					1.326E+002		A G ><
36	1,625.09			0.270	0.290	1.031	0.7094					1.247E+002		A G ><
37	278.80			0.235	0.186	3.882	0.6675					1.653E+002		A G ><
38	321.65			0.232	0.185	3.480	0.6711					1.734E+002		A G ><
39	478.40			0.224	0.175	2.584	0.6813					1.917E+002		A G ><
40	1,033.24	1,033.10	0.14	0.204	0.202	1.486	0.6999	29	75.90	5.071E+001	5.071E+001	1.914E+002	2.12	A G ><
41	546.45			0.199	0.157	2.347	0.6849					2.137E+002		A G ><
42	570.88			0.190	0.147	2.274	0.6860					2.483E+002		A G ><
43	701.74	701.87	-0.13	0.181	0.161	1.964	0.6908	30	89.25	4.875E+001	4.875E+001	2.159E+002	1.00	A G ><
44	1,557.13			0.173	0.169	1.071	0.7085					2.166E+002		A G ><
45	1,666.51	1,667.13	-0.61	0.173	0.207	1.008	0.7099	5	239.15	1.321E+001	1.321E+001	1.601E+002	3.18	A G ><
46	503.82	503.56	0.26	0.171	0.132	2.489	0.6827	93	77.98	1.428E+002	1.428E+002	3.688E+002	0.45	A G ><
47	145.84			0.169	0.149	6.018	0.6532					1.486E+002		A G ><
48	707.42	707.41	0.01	0.162	0.132	1.952	0.6910	43	61.29	8.487E+001	8.487E+001	2.555E+002	0.59	A G ><
49	572.10	571.49	0.61	0.156	0.131	2.271	0.6860	30	103.18	5.145E+001	5.145E+001	2.606E+002	1.91	A G ><
50	1,153.27	1,155.39	-2.12	0.148	0.148	1.368	0.7027	1	1,728.73	3.737E+000	3.737E+000	3.190E+002	0.17	A G ><
51	987.87			0.140	0.120	1.536	0.6988					3.316E+002		A G ><
52	1,065.17			0.135	0.407	1.453	0.7006					1.157E+002		A G ><
53	191.35			0.133	0.107	5.120	0.6583					2.440E+002		A G ><
54	523.13	523.36	-0.23	0.129	0.102	2.422	0.6837	22	154.77	4.443E+001	4.443E+001	3.392E+002	0.13	A G ><
55	440.45	440.50	-0.05	0.128	0.119	2.746	0.6793	19	177.56	2.891E+001	2.891E+001	2.594E+002	0.40	A G ><
56	1,095.67			0.126	0.131	1.422	0.7014					3.622E+002		A G %
57	583.39	583.23	0.16	0.120	0.096	2.239	0.6864	13	609.10	3.129E+001	3.129E+001	5.570E+002	1.44	A G %
58	100.41	100.13	0.28	0.114	0.091	6.591	0.6491	96	54.53	7.724E+001	7.724E+001	2.038E+002	1.55	A G %
59	204.03			0.114	0.099	4.897	0.6600					2.905E+002		A G ><
60	947.98	949.03	-1.05	0.111	0.095	1.583	0.6979	8	306.52	2.787E+001	2.787E+001	4.275E+002	1.11	A G ><
61	1,245.15	1,244.84	0.31	0.110	0.164	1.289	0.7044	31	78.86	7.585E+001	7.585E+001	2.920E+002	0.22	A G ><
62	1,287.77			0.109	0.095	1.255	0.7049					4.831E+002		A G ><
63	674.63	675.55	-0.92	0.105	0.081	2.019	0.6898	3	769.95	1.046E+001	1.046E+001	4.072E+002	0.66	A G ><
64	944.19	945.09	-0.90	0.100	0.102	1.588	0.6978	33	74.28	1.034E+002	1.034E+002	3.765E+002	1.98	A G ><
65	651.53	651.57	-0.04	0.094	0.076	2.070	0.6889	18	150.13	5.747E+001	5.747E+001	4.248E+002	0.88	A G ><
66	1,686.22			0.094	0.100	0.997	0.7102					3.660E+002		A G ><
67	1,887.13			0.094	0.097	0.896	0.7128					3.543E+002		A G ><
68	282.02	282.44	-0.42	0.090	0.071	3.848	0.6679	49	101.89	8.760E+001	8.760E+001	4.322E+002	1.09	A G ><
69	1,142.87			0.011	0.008	1.377	0.7025					5.796E+003		A G ><
70	389.32	389.54	-0.22	0.011	0.008	3.011	0.6763	25	152.55	5.298E+002	5.298E+002	4.100E+003	0.18	A G ><
71	1,700.62			0.010	0.010	0.989	0.7104					3.518E+003		A G ><
72	1,135.26	1,135.07	0.19	0.010	0.009	1.384	0.7023	32	79.50	1.346E+003	1.346E+003	5.159E+003	0.59	A G ><
73	114.56			0.010	0.008	6.538	0.6503					2.549E+003		A G %
74	1,103.43			0.010	0.009	1.415	0.7015					5.062E+003		A G ><
75	89.95	89.88	0.07	2.500	2.500	6.465	0.6506	307	37.46	9.105E+000	9.105E+000	1.122E+001	1.53	A X %
76	93.35	92.64	0.71	4.100	4.100	6.526	0.6502	2,500	6.72	4.475E+001	4.475E+001	9.528E+000	1.05	A X <>
77	105.55			1.500	1.500	6.595	0.6495					1.281E+001		A X %

Warning: Some peaks of this nuclide are identified but not all that are marked as keylines - no calcualtion of nuclide activity!

Ra-224

Activity: < MDA **CL: 4.208E+000 Bq/kg** **MDA: 8.513E+000 Bq/kg**

N°	Energy [keV]	Peakenergy [keV]	ΔE [keV]	Emission [%] uncorr.	Emission [%] corr.	Eff. [%]	GEO Corr.	Net Area [counts]	1-σ Count.	Peakactivity [Bq/kg] time of count	Peakactivity [Bq/kg] Decay corr.	MDA [Bq/kg]	FWHM [keV]	Info
1	240.99				4.119	4.336	0.6638					8.513E+000		K G ><
2	83.78			0.214	0.214	6.292	0.6513					1.941E+002		A X &
3	81.07			0.130	0.130	6.187	0.6517					2.272E+002		A X %

Pb-212

Activity: < MDA **CL: 5.672E-001 Bq/kg** **MDA: 1.144E+000 Bq/kg**

N°	Energy [keV]	Peakenergy [keV]	ΔE [keV]	Emission [%] uncorr.	Emission [%] corr.	Eff. [%]	GEO Corr.	Net Area [counts]	1-σ Count.	Peakactivity [Bq/kg] time of count	Peakactivity [Bq/kg] Decay corr.	MDA [Bq/kg]	FWHM [keV]	Info
1	238.63				43.596	4.368	0.6634					1.144E+000		K G
2	300.09			3.180	2.931	3.670	0.6696					1.649E+001		G ><
3	115.18			0.624	0.559	6.531	0.6503					4.606E+001		G %
4	415.27			0.144	0.198	2.869	0.6779					2.556E+002		G ><
5	176.64			0.052	0.047	5.398	0.6568					5.028E+002		G ><
6	123.45			0.052	0.048	6.425	0.6509					4.116E+002		A G ><
7	74.82			10.070	10.070	5.865	0.6526					3.799E+000		A X
8	77.11			16.900	16.900	5.997	0.6523					1.691E+000		A X ><
9	87.35			5.770	5.770	6.402	0.6509					5.295E+000		A X
10	90.08			1.770	1.770	6.467	0.6506					1.639E+001		A X %

Bi-212

Activity: < MDA **CL: 4.070E+000 Bq/kg** **MDA: 8.276E+000 Bq/kg**

N°	Energy [keV]	Peakenergy [keV]	ΔE [keV]	Emission [%] uncorr.	Emission [%] corr.	Eff. [%]	GEO Corr.	Net Area [counts]	1-σ Count.	Peakactivity [Bq/kg] time of count	Peakactivity [Bq/kg] Decay corr.	MDA [Bq/kg]	FWHM [keV]	Info
1	727.33				6.426	1.914	0.6917					8.276E+000		K G ><
2	1,620.74			1.510	1.526	1.033	0.7093					4.729E+001		A G ><
3	785.37			1.110	0.992	1.813	0.6939					1.495E+001		A G ><
4	1,078.63			0.550	0.492	1.439	0.7009					1.582E+002		A G ><
5	893.41			0.380	0.340	1.653	0.6966					1.036E+002		A G ><

6	452.98			0.340	0.340	2.690	0.6799					1.299E+002		A G	><

Ac-228 **Activity:** **< MDA** **CL: 1.377E+000 Bq/kg** **MDA: 2.796E+000 Bq/kg**

N°	Energy [keV]	Peakenergy [keV]	ΔE [keV]	Emission [%] uncorr.	Emission [%] corr.	Eff. [%]	GEO Corr.	Net Area [counts]	1-σ Count.	Peakactivity [Bq/kg] time of count	Peakactivity [Bq/kg] Decay corr.	MDA [Bq/kg]	FWHM [keV]	Info	
1	616.21	615.87	0.34	0.084	0.073	2.153	0.6876	81	35.83	2.611E+002	2.611E+002	4.525E+002	0.21	K G	><
2	620.32			0.084	0.072	2.143	0.6877					4.509E+002		A G	><
3	792.69			0.081	0.074	1.801	0.6941					5.282E+002		A G	><
4	988.65			0.081	0.077	1.535	0.6989					5.208E+002		A G	><
5	520.16			0.070	0.055	2.432	0.6836					6.254E+002		A G	><
6	688.12			0.070	0.066	1.991	0.6902					5.036E+002		A G	%
7	1,164.55	1,164.79	-0.24	0.067	0.056	1.358	0.7030	2	1,421.78	1.188E+001	1.188E+001	8.340E+002	N/A	A G	><
8	666.45	664.90	1.55	0.065	0.053	2.037	0.6894	17	155.67	8.118E+001	8.118E+001	6.327E+002	0.96	A G	><
9	1,250.06			0.065	0.059	1.285	0.7044					8.108E+002		A G	><
10	677.08			0.000	0.060	2.014	0.6899					5.760E+002		A G	><
11	774.07			0.062	0.063	1.831	0.6935					6.175E+002		A G	><
12	1,952.37	1,951.34	1.03	0.062	0.064	0.867	0.7137	14	77.95	1.356E+002	1.356E+002	5.154E+002	0.42	A G	><
13	1,117.65			0.061	0.062	1.401	0.7018					8.462E+002		A G	><
14	223.79			0.058	0.045	4.581	0.6619					6.446E+002		A G	><
15	1,677.66	1,678.27	-0.61	0.057	0.058	1.002	0.7101	1	1,165.07	9.710E+000	9.710E+000	5.809E+002	0.50	A G	><
16	640.32	641.18	-0.86	0.057	0.048	2.095	0.6885	13	204.58	6.676E+001	6.676E+001	6.745E+002	0.19	A G	><
17	1,039.83			0.056	0.085	1.479	0.7000					4.879E+002		A G	><
18	1,702.40	1,702.95	-0.55	0.055	0.057	0.988	0.7104	20	66.73	1.839E+002	1.839E+002	5.954E+002	0.90	A G	><
19	184.55	184.34	0.21	0.054	0.054	5.246	0.6576	36	178.82	6.140E+001	6.140E+001	3.752E+002	0.17	A G	><
20	824.93	826.31	-1.38	0.053	0.044	1.750	0.6950	30	89.75	2.004E+002	2.004E+002	8.893E+002	1.71	A G	><
21	975.98	973.91	2.07	0.052	0.047	1.550	0.6985	104	63.39	7.338E+002	7.338E+002	1.537E+003	0.38	A G	><
22	1,286.29	1,285.50	0.79	0.052	0.058	1.256	0.7049	12	185.43	8.314E+001	8.314E+001	7.749E+002	1.30	A G	><
23	515.12	515.46	-0.34	0.051	0.040	2.449	0.6833	1	2,305.39	7.295E+000	7.295E+000	6.014E+002	N/A	A G	><
24	449.11	447.46	1.65	0.050	0.040	2.707	0.6796	48	69.52	2.188E+002	2.188E+002	7.585E+002	0.55	A G	><
25	874.45			0.050	0.052	1.679	0.6961					7.343E+002		A G	><
26	1,537.89	1,537.84	0.05	0.049	0.049	1.082	0.7082	11	136.19	1.100E+002	1.100E+002	7.394E+002	3.44	A G	%
27	555.07			0.048	0.042	2.321	0.6853					8.079E+002		A G	><
28	1,040.94	1,041.55	-0.61	0.047	0.043	1.478	0.7001	30	78.29	2.424E+002	2.424E+002	9.443E+002	0.72	A G	><
29	629.41	629.31	0.10	0.047	0.047	2.121	0.6881	10	267.82	5.082E+001	5.082E+001	6.738E+002	0.15	A G	><
30	870.47	872.79	-2.32	0.046	0.041	1.684	0.6961	42	60.47	3.085E+002	3.085E+002	8.958E+002	1.03	A G	><
31	1,823.22			0.046	0.045	0.927	0.7120					7.984E+002		A G	><
32	141.00			0.000	0.036	6.114	0.6526					6.307E+002		A G	><
33	263.58	263.37	0.21	0.043	0.033	4.052	0.6659	6	804.04	2.318E+001	2.318E+001	9.102E+002	0.35	A G	><
34	649.02	648.95	0.07	0.042	0.035	2.075	0.6888	90	31.29	6.344E+002	6.344E+002	9.319E+002	0.75	A G	><
35	1,548.65			0.040	0.041	1.076	0.7083					8.656E+002		A G	><
36	737.74			0.039	0.035	1.895	0.6921					1.086E+003		A G	><
37	698.99	699.51	-0.52	0.038	0.038	1.969	0.6907	37	72.37	2.487E+002	2.487E+002	8.883E+002	0.26	A G	><
38	1,835.29			0.038	0.040	0.921	0.7121					9.610E+002		A G	><
39	1,430.99			0.037	0.036	1.151	0.7068					1.033E+003		A G	><
40	1,842.15	1,841.35	0.80	0.037	0.037	0.918	0.7122	14	85.89	2.197E+002	2.197E+002	9.378E+002	0.06	A G	><
41	1,758.11			0.036	0.035	0.959	0.7111					1.037E+003		A G	><
42	173.96	173.99	-0.03	0.036	0.029	5.450	0.6565	75	71.52	2.303E+002	2.303E+002	8.424E+002	0.38	A G	><
43	326.04			0.035	0.035	3.444	0.6716					9.369E+002		A G	><
44	1,573.39	1,574.12	-0.73	0.034	0.033	1.061	0.7087	3	527.97	3.964E+001	3.964E+001	1.054E+003	1.91	A G	><
45	471.77			0.034	0.034	2.611	0.6810					1.025E+003		A G	%
46	873.10	872.87	0.23	0.032	0.033	1.680	0.6961	32	78.97	3.031E+002	3.031E+002	1.159E+003	1.04	A G	><
47	214.89			0.031	0.031	4.719	0.6611					9.181E+002		A G	><
48	399.83			0.031	0.025	2.951	0.6771					1.329E+003		A G	><
49	816.82	816.43	0.39	0.031	0.050	1.763	0.6948	3	882.41	1.771E+001	1.771E+001	7.870E+002	2.52	A G	%
50	1,017.94			0.030	0.027	1.503	0.6995					1.554E+003		A G	><
51	1,724.19			0.030	0.048	0.977	0.7107					7.062E+002		A G	><
52	397.95			0.029	0.021	2.962	0.6769					1.528E+003		A G	><
53	887.26	887.03	0.23	0.029	0.023	1.661	0.6964	40	64.37	5.340E+002	5.340E+002	1.654E+003	0.66	A G	><
54	663.88	663.09	0.79	0.029	0.025	2.042	0.6893	2	1,588.27	1.689E+001	1.689E+001	1.357E+003	0.50	A G	><
55	257.48			0.029	0.029	4.125	0.6654					1.052E+003		A G	%
56	979.49	978.98	0.51	0.028	0.029	1.546	0.6986	40	56.27	4.598E+002	4.598E+002	1.255E+003	1.44	A G	><
57	137.94			0.028	0.028	6.174	0.6522					7.749E+002		A G	><
58	919.03	918.35	0.68	0.028	0.025	1.619	0.6972	28	88.00	3.610E+002	3.610E+002	1.564E+003	0.52	A G	><
59	671.95			0.027	0.024	2.025	0.6897					1.361E+003		A G	><
60	77.34	77.09	0.25	0.027	0.023	6.009	0.6523	376	27.67	1.286E+003	1.286E+003	1.165E+003	0.88	A G	><
61	540.67			0.027	0.022	2.365	0.6847					1.543E+003		A G	><
62	377.99			0.026	0.021	3.078	0.6754					1.580E+003		A G	><
63	231.42	231.18	0.24	0.026	0.026	4.469	0.6626	77	72.95	3.221E+002	3.221E+002	1.108E+003	0.23	A G	><
64	1,870.82	1,871.73	-0.91	0.026	0.026	0.904	0.7126	25	50.39	5.489E+002	5.489E+002	1.326E+003	0.31	A G	><
65	492.29			0.025	0.019	2.531	0.6820					1.770E+003		A G	><
66	1,175.33			0.025	0.024	1.348	0.7032					2.000E+003		A G	%
67	548.73			0.024	0.021	2.340	0.6852					1.600E+003		A G	><
68	135.51			0.024	0.020	6.220	0.6520					1.053E+003		A G	><
69	610.65	611.10	-0.45	0.024	0.024	2.167	0.6874	103	52.56	1.004E+003	1.004E+003	1.739E+003	0.14	A G	><
70	791.43	791.48	-0.05	0.024	0.020	1.803	0.6941	41	65.65	5.800E+002	5.800E+002	1.839E+003	0.56	A G	><
71	474.79	474.63	0.16	0.023	0.018	2.599	0.6811	38	95.01	4.082E+002	4.082E+002	1.867E+003	0.16	A G	><
72	1,965.22	1,965.37	-0.15	0.022	0.023	0.861	0.7139	8	140.45	2.154E+002	2.154E+002	1.515E+003	0.15	A G	><
73	1,019.88	1,019.99	-0.11	0.022	0.019	1.500	0.6996	7	339.70	1.257E+002	1.257E+002	2.171E+003	0.12	A G	><
74	1,415.55	1,415.49	0.06	0.022	0.019	1.161	0.7066	8	207.52	1.759E+002	1.759E+002	1.869E+003	0.41	A G	><
75	1,217.03			0.022	0.018	1.312	0.7040					2.831E+003		A G	><
76	419.38			0.022	0.017	2.848	0.6782					1.818E+003		A G	><
77	1,357.81	1,355.81	2.00	0.021	0.019	1.202	0.7058	3	651.05	6.258E+001	6.258E+001	2.076E+003	0.68	A G	><
78	1,560.02			0.021	0.022	1.069	0.7086					1.685E+003		A G	><

N°	Energy [keV]	Peakenergy [keV]	ΔE [keV]	Emission [%] uncorr.	Emission [%] corr.	Eff. [%]	GEO Corr.	Net Area [counts]	1-σ Count.	Peakactivity [Bq/kg] time of count	Peakactivity [Bq/kg] Decay corr.	MDA [Bq/kg]	FWHM [keV]	Info
79	1,469.74	1,469.58	0.16	0.021	0.018	1.125	0.7073	32	48.90	7.941E+002	7.941E+002	1.811E+003	0.25	A G ><
80	1,929.78			0.021	0.021	0.877	0.7134					1.689E+003		A G ><
81	56.88			0.020	0.020	4.149	0.6606					1.412E+003		A G ><
82	1,309.76			0.020	0.019	1.238	0.7052					2.259E+003		A G ><
83	776.51			0.020	0.016	1.827	0.6936					2.517E+003		A G ><
84	452.50			0.020	0.016	2.692	0.6799					1.957E+003		A G ><
85	1,374.24	1,376.26	-2.02	0.020	0.358	1.190	0.7061	4	426.23	5.187E+000	5.187E+000	1.123E+002	0.40	A G ><
86	1,054.13	1,055.10	-0.97	0.019	0.022	1.464	0.7004	24	102.80	3.712E+002	3.712E+002	1.912E+003	2.68	A G %
87	1,016.44	1,015.04	1.40	0.019	0.018	1.504	0.6994	20	124.08	3.821E+002	3.821E+002	2.384E+003	4.20	A G %
88	718.30			0.019	0.015	1.931	0.6914					2.311E+003		A G ><
89	1,738.46			0.018	0.018	0.969	0.7109					2.011E+003		A G ><
90	356.70			0.018	0.016	3.216	0.6739					2.009E+003		A G ><
91	901.38			0.017	0.015	1.642	0.6968					2.524E+003		A G ><
92	1,347.50			0.016	0.015	1.210	0.7057					2.674E+003		A G ><
93	457.18	456.91	0.27	0.016	0.012	2.672	0.6802	109	34.32	1.649E+003	1.649E+003	2.656E+003	0.66	A G ><
94	921.87			0.015	0.023	1.616	0.6973					1.649E+003		A G ><
95	626.80	625.90	0.90	0.015	0.013	2.128	0.6880	16	171.75	2.966E+002	2.966E+002	2.511E+003	0.29	A G ><
96	1,315.33			0.015	0.017	1.233	0.7053					2.607E+003		A G ><
97	1,276.72	1,278.27	-1.55	0.015	0.013	1.263	0.7048	21	101.91	6.911E+002	6.911E+002	3.460E+003	0.35	A G ><
98	470.21	470.31	-0.10	0.014	0.011	2.617	0.6809	24	151.66	4.129E+002	4.129E+002	3.031E+003	0.75	A G ><
99	1,053.11	1,052.62	0.49	0.014	0.013	1.465	0.7003	4	556.02	1.207E+002	1.207E+002	3.419E+003	0.14	A G ><
100	877.38	876.93	0.45	0.014	0.012	1.675	0.6962	19	130.46	4.663E+002	4.663E+002	2.975E+003	0.24	A G ><
101	1,365.71	1,363.95	1.76	0.014	0.012	1.196	0.7059	7	266.01	2.335E+002	2.335E+002	3.144E+003	0.77	A G ><
102	415.96	415.00	0.96	0.014	0.010	2.865	0.6779	46	77.86	8.371E+002	8.371E+002	3.197E+003	1.07	A G ><
103	168.53	168.27	0.26	0.014	0.012	5.558	0.6558	65	83.29	4.791E+002	4.791E+002	1.994E+003	1.46	A G %
104	1,401.52			0.013	0.011	1.171	0.7064					3.387E+003		A G ><
105	1,907.14			0.012	0.012	0.887	0.7131					2.816E+003		A G %
106	853.96	853.59	0.37	0.012	0.012	1.707	0.6957	47	54.94	1.147E+003	1.147E+003	3.072E+003	0.90	A G ><
107	623.48			0.012	0.011	2.136	0.6878					2.965E+003		A G ><
108	490.33			0.012	0.012	2.538	0.6820					2.977E+003		A G ><
109	1,451.43			0.011	0.011	1.137	0.7071					3.731E+003		A G ><
110	1,062.57			0.011	0.009	1.455	0.7006					5.173E+003		A G ><
111	1,740.50			0.011	0.012	0.968	0.7109					3.019E+003		A G ><
112	1,074.73			0.011	0.009	1.443	0.7009					5.029E+003		A G ><

Warning: Some peaks of this nuclide are identified but not all that are marked as keylines - no calcualtion of nuclide activity!

Bi-212

Activity: < MDA **CL: 4.070E+000 Bq/kg** **MDA: 8.276E+000 Bq/kg**

N°	Energy [keV]	Peakenergy [keV]	ΔE [keV]	Emission [%] uncorr.	Emission [%] corr.	Eff. [%]	GEO Corr.	Net Area [counts]	1-σ Count.	Peakactivity [Bq/kg] time of count	Peakactivity [Bq/kg] Decay corr.	MDA [Bq/kg]	FWHM [keV]	Info
1	288.18				0.320	3.785	0.6684					1.161E+002		K G ><
2	1,512.70			0.290	0.341	1.098	0.7079					1.301E+002		A G ><
3	952.12			0.140	0.125	1.578	0.6980					2.548E+002		A G ><
4	328.04			0.121	0.121	3.428	0.6717					3.375E+002		A G ><
5	1,805.96			0.120	0.143	0.935	0.7118					2.009E+002		A G ><
6	1,679.45			0.070	0.076	1.001	0.7101					1.529E+002		A G ><
7	473.40			0.044	0.044	2.604	0.6810					9.412E+002		A G ><
8	492.84			0.039	0.039	2.529	0.6821					1.146E+003		A G ><
9	1,073.60			0.015	0.014	1.444	0.7008					1.399E+003		A G ><
10	433.50			0.011	0.011	2.779	0.6789					3.653E+003		A G ><
11	70.83			0.052	0.052	5.594	0.6532					2.322E+002		A X ><
12	72.87			0.089	0.089	5.739	0.6530					4.035E+002		A X ><
13	76.86			0.039	0.039	5.983	0.6523					7.381E+002		A X ><
14	79.29			0.065	0.065	6.107	0.6520					3.382E+002		A X ><
15	82.60			0.030	0.030	6.248	0.6515					4.167E+002		A X ><
16	89.81			0.022	0.022	6.461	0.6506					1.302E+003		A X %

Tl-208

Activity: < MDA **CL: 3.721E-001 Bq/kg** **MDA: 7.545E-001 Bq/kg**

N°	Energy [keV]	Peakenergy [keV]	ΔE [keV]	Emission [%] uncorr.	Emission [%] corr.	Eff. [%]	GEO Corr.	Net Area [counts]	1-σ Count.	Peakactivity [Bq/kg] time of count	Peakactivity [Bq/kg] Decay corr.	MDA [Bq/kg]	FWHM [keV]	Info
1	583.19				74.437	2.240	0.6864					7.545E-001		K G
2	860.53			12.400	11.905	1.698	0.6958					3.484E+000		G ><
3	2,614.51			99.755	85.130	0.626	0.6591							G =
4	510.74			22.500	18.305	2.464	0.6830					1.176E+001		A G @
5	277.37			6.600	5.375	3.898	0.6673					8.645E+000		A G %
6	763.45			1.800	1.531	1.850	0.6931					3.287E+001		A G ><
7	252.71			0.780	0.567	4.184	0.6648					2.235E+001		A G ><
8	1,093.90			0.440	1.718	1.424	0.7013					3.270E+001		A G ><
9	233.37			0.310	0.241	4.442	0.6629					1.997E+002		A G %
10	722.26			0.240	0.211	1.924	0.6916					2.394E+002		A G ><
11	982.70			0.204	0.168	1.542	0.6987					2.593E+002		A G ><
12	211.52			0.180	0.180	4.773	0.6605					2.007E+002		A G ><
13	927.64			0.128	0.106	1.608	0.6974					4.909E+002		A G ><
14	835.90			0.076	0.060	1.734	0.6952					7.684E+002		A G ><
15	588.11			0.060	0.044	2.226	0.6865					1.193E+003		A G ><
16	1,283.04			0.052	0.043	1.258	0.7049					2.005E+003		A G ><
17	486.08			0.049	0.039	2.555	0.6818					1.119E+003		A G ><
18	748.87			0.046	0.038	1.875	0.6925					1.230E+003		A G ><
19	821.48			0.041	0.032	1.755	0.6949					1.214E+003		A G ><
20	650.27			0.041	0.032	2.073	0.6889					1.210E+003		A G ><
21	883.59			0.031	0.031	1.666	0.6964					9.982E+002		A G ><
22	808.32			0.030	0.024	1.776	0.6946					1.257E+003		A G ><
23	705.34			0.022	0.017	1.956	0.6908					1.998E+003		A G ><

N°	Energy [keV]	Peakenergy [keV]	ΔE [keV]	Emission [%] uncorr.	Emission [%] corr.	Eff. [%]	GEO Corr.	Net Area [counts]	1-σ Count.	Peakactivity [Bq/kg] time of count	Peakactivity [Bq/kg] Decay corr.	MDA [Bq/kg]	FWHM [keV]	Info
24	1,185.57			0.017	0.017	1.339	0.7035					2.111E+003		A G ><
25	1,160.96			0.011	0.010	1.361	0.7029					5.755E+003		A G ><
26	74.97			3.420	3.420	5.874	0.6526					1.117E+001		A X
27	72.80			2.030	2.030	5.735	0.6530					1.770E+001		A X ><
28	84.95			1.170	1.170	6.331	0.6513					3.527E+001		A X
29	87.58			0.353	0.353	6.408	0.6509					8.647E+001		A X

Cs-137

Activity: < MDA **CL: 1.763E-001 Bq/kg** **MDA: 3.615E-001 Bq/kg**

N°	Energy [keV]	Peakenergy [keV]	ΔE [keV]	Emission [%] uncorr.	Emission [%] corr.	Eff. [%]	GEO Corr.	Net Area [counts]	1-σ Count.	Peakactivity [Bq/kg] time of count	Peakactivity [Bq/kg] Decay corr.	MDA [Bq/kg]	FWHM [keV]	Info
1	661.66				84.990	2.047	0.6893					3.615E-001		K G ><

Annihil

Activity: < MDA **CL: 5.283E-001 Bq/kg** **MDA: 1.063E+000 Bq/kg**

N°	Energy [keV]	Peakenergy [keV]	ΔE [keV]	Emission [%] uncorr.	Emission [%] corr.	Eff. [%]	GEO Corr.	Net Area [counts]	1-σ Count.	Peakactivity [Bq/kg] time of count	Peakactivity [Bq/kg] Decay corr.	MDA [Bq/kg]	FWHM [keV]	Info
1	511.00				200.000	2.463	0.6830					1.063E+000		K P @

Library Peak Codes:

G - Gamma Ray
X - X-Ray
P - Positron Decay
S - Single-Escape
D - Double-Escape
K - Key Line
A - Not in Average

Analysis Codes:

<> - Peak is too wide, but only one peak in library.
! - Peak is part of a multiplet and this area went negative during deconvolution.
>< - Peak is too narrow.
@ - Peak is too wide at FW25M, but ok at FWHM.
% - Peak fails sensitivity test.
$ - Peak identified, but first peak of this nuclide failed one or more qualification tests.
+ - Peak activity higher than counting uncertainty range.
- - Peak activity lower than counting uncertainty range.
= - Peak outside analysis energy range.
& - Calculated peak centroid is not close enough to the library energy centroid for positive identification.
} - Peak is too close to another for the activity to be found directly.
(= This peak was used in the nuclide activity average

Acquired, Analyzed & Saved by:	Reviewed & Approved by:
LVis Admin (D) @ GAMMA-SPEC	-
