# Supplementary material for: Yellow, Red, and Black Lepidium meyenii Preparations Differentially Modulate Doxorubicin Response and Lipid Metabolism-Related Changes in Pancreatic Cancer Cells
Source: Molecules. 2026 Nov 6;31(21):4011. doi: 10.3390/molecules31214011 (PMC13765015; doi:10.3390/molecules31214011)
Supplement: Supplementary file 1 [file molecules-31-04011-s001.zip › molecules-4589649-supplementary.pdf]

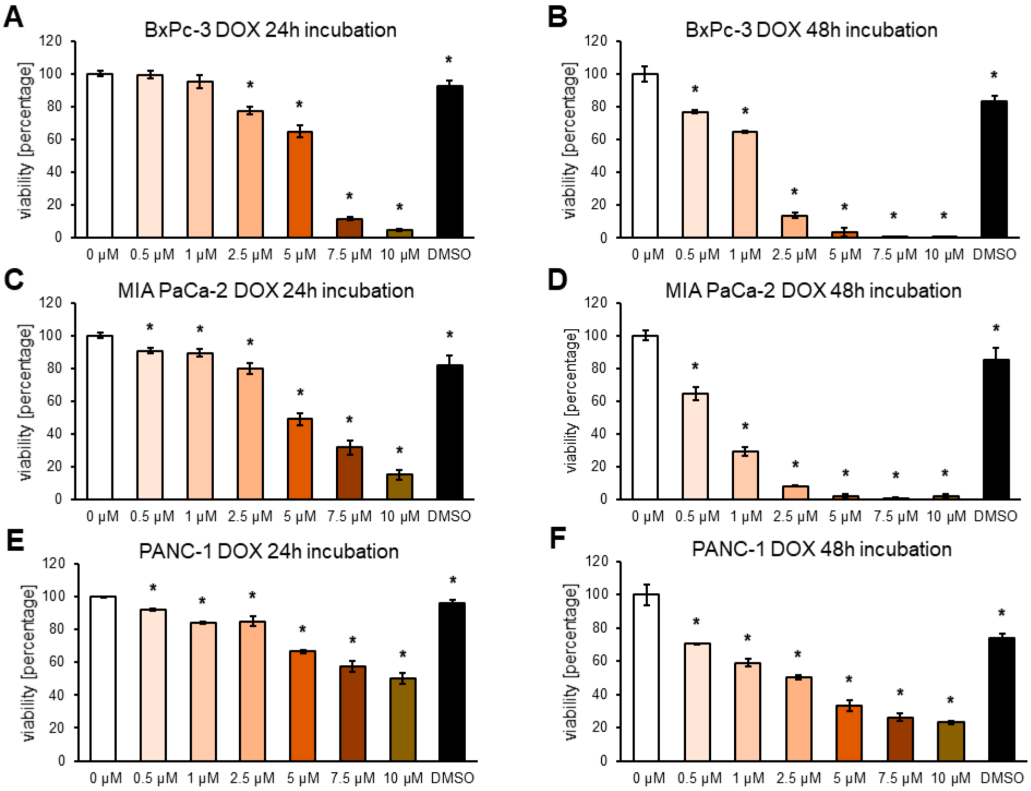

Supplementary Figure S1. Effects of doxorubicin (DOX) on pancreatic cancer cell viability. BxPC-3 cells were treated with increasing concentrations of DOX for 24 h (A) or 48 h (B). MIA PaCa-2 cells were treated for 24 h (C) or 48 h (D). PANC-1 cells were treated for 24 h (E) or 48 h (F). Cell viability was assessed following treatment. As a vehicle control, DMSO was used at the same concentration as that present in the highest DOX dose. Data are presented as mean ± SD from three independent biological experiments, each performed with four technical replicates (n = 3 biological experiments). * *$p < 0.05$ vs. 0 µM DOX*.

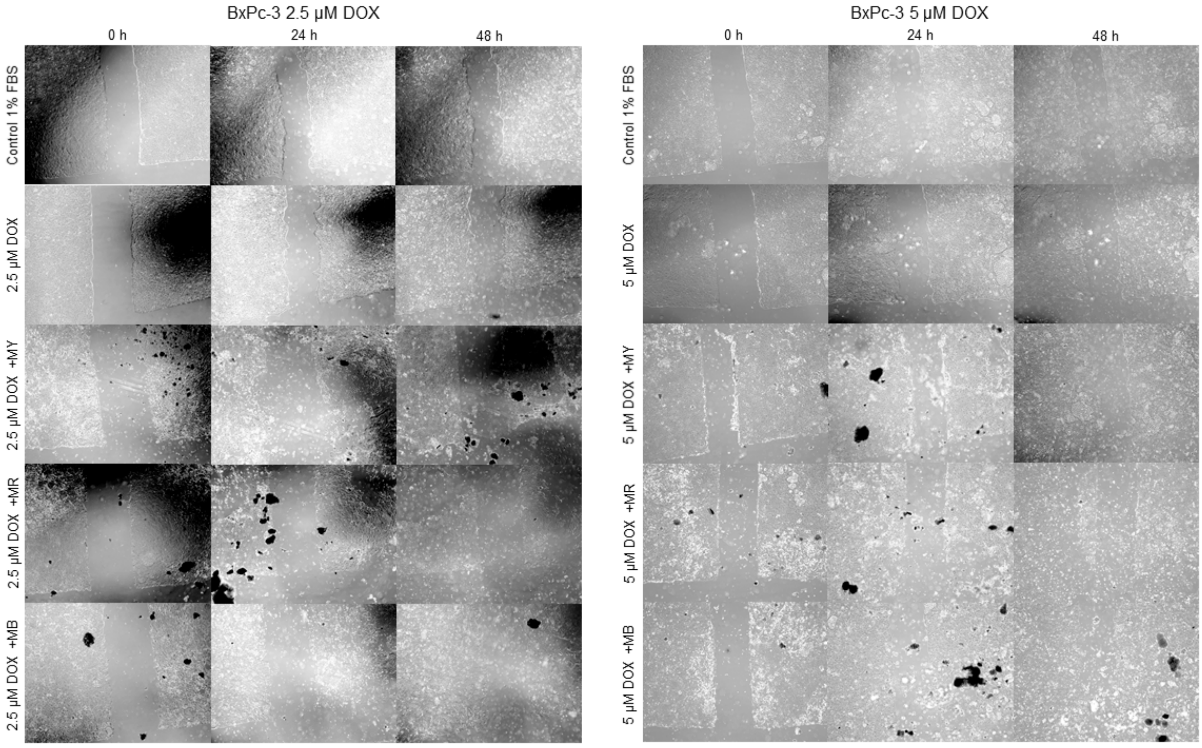

Supplementary Figure S2. Representative microscopy images of the wound healing assay in BxPC-3 pancreatic cancer cells following doxorubicin (DOX) and maca co-treatment. Images show wound closure at 0, 24, and 48 h following treatment with (A) 2.5 μM DOX or (B) 5 μM DOX alone or in combination with Maca Yellow (MY), Maca Red (MR), or Maca Black (MB) (100 μg/mL). Untreated cells served as the control. Representative micrographs illustrate treatment-dependent changes in wound closure compared to DOX monotherapy and control conditions.

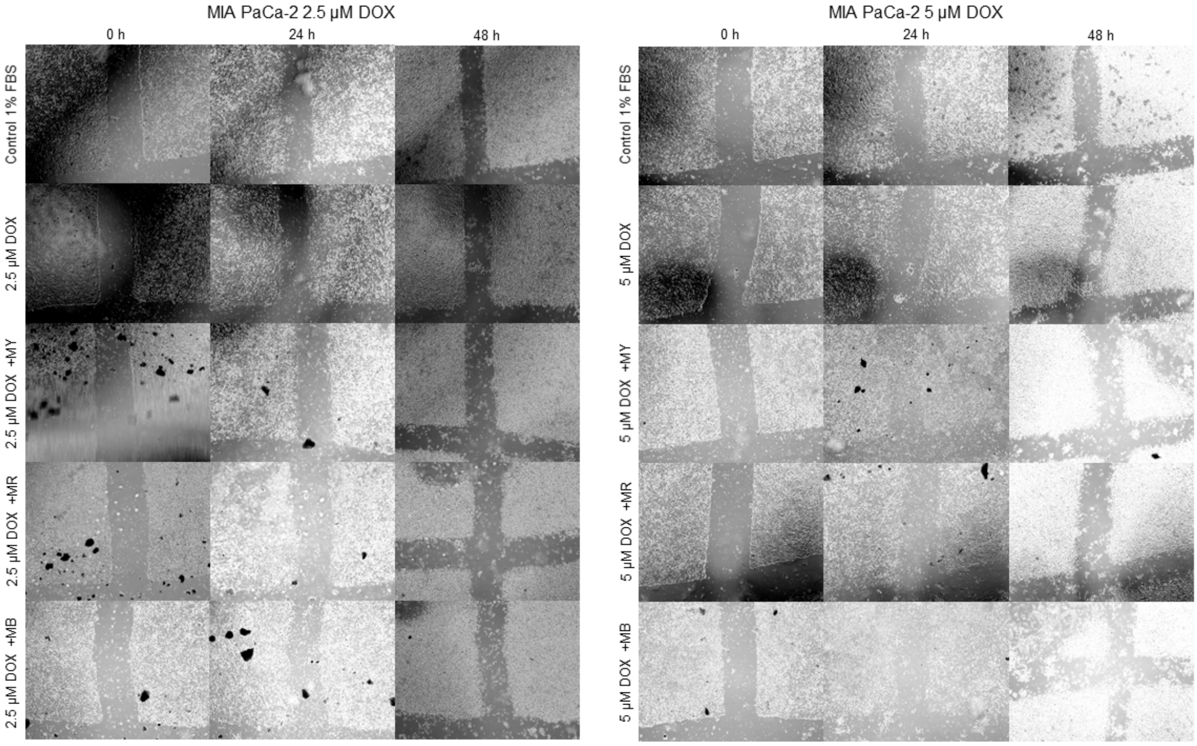

Supplementary Figure S3. Representative microscopy images of the wound healing assay in MIA PaCa-2 pancreatic cancer cells following doxorubicin (DOX) and maca co-treatment. Images show wound closure at 0, 24, and 48 h following treatment with (A) 2.5 μM DOX or (B) 5 μM DOX alone or in combination with Maca Yellow (MY), Maca Red (MR), or Maca Black (MB) (100 μg/mL).

Untreated cells served as the control. Representative micrographs illustrate treatment-dependent changes in wound closure compared to DOX monotherapy and control conditions.

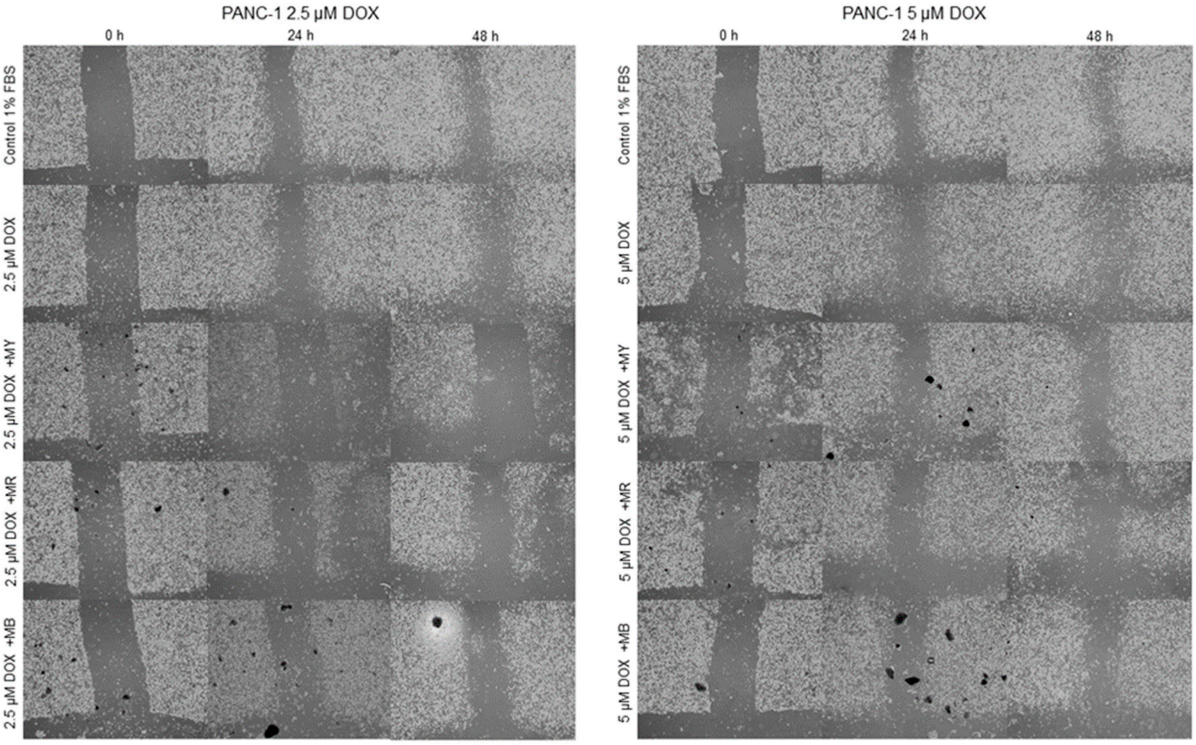

Supplementary Figure S4. Representative microscopy images of the wound healing assay in PANC-1 pancreatic cancer cells following doxorubicin (DOX) and maca co-treatment. Images show wound closure at 0, 24, and 48 h following treatment with (A) 2.5 µM DOX or (B) 5 µM DOX alone or in combination with Maca Yellow (MY), Maca Red (MR), or Maca Black (MB) (100 µg/mL). Untreated cells served as the control. Representative micrographs illustrate treatment-dependent changes in wound closure compared to DOX monotherapy and control conditions.

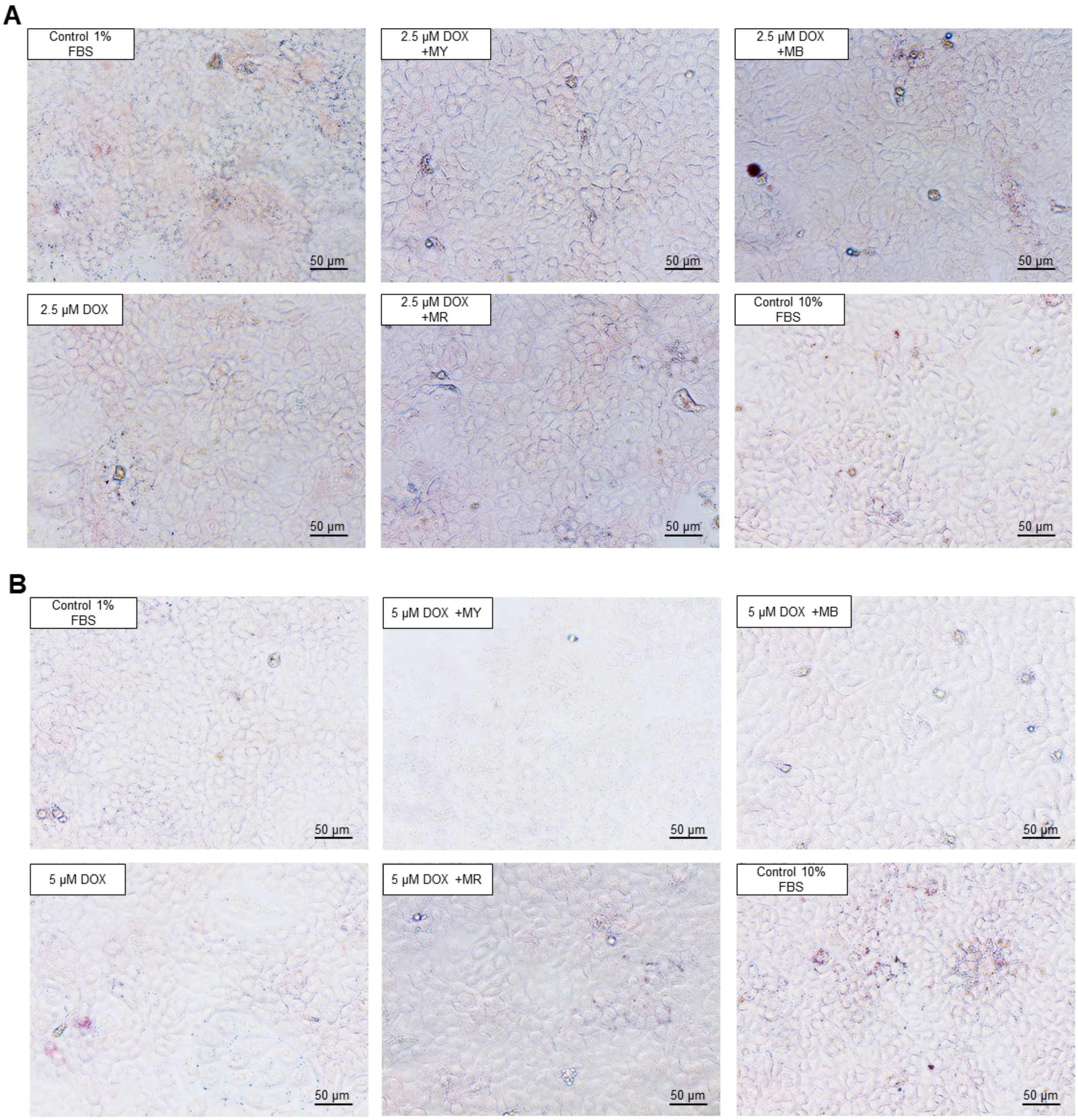

Supplementary Figure S5. Representative Oil Red O staining of lipid droplets (LDs) in BxPC-3 pancreatic cancer cells following doxorubicin (DOX) and maca co-treatment. Cells cultured in 1% FBS served as the control for reduced-serum conditions (Control 1% FBS), while 10% FBS represented standard growth conditions (Control 10% FBS). Treatment with (A) 2.5 µM DOX or (B) 5 µM DOX alone or in combination with Maca Yellow (MY), Maca Red (MR), or Maca Black (MB) (100 µg/mL) was performed. LDs were visualized by Oil Red O staining (red signal). Representative micrographs illustrate treatment-dependent changes in intracellular lipid accumulation, with combined DOX-maca treatment showing altered lipid droplet content compared to DOX alone and control conditions.

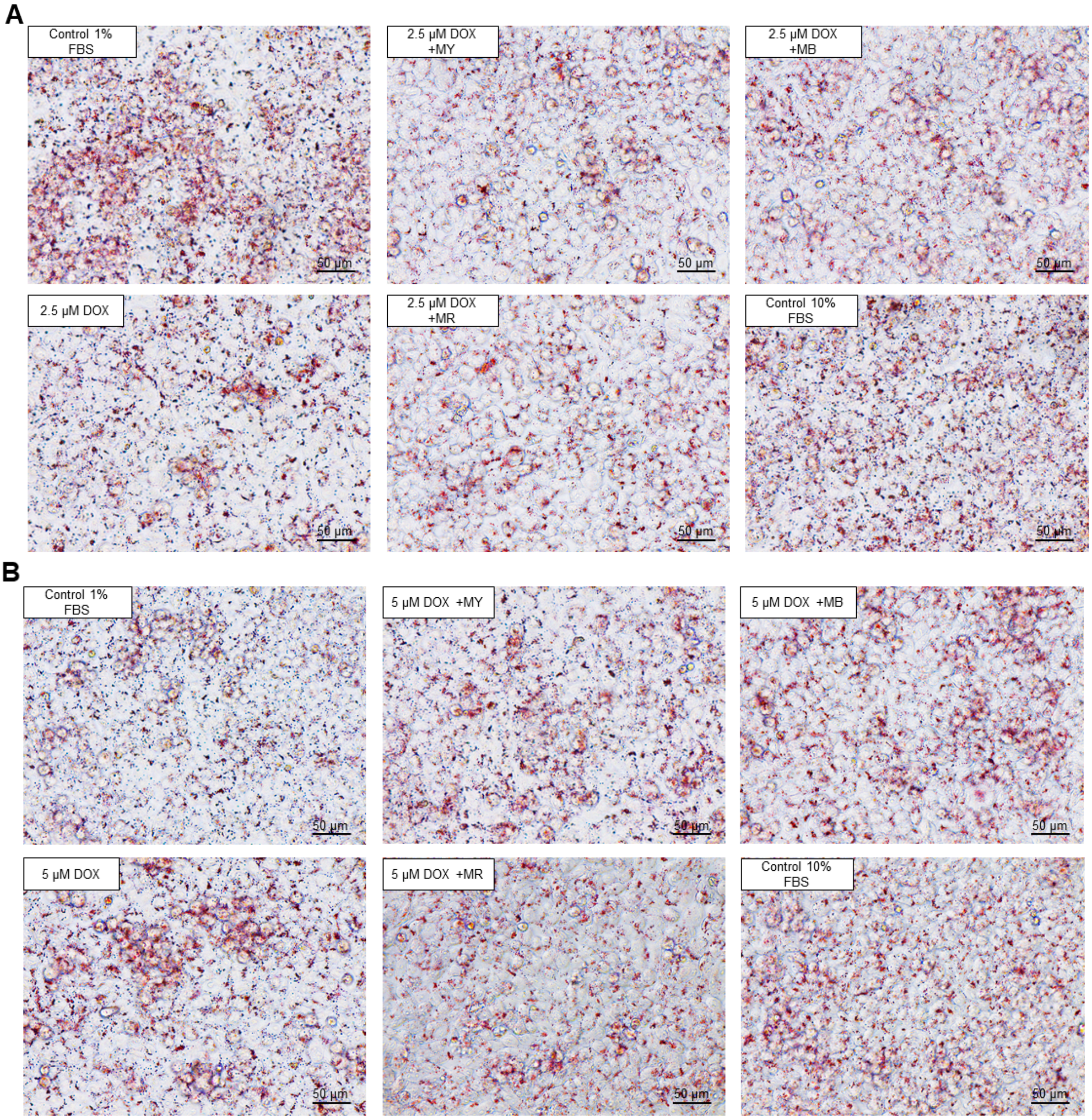

Supplementary Figure S6. Representative Oil Red O staining of lipid droplets (LDs) in MIA PaCa-2 pancreatic cancer cells following doxorubicin (DOX) and maca co-treatment. Cells cultured in 1% FBS served as the control for reduced-serum conditions (Control 1% FBS), while 10% FBS represented standard growth conditions (Control 10% FBS). Treatment with (A) 2.5 µM DOX or (B) 5 µM DOX alone or in combination with Maca Yellow (MY), Maca Red (MR), or Maca Black (MB) (100 µg/mL) was performed. LDs were visualized by Oil Red O staining (red signal). Representative micrographs illustrate treatment-dependent changes in intracellular lipid accumulation, with combined DOX-maca treatment showing altered lipid droplet content compared to DOX alone and control conditions.

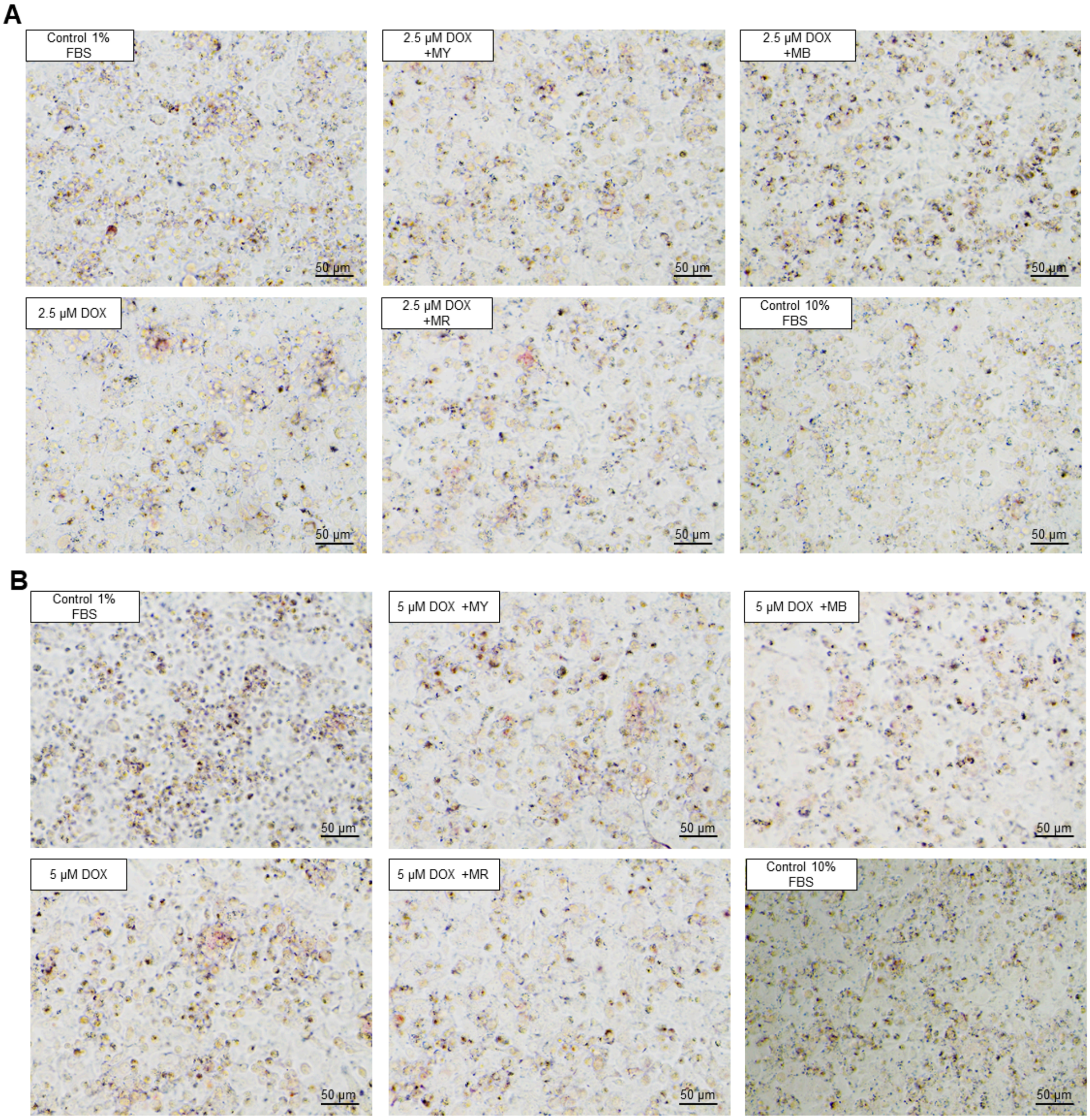

Supplementary Figure S7. Representative Oil Red O staining of lipid droplets (LDs) in PANC-1 pancreatic cancer cells following doxorubicin (DOX) and maca co-treatment. Cells cultured in 1% FBS served as the control for reduced-serum conditions (Control 1% FBS), while 10% FBS represented standard growth conditions (Control 10% FBS). Treatment with (A) 2.5 µM DOX or (B) 5 µM DOX alone or in combination with Maca Yellow (MY), Maca Red (MR), or Maca Black (MB) (100 µg/mL) was performed. LDs were visualized by Oil Red O staining (red signal). Representative micrographs illustrate treatment-dependent changes in intracellular lipid accumulation, with combined DOX-maca treatment showing altered lipid droplet content compared to DOX alone and control conditions.

Supplementary Table S1. Calculated IC_{25} and IC_{50} values for doxorubicin (DOX) in BxPC-3, MIA PaCa-2 and PANC-1 pancreatic cancer cells. Inhibitory concentrations were determined from dose-response curves after 24 h and 48 h of treatment. Values were derived from three independent biological experiments, each performed with four technical replicates.

Cell line	Substance	Incubation time	IC_{25} (μM)	IC_{50} (μM)
BxPC-3	DOX	24 h	2.80	5.21
BxPC-3	DOX	48 h	0.58	1.89
MIA PaCa-2	DOX	24 h	2.63	5.56
MIA PaCa-2	DOX	48 h	0.29	1.02
PANC-1	DOX	24 h	4.38	9.60
PANC-1	DOX	48 h	0.09	4.06

Supplementary Table S2. Complete Bliss independence, Highest Single Agent (HSA), and DOX enhancement analysis for combinations of doxorubicin and morphotype-specific maca preparations in BxPC-3, MIA PaCa-2, and PANC-1 pancreatic cancer cells after 24 h of treatment. Values represent means and corresponding standard deviations from three independent biological experiments.

**Cell line**	**DOX (μM)**	**Morphotype**	**Maca (μg/mL)**	**Observed viability (%)**	**Bliss expected (%)**	**ΔBliss**	**ΔBliss SD**	**HSA**	**HSA SD**	**Enhancement (%)**	**Enhancement SD**
BxPC-3	2.5	Yellow	10	81.96	82.29	0.33	5.64	-4.48	2.33	-5.78	1.97
BxPC-3	2.5	Yellow	25	80.62	77.53	-3.09	5.11	-3.13	4.59	-4.05	5.48
BxPC-3	2.5	Yellow	50	84.41	80.33	-4.08	10.77	-6.93	10.10	-8.94	12.83
BxPC-3	2.5	Yellow	100	79.31	79.41	0.10	4.47	-1.83	3.89	-2.36	4.50
BxPC-3	2.5	Yellow	200	43.65	66.77	23.12	5.83	33.84	5.72	43.67	7.16
BxPC-3	5	Yellow	10	68.94	68.82	-0.12	8.09	-4.14	6.73	-6.39	5.73
BxPC-3	5	Yellow	25	62.12	64.84	2.72	7.24	2.68	6.99	4.14	7.00
BxPC-3	5	Yellow	50	67.15	67.18	0.03	9.39	-2.35	8.79	-3.63	10.55
BxPC-3	5	Yellow	100	65.69	66.41	0.72	6.58	-0.89	6.26	-1.38	4.70
BxPC-3	5	Yellow	200	35.66	55.84	20.18	4.22	29.14	4.52	44.98	1.72
BxPC-3	2.5	Red	10	73.56	76.80	3.24	22.44	3.93	22.36	5.07	28.77
BxPC-3	2.5	Red	25	68.28	80.80	12.53	15.55	9.21	14.09	11.88	18.07
BxPC-3	2.5	Red	50	73.74	75.74	2.00	7.07	3.75	6.60	4.83	8.25
BxPC-3	2.5	Red	100	75.95	79.47	3.52	14.09	1.53	13.23	1.98	16.94
BxPC-3	2.5	Red	200	52.72	60.88	8.16	10.50	24.77	10.46	31.96	13.36
BxPC-3	5	Red	10	62.02	64.22	2.20	8.40	2.78	8.25	4.29	9.73
BxPC-3	5	Red	25	52.93	67.58	14.64	8.51	11.87	6.39	18.31	6.21
BxPC-3	5	Red	50	55.11	63.34	8.23	7.58	9.69	7.32	14.95	8.19
BxPC-3	5	Red	100	54.22	66.46	12.24	7.07	10.58	5.74	16.32	4.33
BxPC-3	5	Red	200	30.71	50.92	20.21	5.97	34.09	6.35	52.61	7.28
BxPC-3	2.5	Black	10	70.84	80.92	10.08	13.85	6.65	13.84	8.58	17.74
BxPC-3	2.5	Black	25	90.41	81.80	-8.61	14.65	-12.93	14.10	-16.68	18.04
BxPC-3	2.5	Black	50	84.86	81.86	-2.99	7.18	-7.37	5.81	-9.51	7.14
BxPC-3	2.5	Black	100	114.42	77.88	-36.54	3.72	-36.93	2.55	-47.66	1.76
BxPC-3	2.5	Black	200	67.79	62.25	-5.54	7.71	9.70	7.59	12.52	9.57
BxPC-3	5	Black	10	52.39	67.67	15.29	6.37	12.41	6.25	19.16	5.91
BxPC-3	5	Black	25	50.11	68.41	18.30	8.30	14.69	7.51	22.68	8.84
BxPC-3	5	Black	50	55.87	68.46	12.59	6.58	8.93	5.41	13.78	2.89
BxPC-3	5	Black	100	65.66	65.13	-0.53	7.08	-0.86	6.70	-1.33	5.99

BxPC-3	5	Black	200	40.17	52.06	11.89	4.61	24.63	4.98	38.01	3.22
MIA PaCa-2	2.5	Yellow	10	78.93	70.38	-8.55	6.76	1.06	5.30	1.32	2.12
MIA PaCa-2	2.5	Yellow	25	77.11	69.93	-7.18	5.14	2.88	5.15	3.60	1.68
MIA PaCa-2	2.5	Yellow	50	80.11	67.67	-12.44	5.60	-0.12	5.26	-0.15	1.77
MIA PaCa-2	2.5	Yellow	100	82.02	68.24	-13.78	5.46	-2.03	5.47	-2.53	2.41
MIA PaCa-2	2.5	Yellow	200	58.14	58.10	-0.04	5.93	14.50	5.86	27.32	4.17
MIA PaCa-2	5	Yellow	10	47.95	43.33	-4.62	5.89	1.29	5.47	2.63	4.10
MIA PaCa-2	5	Yellow	25	48.45	43.05	-5.40	4.99	0.79	5.19	1.61	1.85
MIA PaCa-2	5	Yellow	50	48.30	41.66	-6.64	5.24	0.94	5.33	1.91	3.12
MIA PaCa-2	5	Yellow	100	45.43	42.01	-3.42	4.73	3.81	4.97	7.75	0.86
MIA PaCa-2	5	Yellow	200	29.70	35.77	6.07	4.95	19.55	5.17	39.69	5.99
MIA PaCa-2	2.5	Red	10	73.21	70.73	-2.48	4.77	6.78	5.04	8.48	1.74
MIA PaCa-2	2.5	Red	25	73.83	72.26	-1.57	4.72	6.16	4.95	7.70	1.15
MIA PaCa-2	2.5	Red	50	72.93	69.16	-3.77	4.74	7.06	4.96	8.83	1.36
MIA PaCa-2	2.5	Red	100	73.83	73.45	-0.38	4.96	6.16	4.89	7.70	0.60
MIA PaCa-2	2.5	Red	200	56.53	59.75	3.22	6.34	18.16	6.12	29.33	5.46
MIA PaCa-2	5	Red	10	48.44	43.54	-4.90	4.89	0.80	5.17	1.63	1.64
MIA PaCa-2	5	Red	25	47.12	44.48	-2.64	4.92	2.12	5.16	4.30	2.24
MIA PaCa-2	5	Red	50	47.51	42.57	-4.94	4.82	1.73	5.11	3.52	1.53
MIA PaCa-2	5	Red	100	46.21	45.22	-0.99	4.93	3.04	5.03	6.17	1.33
MIA PaCa-2	5	Red	200	33.85	36.78	2.93	4.25	15.39	4.58	31.26	2.48
MIA PaCa-2	2.5	Black	10	92.16	67.29	-24.88	6.45	-12.17	5.78	-15.22	2.37
MIA PaCa-2	2.5	Black	25	88.38	73.48	-14.90	6.23	-8.39	5.66	-10.49	2.40
MIA PaCa-2	2.5	Black	50	84.50	66.00	-18.50	5.84	-4.51	5.53	-5.64	2.34
MIA PaCa-2	2.5	Black	100	80.66	67.02	-13.64	6.61	-0.67	5.36	-0.83	2.13
MIA PaCa-2	2.5	Black	200	61.03	49.47	-11.56	6.42	0.82	6.85	23.71	3.92
MIA PaCa-2	5	Black	10	55.45	41.42	-14.03	5.60	-6.21	5.54	-12.61	1.62
MIA PaCa-2	5	Black	25	52.82	45.23	-7.59	5.48	-3.58	5.37	-7.27	1.18
MIA PaCa-2	5	Black	50	50.14	40.63	-9.51	5.20	-0.90	5.33	-1.82	2.38
MIA PaCa-2	5	Black	100	46.00	41.26	-4.75	5.37	3.24	5.07	6.57	1.89
MIA PaCa-2	5	Black	200	30.87	30.45	-0.41	4.20	18.37	4.32	37.31	0.83
PANC-1	2.5	Yellow	10	91.50	84.13	-7.37	6.31	-6.40	4.53	-7.53	1.24
PANC-1	2.5	Yellow	25	90.91	90.16	-0.75	5.08	-5.82	4.93	-6.84	2.62
PANC-1	2.5	Yellow	50	87.06	82.16	-4.91	5.33	-1.97	5.07	-2.32	3.18
PANC-1	2.5	Yellow	100	77.61	88.11	10.50	7.31	7.48	6.76	8.80	6.35
PANC-1	2.5	Yellow	200	43.90	72.75	28.86	10.44	41.20	10.49	48.41	11.67
PANC-1	5	Yellow	10	67.57	65.80	-1.76	4.30	-1.01	2.58	-1.51	3.10
PANC-1	5	Yellow	25	70.87	70.52	-0.35	2.79	-4.31	2.72	-6.48	3.32
PANC-1	5	Yellow	50	64.66	64.26	-0.40	5.07	1.89	4.88	2.85	6.97
PANC-1	5	Yellow	100	65.60	68.92	3.31	2.87	0.96	1.94	1.44	1.79
PANC-1	5	Yellow	200	34.60	56.91	22.31	10.53	31.96	10.50	48.02	15.66
PANC-1	2.5	Red	10	84.20	86.66	2.46	5.28	0.89	5.19	1.05	3.55
PANC-1	2.5	Red	25	79.46	93.72	14.27	6.04	5.64	5.10	6.63	3.55
PANC-1	2.5	Red	50	79.46	87.63	8.18	5.11	5.64	4.80	6.63	2.92
PANC-1	2.5	Red	100	77.78	92.11	14.33	6.31	7.31	5.56	8.60	4.46
PANC-1	2.5	Red	200	48.81	71.43	22.63	14.26	35.14	14.19	42.65	15.87
PANC-1	5	Red	10	60.45	67.79	7.33	2.54	6.10	2.46	9.17	2.97
PANC-1	5	Red	25	65.99	73.31	7.31	4.62	0.57	3.98	0.85	5.52
PANC-1	5	Red	50	60.86	68.54	7.69	2.19	5.70	1.79	8.56	1.52
PANC-1	5	Red	100	65.83	72.04	6.21	2.69	0.73	1.61	1.09	0.76

PANC-1	5	Red	200	52.27	55.87	3.61	3.40	14.29	2.18	21.47	2.53
PANC-1	2.5	Black	10	82.07	91.96	9.90	6.11	3.03	4.63	3.56	2.36
PANC-1	2.5	Black	25	78.08	92.35	14.28	6.47	7.02	5.09	8.25	3.59
PANC-1	2.5	Black	50	75.50	83.85	8.35	4.80	9.60	4.14	11.28	1.17
PANC-1	2.5	Black	100	74.66	92.35	17.69	5.30	10.43	4.15	12.26	1.31
PANC-1	2.5	Black	200	55.24	68.68	13.43	4.59	25.46	3.92	35.08	3.87
PANC-1	5	Black	10	61.95	71.93	9.98	3.66	4.61	2.10	6.92	2.23
PANC-1	5	Black	25	67.86	72.24	4.37	3.56	-1.31	1.91	-1.96	1.66
PANC-1	5	Black	50	62.69	65.59	2.89	2.98	3.87	2.26	5.81	2.54
PANC-1	5	Black	100	63.64	72.24	8.59	3.64	2.92	2.71	4.38	3.39
PANC-1	5	Black	200	48.32	53.72	5.40	3.02	18.24	3.03	27.40	4.08
